# Global trends and regional differences in disease burden of stroke among children: a trend analysis based on the global burden of disease study 2019

**DOI:** 10.1186/s12889-023-17046-z

**Published:** 2023-10-27

**Authors:** Min Du, Donghua Mi, Min Liu, Jue Liu

**Affiliations:** 1 Department of Epidemiology and Biostatistics, School of Public Health, Peking University, No.38, Xueyuan Road, Haidian District, Beijing, 100191 China; 2 Beijing Tiantan Hospital, China National Clinical Research Center for Neurological Diseases, Capital Medical University, 119 South Fourth Ring West Road, Fengtai District, Beijing, 100871 China; 3 Institute for Global Health and Development, Peking University, No.5, Yiheyuan Road, Haidian District, Beijing, 100871 China

**Keywords:** Stroke, Incidence, Prevalence, Mortality, Children

## Abstract

**Background:**

Stroke is a major cause of acute neurological symptoms in children with significant long-term neurological sequelae. However, data of diseases burden on stroke among children was lack. We aimed to be dedicated to analyze and compare global trends as well as regional and sociodemographic differences in stroke prevalence, incidence, mortality and disability-adjusted life-years (DALYs) among children aged 0 ~ 14 years.

**Method:**

We obtained data on annual number of incident strokes, prevalent strokes, deaths, and DALYs, age-standardized incidence rates (ASIRs), prevalence rates (ASPRs), mortality rates (ASMRs) and DALY rates (ASDRs) of stroke among individuals aged 14 years and younger during 1990–2019 from the 2019 Global Burden of Disease Study. To quantify the temporal trends, we calculated changes (%) in number, and used joinpoint regression analysis to identify the average annual percentage changes (AAPCs) of age standardized rates.

**Result:**

Globally, the incident strokes and prevalent strokes increased by 18.51% and 31.97%, respectively, but DALYs due to stroke and deaths due to stroke decreased by 60.18% and 65.03%, respectively, from 1990 to 2019. During the same period, ASIR increased by 0.21% (95%CI: 0.17, 0.24) from 18.02 to 100,000 population in 1990 to 19.11 per 100,000 in 2019; ASPR increased by 0.66% (95%CI: 0.36, 0.96) from 68.88 to 100,000 population in 1990 to 81.35 per 100,000 in 2019; while ASMR (AAPC= -3.94; 95%CI: -4.07, -3.81) and ASDR (AAPC= -3.50; 95%CI: -3.64, -3.36) both decreased. In 2019, the highest age standardized incidence, prevalence, mortality, and DALY rates all occurred in low sociodemographic index (SDI) regions. The greatest increase of age standardized incidence rate (AAPC = 0.21; 95%CI: 0.18, 0.25) and prevalence rate (AAPC = 1.15; 95%CI: 0.34, 1.96) both were in high SDI regions. Eastern Sub-Saharan Africa had the highest ASIR and ASPR in 2019, and Oceania had the highest ASMR and ASDR in 2019 across 21 GBD regions. High-income North America had the largest increase in ASIR (AAPC = 0.63; 95%CI: 0.59, 0.66) and ASPR (AAPC = 1.58; 95%CI: 0.54, 2.63). Against the overall decreasing trend of ASMR, an increasing trend of ASMR was found in Zimbabwe (AAPC = 0.91; 95%CI: 0.44, 1.37) and Botswana (AAPC = 0.74; 95%CI: 0.02, 1.47).

**Conclusion:**

The overall increasing stroke incidence and prevalence indicated that prevention and management of stroke among younger population should be critical in the future. Despite stroke mortality with falling trend worldwide, specific countries or territories present worrying increase in stroke mortality. Without urgent implementation of effective primary prevention strategies, the stroke burden of children will probably continue to grow across the world, particularly in high-SDI countries.

**Supplementary Information:**

The online version contains supplementary material available at 10.1186/s12889-023-17046-z.

## Introduction

A large number of deaths and disabilities were from stroke, remaining the second-leading cause of death and the third-leading cause of disability in 2019 worldwide [1]. There were more than 100 million prevalent stroke cases and disability-adjusted life-years (DALYs) due to stroke, and 12.2 million incident stroke cases with 6.55 million deaths due to stroke in 2019 [1]. Stroke is primarily considered as a neurological disorder of the elderly, while in recent years, the increasing number of studies reported that stroke occurred in younger people, even children.

Childhood strokes further are subdivided into three groups: perinatal stroke, stroke in infancy and early childhood and adolescent stroke [2, 3]. Previous studies reported that the prevalence of strokes ranged from 2.9 to 28% in children [4, 5]. A nationwide Swedish registers identified 1606 individuals aged less than 18 years old with ischemic stroke between 1969 and 2016 [6]. Sharma et al. reported that 48 children had haemorrhagic stroke [7]. Stroke results from a multifactorial background in both children and in adults. However, it differs in children, compared that in adults. Previous studies showed that central nervous system infections, vitamin K deficiency-related bleeding disorder, inherited coagulation disorders, and arteriovenous malformations were the most common risk factors for childhood stroke [4, 7–9]. Compare with genetic risk factors, non-genetic risk factors, such as environmental factors (which are common in adults) seem to have low or no clinical relevance in children [10, 11].

Disease patterns, population distribution, and sociodemographic factors continue to change the stroke epidemic across the world. Previous publications on the stroke burden in the core Global Burden of Disease (GBD) study did not report analyses among children. In the present study, we retrieved detailed data on the prevalence, incidence, mortality and DALYs of stroke among children aged 14 years and younger from the 2019 GBD study, which provided a global landscape of the epidemic trend of stroke.

## Method

### Data source

We conducted a post hoc analysis of the GBD 2019. The Institute for Health Metrics and Evaluation at the University of Washington, USA, coordinated the GBD study as a systematic and scientific effort on quantifying the comparative magnitude of health losses due to diseases at sex, age, region and country level over time [12]. In short, stroke was defined as rapidly developing clinical signs of disturbance of cerebral function (usually focal) lasting more than 24 h or leading to death based on WHO clinical criteria [13]. As GBD 2019 only provided data of children aged 14 years and younger age among children younger than 18 years old, we obtained 19-year (1990–2019) annual absolute number, age-standardized incidence rates (ASIRs), prevalence rates (ASPRs), mortality rates (ASMRs) and DALY rates (ASDRs) of stroke in children aged 14 years and younger by sex, region, and country from the website Global Health Data Exchange [12]. The detail methods are shown in elsewhere [1]. In short, deaths were estimated using vital registration and verbal autopsy data in the cause of death ensemble modelling (CODEm) framework. Incidence, prevalence, and mortality were generated by using the Bayesian meta-regression (DisMod-MR 2.1) modelling tool based on all available high-quality related data [1]. As this study used model data, ethical review and informed consent were not required.

### Sociodemographic index, regions and demographics

The sociodemographic index (SDI) as a composite indicator, was the weighted geometric mean of three rescaled components including income per capita, average educational attainment in population aged 15 years or older and total fertility rates under age 25 years [14]. It ranged from zero to one, with a higher value reflected a theoretical higher level of development status relevant to health outcomes. All 204 countries or territories were classified into five SDI regions, including low, low-middle, middle, high-middle and high SDI regions.

According to epidemiological homogeneity and geographical contiguity, all 204 countries or territories were separated into 21 GBD regions, including High-income Asia Pacific, Central Asia and others [15].

### Statistical analysis

The absolute number with 95% uncertainty intervals (UIs) of prevalent strokes, incident strokes, deaths due to stroke and DALYs due to stroke, and age-standardized rate with 95% UIs were used to indicate epidemic status [16]. The relative changes of absolute number from 1990 to 2019 were calculated, defining as

$$\frac{{\text{n}\text{u}\text{m}\text{b}\text{e}\text{r}}_{2019}-{\text{n}\text{u}\text{m}\text{b}\text{e}\text{r}}_{1990}}{{\text{n}\text{u}\text{m}\text{b}\text{e}\text{r}}_{1990}}\times 100\text{\%}$$

Average annual percentage change (AAPC) as a summary and widely used measure of the trend of age-standardized rate, was calculated by using Joinpoint regression analysis [17–19]. Joinpoint regression analysis assessed the trend of the ASR over a specified time interval on a logarithmic scale. In this study, number of joinpoints ranged from zero to five was added. Joinpoint regression analysis tested every group for significance using a Monte Carlo permutation method, selected the best suitable model, and estimated AAPC with 95% confidential interval (CI) which provides a single summary number for the trend over the past 30 years [18, 20]. Joinpoint regression analyses were performed by joinpoint regression program (Version 4.9.1.0 - Nov 2022; Statistical Methodology and Applications Branch, Surveillance Research Program, National Cancer Institute).

To investigate the potential influence factors of the AAPC at the national level, we applied Pearson correlation analysis to assess the correlation of AAPCs with age-standardized incidence (ASIR), prevalence (ASPR), mortality (ASMR), and DALY rates (ASDR) as well as with SDI values (2019) in 204 countries and territories. Polynomial curves were used to present results of analysis. Except Joinpoint regression analyses, other analysis was conducted by R (version 4.1.0). A two-tailed P value less than 0.05 was statistically significant. This manuscript conforms to the STROBE guidelines for observational cohort studies [21].

## Result

### Global trends in stroke prevalence, incidence, mortality and DALY rate

In 2019, there were 374,509.14 (95% UI: 256,946.45, 531,678.85) incident strokes (Table 1) and 1,594,311.22 (95% UI: 1,205,044.34, 2,110,296.06) prevalent strokes (Table S1), 1,678,539.47 (95% UI: 1,390,409.59, 2,034,897.27) DALYs due to stroke (Table S2), and 16,104.92 (95% UI: 13,102.73, 20,177.15) deaths due to stroke (Table 2). The absolute number of incident strokes globally increased by 18.51% from 1990 to 2019, prevalent strokes increased by 31.97%, but DALYs due to stroke decreased by 60.18%, deaths due to stroke decreased by 65.03%. Similarly, age-standardized rates both increased by 0.21% (95% CI: 0.17, 0.24) for incidence from 18.02 to 100,000 population in 1990 to 19.11 per 100,000 in 2019; by 0.66% (95% CI: 0.36, 0.96) for prevalence from 68.88 to 100,000 population in 1990 to 81.35 per 100,000 in 2019. The age-standardized mortality rate (AAPC= -3.94; 95% CI: -4.07, -3.81) and age-standardized DALY rate (AAPC= -3.50; 95% CI: -3.64, -3.36) both decreased.

**Table 1:** The incident strokes and age-standardized incidence rate and their temporal change among children (0–14 years), 1990 − 2019

	Incident strokes (95% UI)			Age-standardized incidence rate (95% UI), per 100,000 population		
Characteristics	1990	2019	Percentage change (%)	1990	2019	AAPC (95% CI)
**Global**	316012.27 (215253.42, 451640.07)	374509.14 (256946.45, 531678.85)	18.51%	18.02 (12.27, 25.75)	19.11 (13.11, 27.13)	0.21 (0.17, 0.24)
**Sex**						
Female	175677.19 (247641.97, 120810.58)	213330.85 (297243.63, 147852.03)	21.43%	29.02 (3.79, 14.16)	31.35 (4.02, 15.60)	0.31 (0.28, 0.34)
Male	140335.08 (199980.65, 94379.08)	161178.29 (230475.70, 108811.99)	14.85%	22.20 (2.99, 10.48)	22.78 (3.07, 10.76)	0.09 (0.03, 0.16)
**Socio-demographic index**						
Low SDI	56461.00 (38992.80, 78919.56)	113961.88 (78429.10, 159704.68)	101.84%	23.33 (16.11, 32.61)	24.10 (16.59, 33.77)	0.13 (0.08, 0.18)
Low-middle SDI	86394.55 (59027.51, 122807.97)	104553.39 (71614.19, 148163.65)	21.02%	19.05 (13.02, 27.09)	19.97 (13.68, 28.30)	0.17 (0.14, 0.20)
Middle SDI	106886.88 (73005.40, 151522.75)	100441.23 (68811.18, 142962.98)	-6.03%	18.39 (12.56, 26.06)	18.14 (12.43, 25.82)	-0.04 (-0.09, 0.01)
High-middle SDI	47204.95 (31793.34, 67894.72)	36368.74 (24551.18, 53071.53)	-22.96%	15.55 (10.47, 22.37)	14.83 (10.01, 21.64)	-0.17 (-0.26, -0.08)
High SDI	18871.48 (12377.84, 28253.23)	18933.42 (12352.96, 28343.89)	0.33%	10.93 (7.17, 16.36)	11.60 (7.57, 17.36)	0.21 (0.18, 0.25)
**GBD region**						
Andean Latin America	2825.76 (1952.52, 3931.35)	3357.30 (2306.58, 4729.78)	18.81%	18.78 (12.98, 26.13)	18.56 (12.75, 26.14)	-0.03 (-0.09, 0.03)
Australasia	504.16 (318.76, 757.07)	573.11 (371.69, 870.44)	13.68%	10.99 (6.95, 16.50)	10.45 (6.78, 15.87)	-0.16 (-0.21, -0.12)
Caribbean	2424.75 (1668.87, 3387.52)	2496.52 (1701.01, 3512.12)	2.96%	21.24 (14.62, 29.68)	21.37 (14.56, 30.06)	0.02 (0.01, 0.03)
Central Asia	3271.40 (2218.74, 4674.09)	3372.14 (2258.24, 4887.71)	3.08%	13.11 (8.89, 18.73)	12.54 (8.40, 18.17)	-0.15 (-0.18, -0.12)
Central Europe	3524.97 (2376.78, 5131.10)	1961.18 (1310.21, 2905.79)	-44.36%	12.18 (8.21, 17.73)	11.12 (7.43, 16.48)	-0.30 (-0.34, -0.26)
Central Latin America	13234.05 (9174.52, 18489.75)	12861.43 (8840.98, 18288.05)	-2.82%	20.65 (14.31, 28.85)	19.67 (13.52, 27.97)	-0.16 (-0.18, -0.15)
Central Sub-Saharan Africa	6279.53 (4343.33, 8740.40)	13018.81 (8864.15, 18345.84)	107.32%	24.31 (16.81, 33.83)	22.82 (15.54, 32.16)	-0.22 (-0.23, -0.20)
East Asia	53892.35 (34926.26, 78754.84)	32176.19 (20577.57, 48245.07)	-40.30%	16.09 (10.43, 23.51)	13.83 (8.84, 20.73)	-0.53 (-0.60, -0.47)
Eastern Europe	7188.62 (4880.27, 10437.50)	5696.03 (3916.18, 8176.97)	-20.76%	13.97 (9.49, 20.29)	15.37 (10.57, 22.06)	0.33 (0.28, 0.39)
Eastern Sub-Saharan Africa	25919.99 (18058.56, 36370.10)	51004.93 (34821.74, 72087.07)	96.78%	28.68 (19.98, 40.25)	28.89 (19.73, 40.84)	0.04 (0.02, 0.07)
High-income Asia Pacific	3344.87 (2171.15, 5078.53)	2185.89 (1393.52, 3327.37)	-34.65%	9.49 (6.16, 14.41)	9.36 (5.97, 14.25)	-0.02 (-0.20, 0.17)
High-income North America	6423.48 (4012.71, 9908.91)	8308.54 (5353.31, 12689.84)	29.35%	10.46 (6.53, 16.14)	12.54 (8.08, 19.15)	0.63 (0.59, 0.66)
North Africa and Middle East	27602.80 (19047.35, 38577.35)	35116.60 (24206.07, 49369.62)	27.22%	19.21 (13.25, 26.84)	19.97 (13.77, 28.08)	0.14 (0.11, 0.17)
Oceania	488.89 (339.12, 676.13)	899.89 (624.19, 1257.61)	84.07%	18.61 (12.91, 25.73)	18.63 (12.92, 26.04)	0.01 (0.00, 0.02)
South Asia	80708.57 (54511.03, 114912.62)	97871.28 (66360.11, 139791.43)	21.27%	18.39 (12.42, 26.18)	18.94 (12.84, 27.05)	0.11 (0.08, 0.14)
Southeast Asia	30833.44 (21181.59, 43505.77)	30777.36 (21198.62, 43623.16)	-0.18%	17.90 (12.30, 25.26)	18.24 (12.56, 25.85)	0.07 (0.04, 0.10)
Southern Latin America	1636.32 (1090.67, 2405.95)	1508.60 (987.34, 2221.14)	-7.81%	10.96 (7.31, 16.11)	10.12 (6.62, 14.90)	-0.27 (-0.28, -0.25)
Southern Sub-Saharan Africa	5588.82 (3841.48, 7804.12)	6245.14 (4272.43, 8749.48)	11.74%	27.43 (18.85, 38.30)	26.45 (18.09, 37.05)	-0.10 (-0.21, 0.01)
Tropical Latin America	12467.17 (8700.65, 17460.29)	10413.03 (7199.49, 14644.44)	-16.48%	23.11 (16.13, 32.37)	20.95 (14.48, 29.46)	-0.32 (-0.43, -0.20)
Western Europe	7365.25 (4753.93, 11105.14)	6596.67 (4272.55, 9903.40)	-10.44%	10.37 (6.69, 15.63)	9.59 (6.21, 14.39)	-0.27 (-0.29, -0.25)
Western Sub-Saharan Africa	20487.10 (14050.54, 28935.23)	48068.49 (32700.87, 67894.52)	134.63%	23.33 (16.00, 32.95)	24.24 (16.49, 34.23)	0.14 (0.11, 0.18)

Note: AAPC, average annual percentage change; CI, confidence interval; GBD, Global Burden of Disease; SDI, socio-demographic index; UI, uncertainty interval

**Table 2:** The deaths and age-standardized morality rate and their temporal change among children (0–14 years), 1990 − 2019

	Deaths (95% UI)			Age-standardized mortality rate (95% UI), per 100,000 population		
**Characteristics**	1990	**2019**	**Percentage change (%)**	**1990**	**2019**	**AAPC (95% CI)**
**Global**	46048.59 (34950.13, 60964.89)	16104.92 (13102.73, 20177.15)	-65.03%	2.63 (1.99, 3.48)	0.82 (0.67, 1.03)	-3.94 (-4.07, -3.81)
**Sex**						
Female	20190.14 (27752.63, 15539.46)	6852.35 (8426.85, 5585.16)	-66.06%	2.37 (1.82, 3.25)	0.72 (0.59, 0.89)	-4.01 (-4.23, -3.79)
Male	25858.45 (34585.53, 18785.29)	9252.57 (11850.09, 7396.65)	-64.22%	2.87 (2.09, 3.84)	0.91 (0.73, 1.17)	-3.91 (-4.03, -3.78)
**Socio-demographic index**						
Low SDI	6586.11 (4555.38, 9666.33)	6408.57 (4927.95, 8716.14)	-2.70%	2.72 (1.88, 3.99)	1.36 (1.04, 1.84)	-2.37 (-2.51, -2.22)
Low-middle SDI	10260.33 (7463.43, 14993.25)	4319.27 (3498.12, 5241.50)	-57.90%	2.26 (1.65, 3.31)	0.82 (0.67, 1.00)	-3.43 (-3.54, -3.33)
Middle SDI	23429.87 (15558.85, 30150.39)	4492.89 (3420.93, 6010.64)	-80.82%	4.03 (2.68, 5.19)	0.81 (0.62, 1.09)	-5.42 (-5.68, -5.15)
High-middle SDI	4736.73 (3596.16, 5863.53)	607.81 (521.93, 724.50)	-87.17%	1.56 (1.18, 1.93)	0.25 (0.21, 0.30)	-6.24 (-6.50, -5.98)
High SDI	999.40 (929.74, 1090.14)	254.18 (220.85, 293.25)	-74.57%	0.58 (0.54, 0.63)	0.16 (0.14, 0.18)	-4.38 (-4.69, -4.06)
**GBD region**						
Andean Latin America	407.32 (305.14, 502.81)	93.26 (71.98, 120.74)	-77.10%	2.71 (2.03, 3.34)	0.52 (0.40, 0.67)	-5.61 (-5.83, -5.40)
Australasia	11.74 (9.97, 14.04)	2.79 (2.13, 3.62)	-76.22%	0.26 (0.22, 0.31)	0.05 (0.04, 0.07)	-5.48 (-6.21, -4.74)
Caribbean	410.54 (250.35, 593.67)	243.39 (147.97, 372.53)	-40.71%	3.60 (2.19, 5.20)	2.08 (1.27, 3.19)	-1.87 (-2.05, -1.68)
Central Asia	113.25 (84.82, 134.25)	35.54 (28.58, 44.79)	-68.62%	0.45 (0.34, 0.54)	0.13 (0.11, 0.17)	-4.22 (-4.64, -3.80)
Central Europe	234.79 (207.03, 268.51)	35.65 (29.41, 42.51)	-84.82%	0.81 (0.72, 0.93)	0.20 (0.17, 0.24)	-4.67 (-5.02, -4.32)
Central Latin America	895.29 (790.63, 982.72)	333.12 (241.62, 470.27)	-62.79%	1.40 (1.23, 1.53)	0.51 (0.37, 0.72)	-3.38 (-3.61, -3.16)
Central Sub-Saharan Africa	778.70 (408.49, 1281.09)	439.17 (291.66, 651.95)	-43.60%	3.01 (1.58, 4.96)	0.77 (0.51, 1.14)	-4.61 (-4.84, -4.39)
East Asia	11217.46 (7672.33, 13803.73)	717.55 (565.67, 996.28)	-93.60%	3.35 (2.29, 4.12)	0.31 (0.24, 0.43)	-7.96 (-8.46, -7.45)
Eastern Europe	110.56 (82.30, 122.94)	37.50 (29.26, 43.90)	-66.08%	0.21 (0.16, 0.24)	0.10 (0.08, 0.12)	-2.78 (-4.15, -1.39)
Eastern Sub-Saharan Africa	1814.85 (1166.04, 2620.98)	1111.01 (751.58, 1692.79)	-38.78%	2.01 (1.29, 2.90)	0.63 (0.43, 0.96)	-3.91 (-4.05, -3.78)
High-income Asia Pacific	295.50 (252.78, 341.17)	23.65 (19.46, 28.30)	-92.00%	0.84 (0.72, 0.97)	0.10 (0.08, 0.12)	-7.04 (-7.56, -6.51)
High-income North America	350.60 (328.29, 390.66)	166.74 (147.01, 190.03)	-52.44%	0.57 (0.53, 0.64)	0.25 (0.22, 0.29)	-2.80 (-3.35, -2.26)
North Africa and Middle East	14671.77 (8452.31, 20618.15)	3382.93 (2314.27, 4890.20)	-76.94%	10.21 (5.88, 14.35)	1.92 (1.32, 2.78)	-5.61 (-5.85, -5.38)
Oceania	92.57 (51.16, 151.35)	160.37 (89.37, 261.09)	73.24%	3.52 (1.95, 5.76)	3.32 (1.85, 5.41)	-0.23 (-0.51, 0.06)
South Asia	5634.07 (4144.96, 7580.72)	2936.10 (2355.96, 3572.42)	-47.89%	1.28 (0.94, 1.73)	0.57 (0.46, 0.69)	-2.86 (-3.02, -2.70)
Southeast Asia	4290.68 (2714.36, 6888.18)	1471.50 (1142.80, 1819.66)	-65.70%	2.49 (1.58, 4.00)	0.87 (0.68, 1.08)	-3.53 (-3.77, -3.28)
Southern Latin America	170.00 (145.13, 197.86)	36.15 (29.12, 43.65)	-78.74%	1.14 (0.97, 1.33)	0.24 (0.20, 0.29)	-5.26 (-5.43, -5.09)
Southern Sub-Saharan Africa	170.70 (99.26, 250.96)	89.91 (62.44, 124.46)	-47.33%	0.84 (0.49, 1.23)	0.38 (0.26, 0.53)	-2.60 (-3.10, -2.10)
Tropical Latin America	677.02 (555.14, 834.56)	192.36 (157.54, 232.74)	-71.59%	1.26 (1.03, 1.55)	0.39 (0.32, 0.47)	-3.95 (-4.23, -3.68)
Western Europe	376.68 (347.07, 424.90)	60.91 (49.26, 71.37)	-83.83%	0.53 (0.49, 0.60)	0.09 (0.07, 0.10)	-6.02 (-6.29, -5.74)
Western Sub-Saharan Africa	3324.49 (2361.44, 4897.85)	4535.32 (3284.69, 6778.78)	36.42%	3.79 (2.69, 5.58)	2.29 (1.66, 3.42)	-1.71 (-1.93, -1.49)

Note: AAPC, average annual percentage change; CI, confidence interval; GBD, Global Burden of Disease; SDI, socio-demographic index; UI, uncertainty interval

### Regional trend in stroke prevalence, incidence, mortality and DALY rate in five SDI regions

The number of incident strokes, prevalent strokes, stroke-related deaths and stroke-related DALYs in 2019 and their changes during the past three decades all were highest in low SDI region (Table S1-S2, Tables 1 and 2). In 2019, the highest age-standardized incidence, prevalence, mortality, and DALY rates occurred in low SDI regions (Table S1-S2, Tables 1 and 2). The greatest increase of age-standardized incidence (AAPC = 0.21; 95% CI: 0.18, 0.25) and prevalence (AAPC = 1.15; 95% CI: 0.34, 1.96) both were in high SDI regions (Table S1, Table 1). The age-standardized mortality rate and DALY rate decreased across five SDI regions; the greatest decline was in high-middle SDI regions (AAPC= -6.24; 95% CI: -6.50, -5.98) for mortality and in middle SDI regions (AAPC= -4.89; 95% CI: -5.25, -4.54) for DALY rate (Table S2, Table 2).

### Regional trend in stroke prevalence, incidence, mortality and DALY rate in 21 GBD regions

For GBD regions, there were nine regions with more than ten thousand incident strokes and six regions with more than one hundred thousand prevalent strokes in 2019, which of them, South Asia suffered the severest threat with 97,871.28 incident cases (95% UI: 66,360.11, 139,791.43) and 380,293.98 prevalent strokes (95% UI: 273,753.18, 530,164.66). Western Sub-Saharan Africa had the largest increase number of incident cases and prevalent strokes. Sub-Saharan Africa (Eastern Sub-Saharan Africa, Southern Sub-Saharan Africa and Western Sub-Saharan Africa, Central Sub-Saharan Africa), Caribbean and Tropical Latin America suffered the severe threat with high age-standardized incidence rate, which of them, Eastern Sub-Saharan Africa had an increase by 0.04% (95% CI: 0.02, 0.07) from 28.68 to 100,000 population in 1990 to 28.89 per 100,000 in 2019 (Table 1). Sub-Saharan Africa (Eastern Sub-Saharan Africa, Southern Sub-Saharan Africa and Western Sub-Saharan Africa) also suffered the severe threat with high age-standardized prevalence rate, which of them, Eastern Sub-Saharan Africa had an increase by 0.37% (95% CI: 0.19, 0.56) from 134.84 to 100,000 population in 1990 to 148.43 per 100,000 in 2019 (Table S1). Meanwhile, High-income North America had the largest increase in ASIR (AAPC = 0.63; 95% CI: 0.59, 0.66) and ASPR (AAPC = 1.58; 95% CI: 0.54, 2.63) across 21 GBD regions (Table S1 and Table 1).

Although absolute numbers of deaths and DALYs decreased over the study period in other 19 GBD regions, it increased in Oceania and Western Sub-Saharan Africa. Oceania had the largest increase in absolute numbers of deaths (73.24%) and DALYs (73.44%), besides, it had the largest age-standardized mortality (3.32 per 100,000 population) and DALY rate (301.66 per 100,000 population) in 2019 across 21 GBD regions. Western Sub-Saharan Africa had the largest absolute numbers of deaths (4,535.32) and DALYs (420,873.60) in 2019. The largest decline in age-standardized mortality (AAPC= -7.96; 95% CI: -8.46, -7.45) and DALY rate (AAPC= -6.64; 95% CI: -7.04, -6.24) occurred in East Asia (Table S2 and Table 2).

### National trend in stroke prevalence, incidence, mortality and DALY rate

**Fig. 1:**
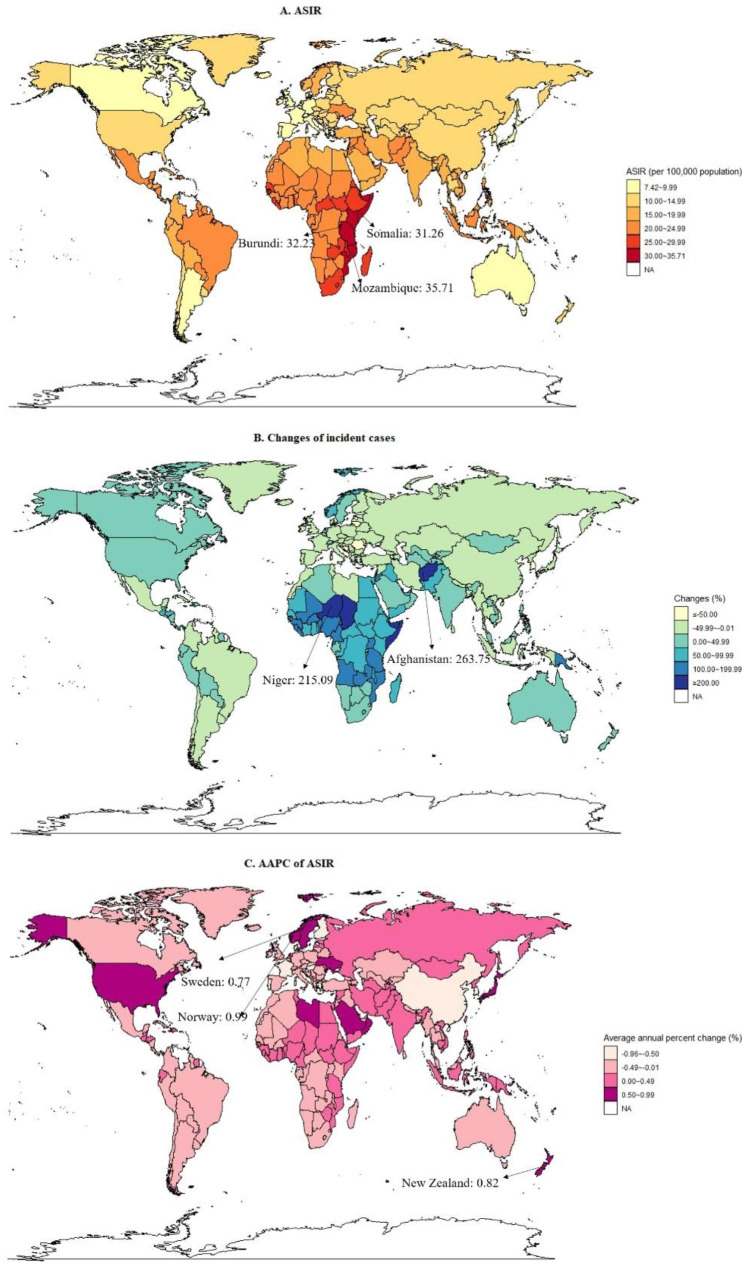
Global trends in stroke incidence among children (0–14 years) in 204 countries and territories. (**A**) age-standardized incidence rates in 2019; (**B**) changes in incident strokes between 1990 and 2019; (**C**) AAPCs of age-standardized incidence rates from 1990 to 2019. ASIR, age-standardized incidence rate; AAPC, average annual percentage change

**Fig. 2:**
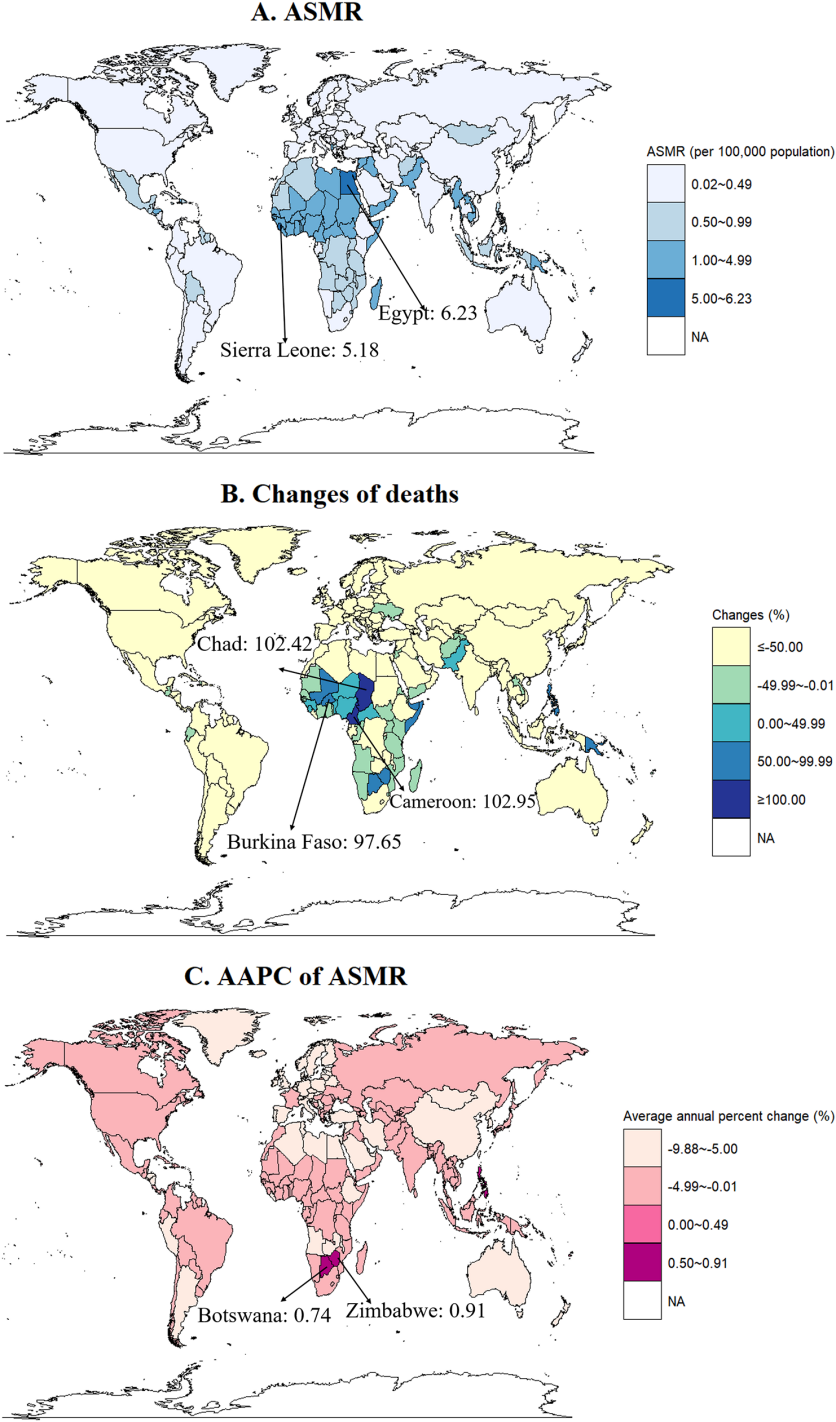
Global trends in stroke mortality among children (0–14 years) in 204 countries and territories. (**A**) age-standardized mortality rates in 2019; (**B**) changes in deaths from strokes between 1990 and 2019; (**C**) AAPCs of age-standardized mortality rates from 1990 to 2019. ASMR, age-standardized mortality rate; AAPC, average annual percentage change

At the country level in 2019, the ASIR for stroke was highest in Mozambique (35.71 per 100,000 population), followed by Burundi (32.23 per 100,000 population) and Somalia (31.26 per 100,000 population) (Fig. 1 and Table S3). The ASPR of stroke varied significantly across the 204 countries and territories. In 2019, the largest ASPR was in Mozambique (ASPR, 183.37 per 100,000 population), followed by Kenya (ASPR, 172.61 per 100,000) and Malawi (ASPR, 171.45 per 100,000) (Table S4). In 2019, across all the countries or territories, ASMR was highest in the Egypt (ASMR, 6.23 per 100,000 population), followed by Sierra Leone and Haiti (Fig. 2 and Table S5). Over the study period, Zimbabwe (AAPC = 0.91; 95% CI: 0.44, 1.37) and Botswana (AAPC = 0.74; 95% CI: 0.02, 1.47) were the only two countries with increasing trend of ASMR across 204 countries or territories (Fig. 2 and Table S5). Similarly, Egypt had the highest number of DALYs and ASDR across all the countries or territories in 2019 (Table S6). The detailed results of national trends in stroke incidence, prevalence, mortality and DALY rate are shown in the Table S3-S6.

### Influence factors associated with AAPC

**Fig. 3:**
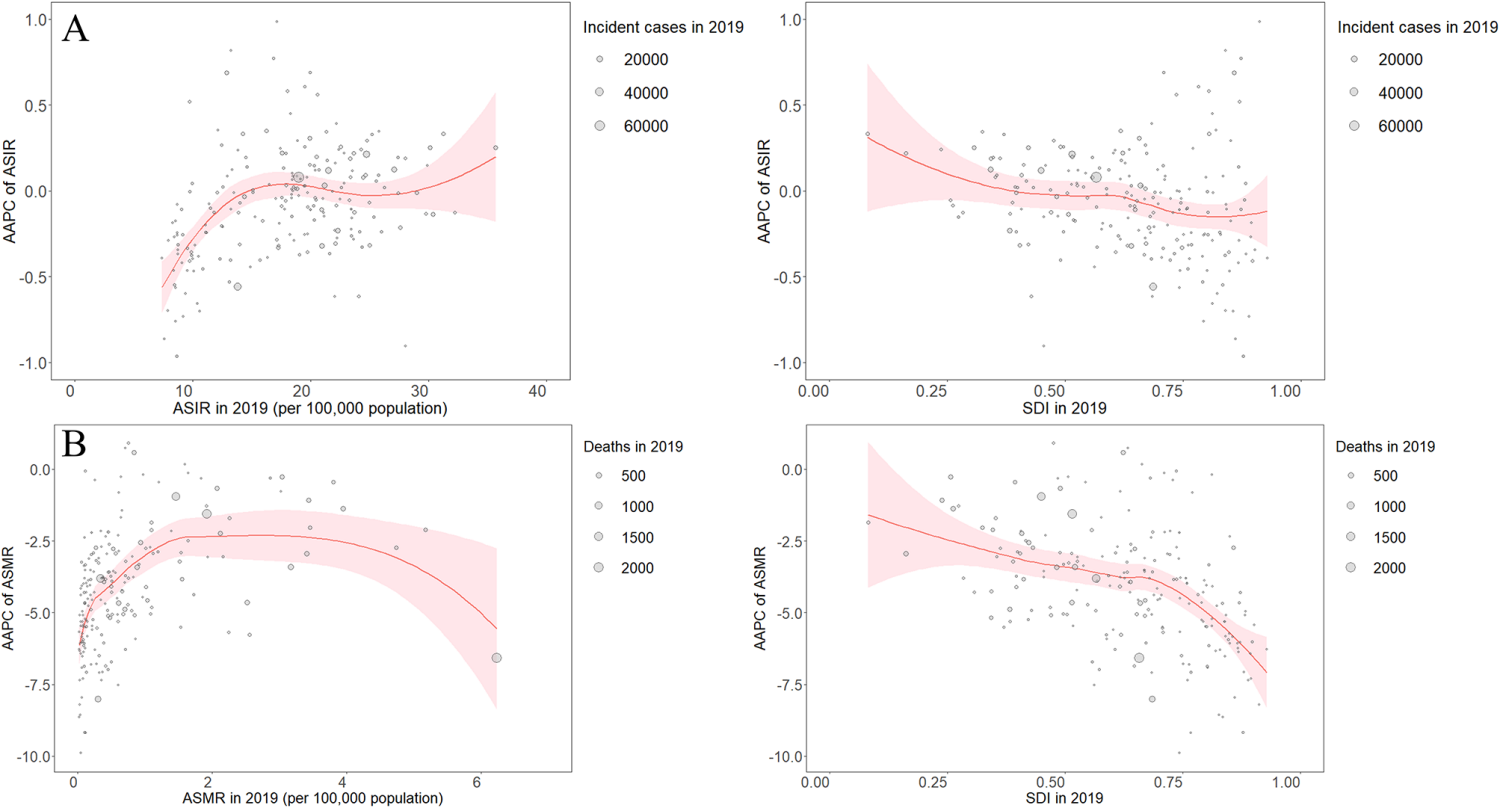
Average annual percentage changes of age-standardized incidence rates and age-standardized mortality rates of stroke at the country and territorial levels. (**A**) Correlation of average annual percentage changes with age-standardized incidence rates and sociodemographic indexes in 2019. (**B**) Correlation of average annual percentage changes with age-standardized mortality rates and sociodemographic indexes in 2019. The incident strokes and deaths from stroke from 204 countries and territories are represented by the circles. The circle size reflects the number of incident strokes and deaths from stroke. AAPC, average annual percentage change; ASIR, age-standardized incidence rate; ASMR, age-standardized mortality rate; SDI, sociodemographic index

A positive correlation was observed between AAPC of ASIR and ASIR in 2019 (ρ = 0.35, P < 0.001). In 2019, a significant negative correlation was detected between AAPC of ASIR and SDI (ρ= -0.21, P = 0.003) (Fig. 3A). Moreover, a significant positive correlation was observed between AAPC of ASMR and ASMR (ρ = 0.40, P < 0.001), and a significant negative correlation was found between AAPC of ASMR and SDI (ρ= -0.45, P < 0.001) (Fig. 3B). There was no correlation between AAPC of ASPR/ASDR and ASPR/ASDR or SDI (Figure S1).

## Discussion

This study used data from 2019 GBD study and systematically evaluated global trends and regional differences in stroke prevalence, incidence, mortality and DALY rate as well as the association with SDI at the national level among children aged less than 14 years. Findings of this study provided the comprehensive distribution and trend of stroke burden in children. We found that global incident strokes, ASIR, prevalent strokes and ASPR all increased, while deaths, ASMR, DALYs and ASDR all decreased over the past thirty years. In 2019, the highest age standardized incidence, prevalence, mortality, and DALY rates all occurred in low SDI regions. The greatest increase of age standardized incidence and prevalence both were in high SDI regions. Eastern Sub-Saharan Africa had the highest ASIR and ASPR in 2019, and Oceania had the highest ASMR and ASDR in 2019 across 21 GBD regions. High-income North America had the largest increase in ASIR and ASPR across 21 GBD regions. Against the overall decreasing trend of ASMR and ASDR, an increasing trend of them was found in Zimbabwe and Botswana. At the national level, we found a positive association of AAPC for ASIR or ASMR with ASIR or ASMR and a negative association of AAPC for ASIR or ASMR with SDI.

Our study provided more detail results and converse findings for children. GBD 2019 study reported that in general population, from 1990 to 2019, the global number of incident strokes and prevalent strokes both increased by over 70%, which was similar with our findings [1]. Our study also found that global incident strokes increased by 18.51%, and prevalent strokes increased by 31.97% among children. However, in contrast to the decreasing trend of stroke incidence and prevalence among general population, during the same period, we found that ASIR and ASPR both increased by 0.21% and 0.66% per year, respectively [1]. To be specific, high SDI regions had the greatest increase of ASIR and ASPR, and High-income North America had the largest increase across 21 GBD regions. Sundelin et al. also reported that the increasing number of childhood ischemic stroke from 1969 to 2016, and the highest number of childhood ischemic stroke reaching 1019 from 1997 to 2016 in Sweden [6]. Developed countries seem to present a greater burden of childhood stroke in the past decade [22]. Our findings revealed that the future clinical management and public health policy formulation should focus on this disease burden in childhood, not just for adults and older people. In fact, risk factors of stroke mainly were in adulthood such as atherosclerosis have been confirmed they began to present in childhood [23]. Multifactorial background of stroke occurrence in children is different from adults. Genetic risk factors have more clinical relevance, compared with non-genetic risk factors—environmental factors in children [10, 11]. In order to address this important global public health concern, continued efforts on screening the trend of stroke diseases burden and its modifiable risk factors, studying cutting-edge clinical assessment methods and techniques with higher sensitivity and specificity, and developing education programs of diagnosis and management in multidisciplinary medical personnel including cardiologists, hematologists, and cardiac intensivists, etc., were critical. Our study provided the updated and basic information of epidemiological distribution and pattern in this field.

In the general population, deaths due to stroke and DALYs due to stroke both increased, while mortality and DALY rate both decreased [1]. Similar results about trend of morality and DALY rate were found in children. In addition, we also found that the global number of DALYs and deaths decreased from 1990 to 2019. Although age-specific technical guidelines or recommendations for childhood stroke patients are unclear, the related technical development and drug therapy have reduced the risk of death from stroke [24–28]. For example, endovascular therapy was proposed to use for acute stroke among at least some children by experienced operators in an increasing number of reports [29]. This decline in childhood stroke morality and DALY may reflect improving recognition and poststroke medical management at global level. However, children in some regions or countries still faced a higher risk of death due to stroke. An increasing trend of ASMR and ASDR was found in Zimbabwe and Botswana. Additionally, in 2019, the number of stroke-related deaths and DALYs, ASMR and ASDR all were highest in low SDI region. Liesl Zühlke et al. found that patients from low- and lower-middle–income countries had significantly higher age- and sex-adjusted mortality than patients from upper-middle–income countries [30]. A large, multinational, prospective cohort reported that low income was associated with worse neurologic outcomes compared to higher income levels among children with arterial ischemic stroke [31]. Diagnosis, therapeutic interventions and management of stroke was relatively behindhand in low-income countries, international collaboration is desired to help in capacity building and research [32]. Higher mortality and DALY rate may reflect a poorer access to healthcare, limited availability of advanced treatment, and lower rates of hospitalization for stroke in developing regions [33]. Despite the decreased mortality and DALY rate, the regional gap should be paid attention.

There still had several limitations in this study. Firstly, the most notable limitation of this analysis was that the accuracy and robustness of GBD estimates largely depend on the quality and quantity of data used in the modelling [16]. For countries where national systematic surveillance and population-based studies were lacking or insufficient, the estimates might be a margin of bias. Secondly, due to GBD only provided data of population aged 14 years and over, not more specific age group, we could not analysis this population in detail.

## Conclusion

In conclusion, the overall increasing stroke incidence and prevalence indicated that prevention and management of stroke among younger population should be critical in the future. Despite falling trend in stroke mortality worldwide, specific countries or territories present worrying increase in stroke mortality. Without urgent implementation of effective primary prevention strategies, the stroke burden of children will probably continue to grow across the world, particularly in high-SDI countries.

### Electronic supplementary material

Below is the link to the electronic supplementary material.

Supplementary Material 1

## Data Availability

The datasets generated during and/or analyzed during the current study are available in the GBD repository, [http://ghdx.healthdata.org/gbd-results-tool].
