# Supplementary material for: Global trends and regional differences in disease burden of stroke among children: a trend analysis based on the global burden of disease study 2019
Source: BMC Public Health. 2023 Oct 27;23:2120. doi: 10.1186/s12889-023-17046-z (PMC10612321; doi:10.1186/s12889-023-17046-z)
Supplement: Supplementary file 1 — Supplementary Material 1 [file 12889_2023_17046_MOESM1_ESM.docx]

**Supplementary appendix**

This appendix formed part of the original submission.

[Table S1 1](#_Toc122616349)

[Table S2 1](#_Toc122616350)

[Table S3 4](#_Toc122616351)

[Table S4 10](#_Toc122616352)

[Table S5 16](#_Toc122616353)

[Table S6 22](#_Toc122616354)

[Figure S1 28](#_Toc122616356)

Table S1**.** **The prevalent strokes and age-standardized prevalence rate for stroke and their temporal change among children (0-14 years), 1990−2019**

|  | Prevalent strokes (95% UI) | | | Age-standardized prevalence (95% UI), per 100,000 | | |
| --- | --- | --- | --- | --- | --- | --- |
| **Characteristics** | 1990 | 2019 | Percentage change (%) | 1990 | 2019 | AAPC (95% CI) |
| **Global** | 1208128.16 (897742.74, 1606251.52) | 1594311.22 (1205044.34, 2110296.06) | 31.97% | 68.88 (51.18, 91.58) | 81.35 (61.49, 107.68) | 0.66 (0.36, 0.96) |
| **Sex** |  |  |  |  |  |  |
| Female | 749479.94 (998118.28, 561982.49) | 1010571.23 (1338841.67, 768797.20) | 34.84% | 116.97 (13.04, 65.86) | 141.22 (15.34, 81.09) | 0.76 (0.45, 1.06) |
| Male | 458648.22 (619163.39, 335767.26) | 583739.99 (776259.84, 431559.78) | 27.27% | 68.74 (8.03, 37.28) | 76.73 (8.69, 42.66) | 0.51 (0.22, 0.80) |
| **Socio-demographic index** |  |  |  |  |  |  |
| Low SDI | 222763.29 (175379.52, 288442.57) | 499007.79 (389763.73, 643897.74) | 124.01% | 92.04 (72.47, 119.18) | 105.53 (82.42, 136.17) | 0.53 (0.29, 0.77) |
| Low-middle SDI | 319981.72 (237252.44, 425569.03) | 436718.18 (326309.17, 581541.52) | 36.48% | 70.57 (52.33, 93.86) | 83.42 (62.33, 111.08) | 0.68 (0.34, 1.02) |
| Middle SDI | 406135.55 (297834.57, 548744.84) | 410153.49 (300733.37, 548874.72) | 0.99% | 69.86 (51.23, 94.39) | 74.09 (54.32, 99.15) | 0.26 (0.04, 0.48) |
| High-middle SDI | 172276.97 (123251.42, 235585.95) | 139033.29 (101686.07, 190254.96) | -19.30% | 56.76 (40.60, 77.61) | 56.70 (41.47, 77.59) | 0.02 (-0.14, 0.18) |
| High SDI | 86209.10 (62482.04, 115242.68) | 108366.01 (77869.81, 146430.82) | 25.70% | 49.93 (36.18, 66.74) | 66.38 (47.70, 89.69) | 1.15 (0.34, 1.96) |
| **GBD regions** |  |  |  |  |  |  |
| Andean Latin America | 9727.87 (7356.13, 12546.87) | 12307.86 (9445.06, 15737.91) | 26.52% | 64.66 (48.90, 83.40) | 68.03 (52.21, 86.99) | 0.23 (0.00, 0.46) |
| Australasia | 1739.13 (1123.03, 2418.89) | 2105.56 (1385.57, 2982.09) | 21.07% | 37.91 (24.48, 52.73) | 38.40 (25.27, 54.38) | 0.06 (-0.36, 0.48) |
| Caribbean | 10964.18 (8468.27, 13796.24) | 11628.56 (9084.71, 14673.51) | 6.06% | 96.06 (74.19, 120.88) | 99.53 (77.76, 125.59) | 0.18 (-0.07, 0.43) |
| Central Asia | 7271.49 (5305.49, 9705.01) | 8390.50 (5781.42, 11464.18) | 15.39% | 29.14 (21.26, 38.89) | 31.19 (21.49, 42.62) | 0.29 (-0.05, 0.62) |
| Central Europe | 13862.33 (9836.38, 18771.23) | 8045.65 (5783.30, 10818.52) | -41.96% | 47.89 (33.98, 64.85) | 45.62 (32.79, 61.34) | 0.00 (-0.42, 0.43) |
| Central Latin America | 59860.55 (45329.73, 78915.06) | 62999.19 (47573.10, 82179.86) | 5.24% | 93.39 (70.72, 123.12) | 96.35 (72.75, 125.68) | 0.14 (-0.07, 0.36) |
| Central Sub-Saharan Africa | 24912.98 (19337.63, 31379.59) | 56926.71 (43457.22, 73412.64) | 128.50% | 96.44 (74.86, 121.47) | 99.78 (76.17, 128.68) | 0.15 (-0.11, 0.42) |
| East Asia | 202609.14 (139344.32, 285540.15) | 114608.42 (77809.31, 163973.21) | -43.43% | 60.49 (41.60, 85.25) | 49.25 (33.44, 70.46) | -0.62 (-0.89, -0.35) |
| Eastern Europe | 22092.35 (15223.43, 30671.64) | 22130.96 (15798.09, 30304.87) | 0.17% | 42.94 (29.59, 59.61) | 59.72 (42.63, 81.77) | 1.19 (0.85, 1.54) |
| Eastern Sub-Saharan Africa | 121851.48 (96144.19, 156341.49) | 262024.41 (205155.30, 334244.29) | 115.04% | 134.84 (106.39, 173.01) | 148.43 (116.22, 189.34) | 0.37 (0.19, 0.56) |
| High-income Asia Pacific | 9534.86 (6533.29, 13215.76) | 8142.29 (5476.34, 11586.68) | -14.61% | 27.05 (18.54, 37.50) | 34.88 (23.46, 49.64) | 0.94 (0.50, 1.38) |
| High-income North America | 37063.96 (25359.22, 52033.48) | 59508.09 (41430.59, 82918.81) | 60.56% | 60.35 (41.29, 84.73) | 89.80 (62.52, 125.12) | 1.58 (0.54, 2.63) |
| North Africa and Middle East | 107397.72 (83229.30, 136864.28) | 146743.23 (115108.22, 185176.14) | 36.64% | 74.73 (57.91, 95.23) | 83.45 (65.46, 105.31) | 0.43 (0.22, 0.64) |
| Oceania | 1313.76 (963.96, 1733.60) | 2281.02 (1650.31, 3041.07) | 73.63% | 50.00 (36.68, 65.97) | 47.23 (34.17, 62.96) | -0.17 (-0.29, -0.05) |
| South Asia | 274821.37 (197655.92, 376695.52) | 380293.98 (273753.18, 530164.66) | 38.38% | 62.60 (45.03, 85.81) | 73.59 (52.97, 102.59) | 0.69 (0.38, 1.00) |
| Southeast Asia | 101310.24 (74837.12, 135808.41) | 109368.06 (81009.41, 145638.41) | 7.95% | 58.82 (43.45, 78.85) | 64.80 (48.00, 86.29) | 0.38 (0.18, 0.58) |
| Southern Latin America | 3955.69 (2720.55, 5398.68) | 3952.12 (2610.38, 5654.21) | -0.09% | 26.50 (18.22, 36.16) | 26.51 (17.51, 37.93) | 0.02 (-0.15, 0.19) |
| Southern Sub-Saharan Africa | 29918.31 (22506.36, 39937.14) | 33579.55 (25587.16, 44595.63) | 12.24% | 146.84 (110.46, 196.01) | 142.20 (108.35, 188.85) | -0.09 (-0.13, -0.05) |
| Tropical Latin America | 54564.26 (39996.93, 74946.36) | 45775.64 (34309.74, 61626.00) | -16.11% | 101.15 (74.14, 138.93) | 92.08 (69.02, 123.96) | -0.32 (-0.47, -0.17) |
| Western Europe | 28188.83 (20072.84, 38143.55) | 28462.44 (20398.67, 38275.17) | 0.97% | 39.67 (28.25, 53.68) | 41.36 (29.64, 55.61) | 0.23 (-0.22, 0.68) |
| Western Sub-Saharan Africa | 85167.65 (66334.75, 111700.67) | 215036.99 (166471.07, 281423.05) | 152.49% | 96.98 (75.54, 127.20) | 108.42 (83.94, 141.90) | 0.43 (0.24, 0.61) |

Note: AAPC, average annual percentage change; CI, confidence interval; GBD, Global Burden of Disease; SDI, socio-demographic index; UI, uncertainty interval

Table S2**. The DALYs and age-standardized DALY rate due to stroke and their temporal change among children (0-14 years), 1990−2019**

|  | DALYs (95% UI) | |  | Age-standardized DALY rate (95% UI), per 100,000 population | | |
| --- | --- | --- | --- | --- | --- | --- |
| **Characteristics** | 1990 | 2019 | Percentage change (%) | 1990 | 2019 | AAPC (95% CI) |
| **Global** | 4215300.72 (3279611.22, 5551082.27) | 1678539.47 (1390409.59, 2034897.27) | -60.18% | 240.33 (186.98, 316.49) | 85.65 (70.95, 103.84) | -3.50 (-3.64, -3.36) |
| **Sex** |  |  |  |  |  |  |
| Female | 1891813.98 (2548723.53, 1475723.59) | 777362.05 (939880.33, 644559.55) | -58.91% | 221.71 (172.95, 298.70) | 82.00 (67.99, 99.14) | -3.40 (-3.59, -3.21) |
| Male | 2323486.74 (3079091.10, 1701450.68) | 901177.43 (1136531.76, 734500.49) | -61.21% | 257.97 (188.91, 341.86) | 89.08 (72.60, 112.34) | -3.63 (-3.74, -3.52) |
| **Socio-demographic index** | | | | | | |
| Low SDI | 603405.55 (429182.28, 878574.60) | 628505.73 (496162.08, 828266.90) | 4.16% | 249.32 (177.34, 363.02) | 132.91 (104.92, 175.16) | -2.14 (-2.28, -1.99) |
| Low-middle SDI | 943546.30 (695977.31, 1356562.76) | 447056.27 (370755.74, 529510.43) | -52.62% | 208.11 (153.50, 299.20) | 85.39 (70.82, 101.14) | -3.03 (-3.13, -2.94) |
| Middle SDI | 2106607.48 (1430783.16, 2705378.47) | 467449.30 (365979.57, 596855.08) | -77.81% | 362.36 (246.11, 465.36) | 84.44 (66.11, 107.81) | -4.89 (-5.25, -4.54) |
| High-middle SDI | 452110.68 (352891.60, 553174.06) | 87906.54 (72265.90, 106422.52) | -80.56% | 148.95 (116.26, 182.24) | 35.85 (29.47, 43.40) | -4.83 (-5.09, -4.57) |
| High SDI | 106381.55 (96477.80, 118235.07) | 45537.07 (36614.90, 55742.48) | -57.19% | 61.61 (55.87, 68.47) | 27.89 (22.43, 34.14) | -2.63 (-2.91, -2.35) |
| **GBD region** |  |  |  |  |  |  |
| Andean Latin America | 35956.36 (26768.40, 44539.31) | 9176.93 (7337.49, 11546.41) | -74.48% | 239.00 (177.93, 296.05) | 50.73 (40.56, 63.82) | -5.23 (-5.45, -5.01) |
| Australasia | 1451.71 (1219.54, 1718.87) | 798.69 (595.55, 1032.38) | -44.98% | 31.65 (26.59, 37.47) | 14.57 (10.86, 18.83) | -2.61 (-3.15, -2.07) |
| Caribbean | 36544.19 (22558.03, 52452.49) | 22031.14 (13748.98, 33376.23) | -39.71% | 320.18 (197.64, 459.56) | 188.56 (117.68, 285.67) | -1.81 (-1.98, -1.64) |
| Central Asia | 12860.11 (10228.46, 15118.30) | 6536.82 (5191.81, 8150.06) | -49.17% | 51.53 (40.99, 60.58) | 24.30 (19.30, 30.30) | -2.55 (-2.81, -2.30) |
| Central Europe | 24450.90 (21210.74, 27822.16) | 5801.90 (4668.47, 7094.11) | -76.27% | 84.47 (73.28, 96.12) | 32.90 (26.47, 40.22) | -3.21 (-3.46, -2.95) |
| Central Latin America | 82446.65 (73337.42, 90078.70) | 34837.86 (26377.47, 46977.54) | -57.74% | 128.63 (114.42, 140.53) | 53.28 (40.34, 71.84) | -2.94 (-3.08, -2.80) |
| Central Sub-Saharan Africa | 70930.04 (39178.16, 113887.66) | 46350.90 (33154.90, 65451.70) | -34.65% | 274.57 (151.66, 440.86) | 81.25 (58.11, 114.73) | -4.10 (-4.25, -3.95) |
| East Asia | 1027121.35 (712402.50, 1257884.50) | 97491.18 (76970.96, 125404.54) | -90.51% | 306.66 (212.70, 375.56) | 41.89 (33.08, 53.89) | -6.64 (-7.04, -6.24) |
| Eastern Europe | 17602.53 (14106.23, 21304.34) | 10037.07 (7525.05, 13159.54) | -42.98% | 34.21 (27.42, 41.41) | 27.08 (20.31, 35.51) | -0.80 (-1.40, -0.21) |
| Eastern Sub-Saharan Africa | 173475.77 (114718.04, 243813.76) | 133327.94 (97444.65, 183061.82) | -23.14% | 191.97 (126.95, 269.80) | 75.53 (55.20, 103.70) | -3.17 (-3.29, -3.04) |
| High-income Asia Pacific | 28885.54 (24897.75, 33318.45) | 4953.00 (3849.21, 6236.82) | -82.85% | 81.96 (70.65, 94.54) | 21.22 (16.49, 26.72) | -4.57 (-4.83, -4.30) |
| High-income North America | 38322.89 (34666.54, 43119.06) | 25819.47 (21223.95, 31412.51) | -32.63% | 62.40 (56.45, 70.21) | 38.96 (32.03, 47.40) | -1.60 (-2.14, -1.06) |
| North Africa and Middle East | 1282714.07 (737206.06, 1802158.65) | 313314.56 (219773.08, 442756.94) | -75.57% | 892.49 (512.94, 1253.92) | 178.18 (124.98, 251.79) | -5.41 (-5.65, -5.18) |
| Oceania | 8400.77 (4776.70, 13505.71) | 14570.24 (8386.35, 23391.82) | 73.44% | 319.70 (181.78, 513.98) | 301.66 (173.63, 484.31) | -0.22 (-0.49, 0.05) |
| South Asia | 537604.25 (405324.84, 710886.58) | 323542.80 (265822.29, 384174.47) | -39.82% | 122.47 (92.33, 161.94) | 62.61 (51.44, 74.34) | -2.34 (-2.46, -2.21) |
| Southeast Asia | 401290.35 (264056.03, 630517.07) | 158866.77 (126359.25, 193070.29) | -60.41% | 233.00 (153.32, 366.09) | 94.13 (74.87, 114.40) | -3.08 (-3.25, -2.90) |
| Southern Latin America | 15698.25 (13498.45, 18217.28) | 4319.71 (3577.44, 5108.51) | -72.48% | 105.15 (90.41, 122.02) | 28.98 (24.00, 34.27) | -4.42 (-4.57, -4.28) |
| Southern Sub-Saharan Africa | 18971.15 (12437.56, 25886.39) | 12838.13 (9878.83, 16448.83) | -32.33% | 93.11 (61.04, 127.05) | 54.37 (41.83, 69.66) | -1.78 (-2.10, -1.45) |
| Tropical Latin America | 63073.26 (52029.15, 76902.27) | 20952.04 (17287.26, 24926.86) | -66.78% | 116.92 (96.45, 142.56) | 42.15 (34.77, 50.14) | -3.42 (-3.67, -3.17) |
| Western Europe | 38461.90 (34743.77, 43268.26) | 12098.73 (9577.61, 15077.01) | -68.54% | 54.13 (48.90, 60.90) | 17.58 (13.92, 21.91) | -3.82 (-4.03, -3.61) |
| Western Sub-Saharan Africa | 299038.68 (216927.76, 435585.27) | 420873.60 (316057.35, 610893.61) | 40.74% | 340.52 (247.02, 496.01) | 212.21 (159.36, 308.02) | -1.60 (-1.80, -1.40) |

Note: ASR: age-standardized rates; AAPC, average annual percentage change; CI, confidence interval; disability-adjusted life-years: DALYs; UI, uncertainty interval

Table S3**. The incident strokes and age-standardized incidence rate for stroke and their temporal change among children (0-14 years) in 204 countries or territories from 1990 to 2019.**

|  | Incident strokes (95% UI) | | | Age-standardized incidence rate (95% UI), per 100,000 population | | |
| --- | --- | --- | --- | --- | --- | --- |
| **Countries or territories** | 1990 | 2019 | Percentage change (%) | 1990 | 2019 | AAPC (95% CI) |
| Afghanistan | 1108.35 (771.51, 1524.51) | 4031.58 (2798.66, 5564.88) | 263.75% | 22.21 (15.46, 30.55) | 23.36 (16.22, 32.24) | 0.19 (0.10, 0.28) |
| Albania | 105.23 (68.81, 156.08) | 41.22 (27.02, 62.04) | -60.83% | 9.36 (6.12, 13.89) | 8.72 (5.71, 13.12) | -0.24 (-0.26, -0.22) |
| Algeria | 1953.63 (1344.98, 2782.39) | 2103.87 (1438.65, 2985.63) | 7.69% | 18.22 (12.55, 25.95) | 17.92 (12.26, 25.44) | -0.06 (-0.10, -0.02) |
| American Samoa | 3.48 (2.41, 4.84) | 3.13 (2.19, 4.34) | -10.07% | 18.39 (12.73, 25.59) | 18.83 (13.18, 26.17) | 0.08 (0.06, 0.10) |
| Andorra | 0.87 (0.55, 1.33) | 0.91 (0.57, 1.43) | 3.96% | 9.18 (5.76, 14.03) | 8.47 (5.32, 13.39) | -0.27 (-0.31, -0.23) |
| Angola | 1170.93 (815.91, 1620.04) | 3315.26 (2264.29, 4705.37) | 183.13% | 24.66 (17.18, 34.12) | 23.70 (16.19, 33.64) | -0.12 (-0.17, -0.08) |
| Antigua and Barbuda | 3.87 (2.67, 5.39) | 3.38 (2.31, 4.75) | -12.65% | 20.67 (14.30, 28.84) | 20.00 (13.66, 28.12) | -0.11 (-0.13, -0.09) |
| Argentina | 1067.94 (706.62, 1593.06) | 1011.73 (657.30, 1512.33) | -5.26% | 10.54 (6.97, 15.72) | 9.57 (6.22, 14.30) | -0.33 (-0.35, -0.32) |
| Armenia | 139.36 (93.35, 201.42) | 76.43 (50.75, 111.63) | -45.16% | 13.38 (8.96, 19.34) | 12.56 (8.34, 18.34) | -0.21 (-0.31, -0.11) |
| Australia | 419.93 (268.66, 631.70) | 454.82 (292.53, 691.62) | 8.31% | 11.09 (7.09, 16.68) | 9.91 (6.37, 15.06) | -0.38 (-0.42, -0.33) |
| Austria | 156.15 (101.27, 231.11) | 130.07 (85.11, 197.10) | -16.70% | 11.58 (7.51, 17.13) | 10.09 (6.60, 15.29) | -0.47 (-0.49, -0.44) |
| Azerbaijan | 336.11 (224.13, 483.60) | 285.73 (189.03, 417.80) | -14.99% | 13.83 (9.23, 19.91) | 12.28 (8.12, 17.96) | -0.40 (-0.44, -0.35) |
| Bahamas | 17.14 (11.80, 23.81) | 15.80 (10.81, 22.07) | -7.86% | 21.32 (14.68, 29.61) | 19.77 (13.53, 27.62) | -0.25 (-0.28, -0.22) |
| Bahrain | 32.20 (22.35, 44.87) | 42.83 (29.05, 60.87) | 32.99% | 19.63 (13.63, 27.36) | 18.24 (12.37, 25.92) | -0.26 (-0.31, -0.20) |
| Bangladesh | 7049.54 (4565.33, 10314.60) | 6336.77 (4109.39, 9317.74) | -10.11% | 14.55 (9.43, 21.29) | 14.39 (9.33, 21.15) | -0.03 (-0.08, 0.01) |
| Barbados | 13.78 (9.45, 19.42) | 10.80 (7.47, 15.03) | -21.65% | 21.96 (15.06, 30.94) | 21.86 (15.13, 30.43) | -0.01 (-0.13, 0.13) |
| Belarus | 345.45 (238.68, 487.31) | 230.41 (156.19, 326.84) | -33.30% | 14.32 (9.89, 20.20) | 14.01 (9.50, 19.88) | -0.07 (-0.12, -0.01) |
| Belgium | 162.53 (103.06, 250.46) | 165.11 (101.63, 255.86) | 1.58% | 9.00 (5.70, 13.86) | 8.54 (5.26, 13.24) | -0.17 (-0.19, -0.16) |
| Belize | 17.40 (11.91, 24.54) | 28.01 (19.32, 39.47) | 60.97% | 21.54 (14.74, 30.38) | 22.86 (15.77, 32.22) | 0.22 (0.16, 0.28) |
| Benin | 554.79 (377.74, 785.29) | 1349.29 (909.45, 1912.39) | 143.21% | 22.88 (15.58, 32.39) | 23.42 (15.78, 33.19) | 0.08 (0.06, 0.10) |
| Bermuda | 2.42 (1.68, 3.38) | 1.64 (1.12, 2.33) | -32.28% | 20.27 (14.06, 28.29) | 18.59 (12.70, 26.39) | -0.29 (-0.31, -0.27) |
| Bhutan | 40.44 (26.79, 59.35) | 29.77 (19.43, 43.03) | -26.39% | 15.97 (10.58, 23.44) | 15.30 (9.98, 22.11) | -0.15 (-0.18, -0.12) |
| Bolivia (Plurinational State of) | 553.83 (388.33, 769.47) | 774.79 (535.25, 1079.64) | 39.90% | 20.29 (14.22, 28.18) | 19.73 (13.63, 27.50) | -0.09 (-0.12, -0.07) |
| Bosnia and Herzegovina | 130.14 (86.08, 190.17) | 52.95 (35.76, 79.24) | -59.31% | 11.78 (7.79, 17.21) | 11.11 (7.50, 16.63) | -0.20 (-0.22, -0.18) |
| Botswana | 140.72 (96.69, 196.62) | 165.57 (114.20, 230.14) | 17.66% | 24.63 (16.92, 34.41) | 23.48 (16.19, 32.63) | -0.16 (-0.18, -0.14) |
| Brazil | 12118.99 (8461.34, 16959.69) | 10014.12 (6922.91, 14069.73) | -17.37% | 23.18 (16.19, 32.45) | 20.97 (14.50, 29.47) | -0.32 (-0.44, -0.20) |
| Brunei Darussalam | 10.13 (6.61, 14.85) | 8.95 (5.62, 13.59) | -11.60% | 11.17 (7.29, 16.37) | 9.41 (5.91, 14.29) | -0.60 (-0.63, -0.57) |
| Bulgaria | 195.13 (133.80, 286.67) | 99.07 (64.87, 149.20) | -49.23% | 11.24 (7.70, 16.51) | 10.01 (6.55, 15.07) | -0.40 (-0.43, -0.37) |
| Burkina Faso | 1119.51 (767.68, 1580.67) | 2420.34 (1662.14, 3426.18) | 116.20% | 23.68 (16.24, 33.43) | 23.29 (15.99, 32.96) | -0.06 (-0.09, -0.03) |
| Burundi | 886.77 (616.16, 1216.27) | 1740.85 (1194.92, 2460.98) | 96.31% | 33.60 (23.35, 46.09) | 32.23 (22.12, 45.56) | -0.13 (-0.19, -0.07) |
| Cabo Verde | 35.12 (23.91, 50.29) | 36.35 (24.69, 51.21) | 3.50% | 22.64 (15.41, 32.42) | 22.86 (15.53, 32.21) | 0.04 (0.00, 0.08) |
| Cambodia | 717.15 (486.02, 1014.67) | 758.84 (513.09, 1082.86) | 5.81% | 15.04 (10.19, 21.27) | 15.08 (10.19, 21.51) | 0.01 (-0.02, 0.03) |
| Cameroon | 1014.11 (687.39, 1471.18) | 2711.64 (1844.21, 3864.07) | 167.39% | 20.96 (14.21, 30.40) | 22.53 (15.32, 32.10) | 0.26 (0.20, 0.31) |
| Canada | 538.38 (334.52, 824.87) | 552.42 (341.15, 840.90) | 2.61% | 9.36 (5.81, 14.34) | 9.05 (5.59, 13.78) | -0.11 (-0.16, -0.05) |
| Central African Republic | 324.43 (222.12, 454.26) | 565.65 (386.84, 785.00) | 74.35% | 26.49 (18.14, 37.10) | 25.33 (17.32, 35.15) | -0.15 (-0.18, -0.13) |
| Chad | 663.41 (452.49, 942.11) | 2019.91 (1386.57, 2854.35) | 204.48% | 22.70 (15.48, 32.24) | 24.31 (16.69, 34.35) | 0.24 (0.21, 0.27) |
| Chile | 472.82 (322.86, 684.69) | 416.77 (281.67, 608.70) | -11.85% | 11.89 (8.12, 17.21) | 11.49 (7.76, 16.77) | -0.10 (-0.17, -0.04) |
| China | 52229.00 (33769.52, 76408.28) | 31024.69 (19759.15, 46630.14) | -40.60% | 16.17 (10.46, 23.66) | 13.80 (8.79, 20.74) | -0.56 (-0.63, -0.49) |
| Colombia | 2233.91 (1550.22, 3141.86) | 1906.36 (1293.30, 2740.13) | -14.66% | 19.08 (13.24, 26.83) | 17.29 (11.73, 24.85) | -0.32 (-0.38, -0.26) |
| Comoros | 77.37 (55.39, 104.51) | 65.24 (44.71, 90.31) | -15.67% | 36.42 (26.07, 49.19) | 28.03 (19.21, 38.79) | -0.91 (-1.00, -0.81) |
| Congo | 295.73 (205.18, 416.14) | 483.77 (331.48, 675.37) | 63.58% | 26.98 (18.72, 37.96) | 24.34 (16.68, 33.98) | -0.33 (-0.39, -0.26) |
| Cook Islands | 1.23 (0.85, 1.72) | 0.77 (0.54, 1.08) | -37.57% | 18.36 (12.69, 25.70) | 18.39 (12.82, 25.76) | 0.01 (-0.02, 0.04) |
| Costa Rica | 215.36 (149.23, 304.11) | 193.91 (131.52, 275.54) | -9.96% | 19.18 (13.29, 27.09) | 18.38 (12.47, 26.12) | -0.15 (-0.18, -0.12) |
| Croatia | 105.11 (68.92, 157.41) | 52.01 (33.60, 79.47) | -50.52% | 10.65 (6.98, 15.95) | 8.53 (5.51, 13.04) | -0.76 (-0.80, -0.72) |
| Cuba | 531.09 (364.93, 746.28) | 341.96 (231.69, 484.28) | -35.61% | 21.25 (14.60, 29.86) | 19.05 (12.90, 26.97) | -0.37 (-0.43, -0.31) |
| Cyprus | 17.64 (11.27, 27.07) | 18.52 (11.88, 28.57) | 5.01% | 8.91 (5.69, 13.67) | 8.69 (5.58, 13.41) | -0.08 (-0.12, -0.04) |
| Czechia | 261.55 (176.49, 377.21) | 189.02 (127.26, 275.57) | -27.73% | 11.90 (8.03, 17.16) | 11.09 (7.47, 16.17) | -0.24 (-0.27, -0.22) |
| Cote d'Ivoire | 1380.64 (947.43, 1963.05) | 2634.99 (1802.51, 3716.92) | 90.85% | 24.10 (16.54, 34.26) | 24.66 (16.87, 34.79) | 0.09 (0.01, 0.17) |
| Democratic People's Republic of Korea | 892.08 (599.93, 1282.57) | 709.97 (481.97, 1010.10) | -20.41% | 13.70 (9.21, 19.70) | 14.49 (9.84, 20.61) | 0.20 (0.18, 0.21) |
| Democratic Republic of the Congo | 4335.31 (2992.43, 6093.45) | 8401.36 (5700.01, 11875.66) | 93.79% | 23.90 (16.50, 33.59) | 22.29 (15.12, 31.50) | -0.23 (-0.29, -0.18) |
| Denmark | 100.65 (64.81, 151.60) | 88.53 (56.20, 135.32) | -12.04% | 11.40 (7.34, 17.16) | 9.22 (5.85, 14.09) | -0.73 (-0.75, -0.71) |
| Djibouti | 63.05 (44.07, 87.10) | 125.85 (87.17, 177.76) | 99.62% | 28.69 (20.05, 39.63) | 29.92 (20.73, 42.27) | 0.15 (0.10, 0.20) |
| Dominica | 5.39 (3.72, 7.42) | 3.06 (2.09, 4.30) | -43.31% | 21.97 (15.16, 30.22) | 21.10 (14.44, 29.65) | -0.13 (-0.18, -0.07) |
| Dominican Republic | 547.80 (368.94, 771.49) | 630.02 (426.65, 889.66) | 15.01% | 19.97 (13.45, 28.12) | 20.70 (14.02, 29.23) | 0.12 (0.06, 0.18) |
| Ecuador | 757.63 (526.96, 1060.23) | 980.69 (669.94, 1374.43) | 29.44% | 19.38 (13.48, 27.11) | 19.48 (13.31, 27.31) | 0.03 (-0.01, 0.07) |
| Egypt | 4091.97 (2756.80, 5744.97) | 6520.36 (4471.14, 9140.69) | 59.35% | 18.38 (12.39, 25.81) | 19.97 (13.69, 27.99) | 0.31 (0.21, 0.40) |
| El Salvador | 362.51 (248.68, 506.87) | 302.17 (205.21, 429.96) | -16.65% | 17.06 (11.70, 23.85) | 17.70 (12.02, 25.19) | 0.13 (0.11, 0.15) |
| Equatorial Guinea | 54.09 (37.36, 74.75) | 124.33 (85.48, 174.50) | 129.87% | 26.34 (18.19, 36.40) | 22.03 (15.14, 30.92) | -0.61 (-0.66, -0.57) |
| Eritrea | 394.11 (266.37, 555.94) | 686.97 (469.62, 980.83) | 74.31% | 27.98 (18.91, 39.47) | 26.01 (17.78, 37.14) | -0.24 (-0.31, -0.17) |
| Estonia | 43.86 (29.50, 62.66) | 27.91 (18.86, 40.33) | -36.36% | 12.56 (8.45, 17.94) | 12.95 (8.75, 18.71) | 0.09 (0.01, 0.16) |
| Eswatini | 96.52 (66.04, 135.54) | 95.94 (66.44, 134.99) | -0.60% | 25.05 (17.14, 35.17) | 23.44 (16.24, 32.99) | -0.22 (-0.26, -0.18) |
| Ethiopia | 6495.08 (4469.13, 9201.84) | 12478.63 (8275.33, 17775.68) | 92.12% | 26.22 (18.04, 37.14) | 27.09 (17.96, 38.59) | 0.12 (0.08, 0.17) |
| Fiji | 54.69 (38.16, 77.01) | 51.69 (36.09, 71.76) | -5.50% | 19.39 (13.53, 27.30) | 19.47 (13.59, 27.03) | 0.03 (-0.04, 0.10) |
| Finland | 126.21 (83.28, 192.06) | 92.59 (60.85, 139.71) | -26.64% | 13.07 (8.63, 19.89) | 10.59 (6.96, 15.98) | -0.70 (-0.81, -0.60) |
| France | 1158.71 (759.78, 1734.56) | 995.84 (652.17, 1505.84) | -14.06% | 9.90 (6.49, 14.81) | 8.43 (5.52, 12.75) | -0.55 (-0.64, -0.46) |
| Gabon | 99.03 (69.16, 138.37) | 128.44 (87.39, 181.09) | 29.69% | 23.64 (16.51, 33.03) | 22.05 (15.00, 31.08) | -0.23 (-0.26, -0.21) |
| Gambia | 123.24 (86.26, 172.24) | 242.96 (169.23, 337.89) | 97.14% | 26.17 (18.32, 36.58) | 26.27 (18.30, 36.53) | 0.02 (-0.02, 0.06) |
| Georgia | 185.02 (127.87, 259.58) | 97.85 (65.24, 143.41) | -47.11% | 13.54 (9.36, 18.99) | 13.49 (9.00, 19.78) | -0.01 (-0.03, 0.01) |
| Germany | 1418.46 (928.78, 2114.99) | 1129.26 (714.45, 1689.45) | -20.39% | 10.95 (7.17, 16.33) | 9.74 (6.16, 14.56) | -0.41 (-0.43, -0.39) |
| Ghana | 1616.55 (1108.78, 2251.88) | 2766.37 (1915.07, 3848.11) | 71.13% | 23.95 (16.42, 33.36) | 24.47 (16.94, 34.03) | 0.08 (0.06, 0.10) |
| Greece | 190.02 (122.48, 296.08) | 127.45 (79.66, 196.76) | -32.93% | 9.39 (6.06, 14.64) | 8.72 (5.45, 13.46) | -0.26 (-0.28, -0.24) |
| Greenland | 1.58 (1.00, 2.39) | 1.19 (0.74, 1.80) | -24.96% | 11.14 (7.04, 16.84) | 10.19 (6.34, 15.48) | -0.32 (-0.35, -0.28) |
| Grenada | 7.33 (5.09, 10.17) | 5.02 (3.46, 6.98) | -31.47% | 22.86 (15.89, 31.74) | 22.50 (15.50, 31.27) | -0.05 (-0.07, -0.03) |
| Guam | 6.70 (4.61, 9.43) | 8.32 (5.72, 11.70) | 24.24% | 16.05 (11.04, 22.60) | 18.28 (12.56, 25.71) | 0.45 (0.43, 0.47) |
| Guatemala | 648.63 (446.03, 911.21) | 1045.22 (705.09, 1490.79) | 61.14% | 17.78 (12.23, 24.98) | 17.93 (12.10, 25.58) | 0.03 (0.02, 0.05) |
| Guinea | 547.54 (363.44, 805.17) | 1218.63 (818.37, 1748.63) | 122.56% | 19.30 (12.81, 28.38) | 21.33 (14.32, 30.61) | 0.34 (0.31, 0.38) |
| Guinea-Bissau | 119.47 (81.78, 166.25) | 204.67 (141.75, 287.42) | 71.32% | 25.10 (17.18, 34.92) | 25.76 (17.84, 36.17) | 0.09 (0.08, 0.11) |
| Guyana | 73.42 (52.02, 100.68) | 49.32 (34.08, 67.64) | -32.83% | 25.80 (18.28, 35.37) | 23.63 (16.33, 32.42) | -0.28 (-0.39, -0.17) |
| Haiti | 605.00 (425.09, 834.91) | 966.34 (662.16, 1354.47) | 59.73% | 22.48 (15.80, 31.03) | 22.60 (15.48, 31.67) | 0.02 (0.01, 0.03) |
| Honduras | 399.86 (273.24, 577.44) | 643.73 (441.46, 912.11) | 60.99% | 18.12 (12.38, 26.17) | 19.51 (13.38, 27.65) | 0.26 (0.24, 0.28) |
| Hungary | 326.31 (230.26, 464.60) | 181.38 (124.48, 261.75) | -44.41% | 15.34 (10.82, 21.84) | 13.09 (8.98, 18.89) | -0.53 (-0.57, -0.49) |
| Iceland | 5.50 (3.39, 8.41) | 5.34 (3.27, 8.46) | -2.83% | 8.66 (5.34, 13.25) | 7.97 (4.88, 12.62) | -0.29 (-0.32, -0.26) |
| India | 61757.05 (41676.16, 88132.94) | 71495.07 (48463.18, 102208.25) | 15.77% | 18.65 (12.59, 26.62) | 19.01 (12.88, 27.17) | 0.08 (0.05, 0.11) |
| Indonesia | 14344.18 (10052.10, 20185.68) | 13803.88 (9672.15, 19381.20) | -3.77% | 21.06 (14.76, 29.64) | 21.20 (14.86, 29.77) | 0.03 (0.00, 0.06) |
| Iran (Islamic Republic of) | 4872.02 (3316.92, 6900.51) | 3843.93 (2619.66, 5449.48) | -21.10% | 18.80 (12.80, 26.63) | 18.84 (12.84, 26.72) | 0.01 (-0.02, 0.04) |
| Iraq | 1955.06 (1382.01, 2680.10) | 3047.75 (2144.49, 4239.34) | 55.89% | 24.19 (17.10, 33.17) | 22.12 (15.57, 30.77) | -0.31 (-0.35, -0.27) |
| Ireland | 100.69 (64.63, 154.92) | 92.78 (59.75, 143.57) | -7.86% | 10.26 (6.58, 15.78) | 9.09 (5.85, 14.07) | -0.42 (-0.47, -0.37) |
| Israel | 150.68 (92.84, 234.69) | 236.28 (144.81, 369.05) | 56.81% | 9.83 (6.06, 15.31) | 9.13 (5.59, 14.25) | -0.25 (-0.28, -0.22) |
| Italy | 911.52 (517.45, 1529.00) | 774.91 (482.29, 1217.45) | -14.99% | 9.88 (5.61, 16.57) | 9.75 (6.07, 15.31) | 0.00 (-0.21, 0.20) |
| Jamaica | 165.43 (112.62, 235.51) | 137.46 (95.13, 191.50) | -16.91% | 19.85 (13.51, 28.25) | 22.21 (15.37, 30.95) | 0.39 (0.38, 0.41) |
| Japan | 1962.28 (1233.44, 3156.46) | 1524.43 (968.69, 2317.64) | -22.31% | 8.51 (5.35, 13.68) | 9.77 (6.21, 14.85) | 0.52 (0.21, 0.83) |
| Jordan | 399.13 (281.51, 558.37) | 803.36 (559.54, 1131.77) | 101.28% | 23.99 (16.92, 33.56) | 21.58 (15.03, 30.40) | -0.36 (-0.40, -0.33) |
| Kazakhstan | 770.61 (529.08, 1093.23) | 668.79 (452.58, 961.51) | -13.21% | 14.92 (10.24, 21.16) | 13.18 (8.92, 18.95) | -0.40 (-0.45, -0.35) |
| Kenya | 3562.63 (2470.76, 5015.28) | 5824.30 (3995.03, 8109.28) | 63.48% | 31.74 (22.01, 44.68) | 30.40 (20.85, 42.32) | -0.14 (-0.17, -0.10) |
| Kiribati | 5.75 (4.08, 7.96) | 8.55 (6.06, 11.70) | 48.65% | 19.70 (13.98, 27.27) | 20.41 (14.46, 27.93) | 0.12 (0.09, 0.16) |
| Kuwait | 99.05 (66.43, 141.27) | 162.67 (111.40, 231.73) | 64.24% | 17.52 (11.75, 24.99) | 18.89 (12.93, 26.90) | 0.29 (0.22, 0.37) |
| Kyrgyzstan | 220.88 (148.44, 316.13) | 260.43 (174.67, 374.91) | 17.90% | 13.26 (8.91, 18.97) | 12.47 (8.37, 17.96) | -0.20 (-0.25, -0.16) |
| Lao People's Democratic Republic | 321.23 (221.47, 448.30) | 352.92 (238.95, 501.23) | 9.86% | 17.60 (12.14, 24.56) | 15.69 (10.62, 22.28) | -0.40 (-0.42, -0.39) |
| Latvia | 62.56 (41.25, 93.50) | 36.97 (24.60, 53.85) | -40.90% | 11.00 (7.25, 16.44) | 12.17 (8.10, 17.73) | 0.35 (0.30, 0.41) |
| Lebanon | 241.63 (166.91, 342.87) | 281.37 (195.76, 398.20) | 16.45% | 19.52 (13.49, 27.70) | 20.30 (14.12, 28.72) | 0.15 (0.09, 0.20) |
| Lesotho | 174.20 (119.16, 245.30) | 155.49 (106.72, 216.82) | -10.74% | 22.87 (15.65, 32.21) | 23.53 (16.15, 32.81) | 0.12 (0.08, 0.15) |
| Liberia | 207.18 (140.42, 294.72) | 470.69 (324.11, 659.21) | 127.19% | 22.88 (15.51, 32.55) | 25.20 (17.35, 35.29) | 0.33 (0.26, 0.40) |
| Libya | 307.58 (207.44, 440.36) | 292.75 (203.47, 414.35) | -4.82% | 16.34 (11.02, 23.39) | 19.93 (13.85, 28.21) | 0.69 (0.63, 0.74) |
| Lithuania | 113.36 (77.12, 162.93) | 55.09 (37.45, 79.79) | -51.41% | 13.68 (9.30, 19.66) | 13.25 (9.01, 19.19) | -0.11 (-0.18, -0.04) |
| Luxembourg | 5.79 (3.74, 9.02) | 8.21 (5.17, 12.83) | 41.72% | 8.78 (5.67, 13.67) | 8.31 (5.23, 12.99) | -0.19 (-0.20, -0.17) |
| Madagascar | 1605.27 (1142.06, 2181.63) | 3171.48 (2221.63, 4469.58) | 97.57% | 29.16 (20.75, 39.63) | 29.02 (20.33, 40.90) | -0.01 (-0.05, 0.02) |
| Malawi | 1374.50 (948.47, 1932.66) | 2349.46 (1620.03, 3266.89) | 70.93% | 31.03 (21.41, 43.63) | 29.82 (20.56, 41.47) | -0.13 (-0.18, -0.09) |
| Malaysia | 1225.92 (872.72, 1703.68) | 1318.74 (905.62, 1854.11) | 7.57% | 18.64 (13.27, 25.90) | 17.14 (11.77, 24.10) | -0.28 (-0.31, -0.26) |
| Maldives | 17.72 (12.01, 24.80) | 16.37 (10.97, 23.57) | -7.63% | 16.93 (11.48, 23.69) | 14.88 (9.97, 21.42) | -0.45 (-0.48, -0.41) |
| Mali | 940.02 (631.06, 1322.95) | 2292.27 (1548.84, 3280.21) | 143.85% | 22.79 (15.30, 32.08) | 22.20 (15.00, 31.76) | -0.09 (-0.12, -0.05) |
| Malta | 8.43 (5.40, 12.51) | 5.29 (3.38, 8.19) | -37.19% | 9.63 (6.17, 14.30) | 8.41 (5.38, 13.01) | -0.46 (-0.49, -0.44) |
| Marshall Islands | 3.97 (2.76, 5.57) | 3.41 (2.42, 4.69) | -14.28% | 17.92 (12.43, 25.12) | 18.47 (13.09, 25.42) | 0.10 (0.10, 0.11) |
| Mauritania | 242.45 (166.50, 337.09) | 389.38 (267.35, 551.16) | 60.60% | 25.91 (17.79, 36.03) | 24.15 (16.58, 34.18) | -0.24 (-0.27, -0.21) |
| Mauritius | 60.57 (42.25, 84.77) | 35.11 (24.00, 50.19) | -42.03% | 18.34 (12.79, 25.67) | 16.34 (11.17, 23.36) | -0.39 (-0.41, -0.37) |
| Mexico | 7273.13 (5055.21, 10229.12) | 6771.52 (4687.86, 9602.64) | -6.90% | 21.70 (15.08, 30.52) | 20.97 (14.52, 29.74) | -0.11 (-0.14, -0.09) |
| Micronesia (Federated States of) | 8.65 (6.11, 12.02) | 5.45 (3.83, 7.58) | -36.94% | 18.38 (12.99, 25.55) | 17.36 (12.19, 24.14) | -0.19 (-0.21, -0.17) |
| Monaco | 0.34 (0.22, 0.52) | 0.43 (0.27, 0.66) | 27.04% | 9.68 (6.17, 14.72) | 8.75 (5.53, 13.38) | -0.35 (-0.37, -0.32) |
| Mongolia | 116.64 (78.73, 164.30) | 140.19 (94.51, 200.94) | 20.19% | 12.99 (8.77, 18.30) | 13.85 (9.34, 19.85) | 0.24 (0.16, 0.31) |
| Montenegro | 19.01 (12.98, 27.37) | 11.46 (7.62, 17.12) | -39.68% | 11.75 (8.02, 16.92) | 10.52 (6.99, 15.70) | -0.38 (-0.41, -0.36) |
| Morocco | 2023.49 (1410.13, 2811.82) | 1924.81 (1344.37, 2683.52) | -4.88% | 20.66 (14.39, 28.70) | 20.40 (14.25, 28.44) | -0.04 (-0.08, -0.01) |
| Mozambique | 2043.65 (1426.08, 2891.84) | 4915.12 (3414.16, 6833.46) | 140.51% | 33.54 (23.41, 47.46) | 35.71 (24.81, 49.65) | 0.25 (0.18, 0.33) |
| Myanmar | 2631.99 (1766.76, 3722.78) | 2386.64 (1601.90, 3432.29) | -9.32% | 16.78 (11.27, 23.74) | 15.93 (10.70, 22.92) | -0.18 (-0.22, -0.13) |
| Namibia | 150.79 (104.99, 209.16) | 196.38 (134.70, 272.99) | 30.24% | 25.34 (17.64, 35.15) | 23.45 (16.08, 32.59) | -0.26 (-0.32, -0.21) |
| Nauru | 0.83 (0.58, 1.16) | 0.75 (0.53, 1.05) | -10.02% | 18.74 (13.15, 26.07) | 19.13 (13.44, 26.78) | 0.07 (0.06, 0.08) |
| Nepal | 1300.56 (835.71, 1927.88) | 1289.81 (839.45, 1895.76) | -0.83% | 15.30 (9.83, 22.68) | 13.99 (9.11, 20.56) | -0.31 (-0.34, -0.28) |
| Netherlands | 269.22 (173.94, 405.19) | 272.54 (174.31, 410.43) | 1.23% | 9.88 (6.38, 14.87) | 9.97 (6.38, 15.01) | 0.05 (0.01, 0.08) |
| New Zealand | 84.23 (50.70, 135.31) | 118.30 (78.08, 177.62) | 40.44% | 10.53 (6.34, 16.91) | 13.26 (8.75, 19.91) | 0.82 (0.74, 0.90) |
| Nicaragua | 389.24 (269.35, 544.23) | 406.14 (278.04, 569.80) | 4.34% | 21.31 (14.75, 29.80) | 20.30 (13.90, 28.48) | -0.17 (-0.17, -0.16) |
| Niger | 902.68 (610.97, 1289.36) | 2844.28 (1945.22, 4042.01) | 215.09% | 22.26 (15.06, 31.79) | 23.66 (16.18, 33.62) | 0.22 (0.18, 0.26) |
| Nigeria | 9211.52 (6272.66, 13010.65) | 23199.82 (15574.49, 32963.60) | 151.86% | 23.39 (15.93, 33.03) | 24.75 (16.61, 35.16) | 0.21 (0.16, 0.26) |
| Niue | 0.15 (0.10, 0.21) | 0.07 (0.05, 0.10) | -50.63% | 18.15 (12.58, 25.41) | 18.44 (12.92, 25.69) | 0.06 (0.05, 0.08) |
| North Macedonia | 58.75 (39.34, 86.42) | 33.06 (21.75, 49.16) | -43.73% | 10.96 (7.34, 16.12) | 9.57 (6.30, 14.23) | -0.47 (-0.49, -0.44) |
| Northern Mariana Islands | 2.02 (1.39, 2.80) | 1.29 (0.89, 1.81) | -36.25% | 16.28 (11.22, 22.54) | 17.44 (12.09, 24.50) | 0.25 (0.21, 0.29) |
| Norway | 102.79 (65.44, 155.70) | 160.22 (107.58, 233.66) | 55.87% | 12.86 (8.19, 19.48) | 17.13 (11.50, 24.98) | 0.99 (0.80, 1.17) |
| Oman | 136.29 (91.48, 193.82) | 199.88 (139.59, 280.62) | 46.65% | 16.36 (10.98, 23.26) | 19.48 (13.60, 27.35) | 0.61 (0.53, 0.69) |
| Pakistan | 10560.98 (7291.36, 14867.34) | 18719.87 (12939.42, 26288.41) | 77.26% | 20.82 (14.38, 29.32) | 21.48 (14.85, 30.17) | 0.12 (0.07, 0.17) |
| Palau | 0.88 (0.61, 1.21) | 0.62 (0.44, 0.88) | -28.86% | 18.49 (12.88, 25.61) | 18.51 (12.94, 26.07) | 0.00 (-0.01, 0.01) |
| Palestine | 185.86 (126.08, 260.48) | 347.31 (232.42, 497.20) | 86.87% | 18.67 (12.67, 26.17) | 18.72 (12.53, 26.79) | 0.01 (-0.02, 0.05) |
| Panama | 148.14 (98.88, 212.62) | 200.86 (138.42, 286.88) | 35.59% | 17.77 (11.86, 25.50) | 17.56 (12.10, 25.08) | -0.04 (-0.06, -0.02) |
| Papua New Guinea | 309.32 (214.40, 429.51) | 682.55 (470.13, 951.93) | 120.66% | 18.43 (12.77, 25.59) | 18.54 (12.77, 25.86) | 0.02 (0.02, 0.03) |
| Paraguay | 348.18 (241.84, 487.85) | 398.91 (278.55, 558.79) | 14.57% | 20.81 (14.46, 29.16) | 20.27 (14.15, 28.39) | -0.09 (-0.12, -0.05) |
| Peru | 1514.29 (1038.91, 2143.57) | 1601.82 (1093.15, 2279.59) | 5.78% | 18.02 (12.36, 25.51) | 17.54 (11.97, 24.96) | -0.09 (-0.15, -0.03) |
| Philippines | 4208.21 (2860.50, 5961.89) | 6259.33 (4301.08, 8843.92) | 48.74% | 16.55 (11.25, 23.45) | 17.60 (12.09, 24.87) | 0.22 (0.18, 0.26) |
| Poland | 1174.16 (778.16, 1739.49) | 696.10 (461.41, 1031.99) | -40.72% | 12.30 (8.15, 18.22) | 11.82 (7.84, 17.52) | -0.13 (-0.21, -0.04) |
| Portugal | 213.57 (137.38, 332.00) | 119.03 (74.67, 189.61) | -44.27% | 10.10 (6.50, 15.70) | 8.54 (5.36, 13.61) | -0.56 (-0.61, -0.52) |
| Puerto Rico | 198.11 (136.72, 277.51) | 103.48 (71.19, 143.77) | -47.77% | 19.89 (13.72, 27.86) | 19.77 (13.60, 27.47) | -0.01 (-0.04, 0.01) |
| Qatar | 26.17 (18.55, 36.15) | 80.13 (55.26, 113.49) | 206.23% | 21.02 (14.90, 29.04) | 19.51 (13.45, 27.62) | -0.26 (-0.28, -0.23) |
| Republic of Korea | 1309.03 (860.56, 1910.52) | 593.04 (377.81, 912.89) | -54.70% | 11.45 (7.53, 16.71) | 8.65 (5.51, 13.31) | -0.96 (-1.00, -0.92) |
| Republic of Moldova | 158.22 (106.76, 229.78) | 77.06 (51.78, 111.85) | -51.30% | 12.85 (8.67, 18.66) | 13.35 (8.97, 19.37) | 0.12 (0.08, 0.17) |
| Romania | 689.08 (469.72, 998.60) | 328.19 (217.24, 489.85) | -52.37% | 12.36 (8.42, 17.91) | 10.83 (7.17, 16.17) | -0.45 (-0.48, -0.43) |
| Russian Federation | 4474.85 (2989.54, 6560.94) | 3845.79 (2631.18, 5532.89) | -14.06% | 12.89 (8.61, 18.90) | 14.25 (9.75, 20.50) | 0.33 (0.24, 0.42) |
| Rwanda | 970.60 (674.40, 1361.30) | 1167.61 (798.50, 1647.49) | 20.30% | 28.70 (19.94, 40.25) | 24.11 (16.49, 34.01) | -0.61 (-0.70, -0.53) |
| Saint Kitts and Nevis | 2.99 (2.07, 4.14) | 2.49 (1.72, 3.47) | -16.93% | 21.35 (14.72, 29.54) | 21.05 (14.54, 29.39) | -0.04 (-0.07, -0.02) |
| Saint Lucia | 12.62 (8.81, 17.38) | 7.07 (4.91, 9.84) | -43.97% | 24.09 (16.81, 33.17) | 22.23 (15.45, 30.96) | -0.27 (-0.32, -0.23) |
| Saint Vincent and the Grenadines | 8.79 (6.07, 12.28) | 5.52 (3.86, 7.76) | -37.16% | 21.09 (14.57, 29.48) | 22.08 (15.43, 31.03) | 0.16 (0.14, 0.18) |
| Samoa | 12.63 (8.88, 17.64) | 14.00 (9.88, 19.58) | 10.85% | 19.18 (13.48, 26.79) | 19.01 (13.41, 26.58) | -0.03 (-0.07, 0.01) |
| San Marino | 0.45 (0.28, 0.68) | 0.47 (0.29, 0.73) | 5.12% | 9.75 (6.06, 14.81) | 8.76 (5.47, 13.57) | -0.37 (-0.40, -0.33) |
| Sao Tome and Principe | 13.28 (9.28, 18.73) | 19.19 (13.43, 26.69) | 44.53% | 23.34 (16.31, 32.93) | 25.80 (18.05, 35.87) | 0.35 (0.31, 0.38) |
| Saudi Arabia | 1018.30 (696.14, 1440.38) | 1271.41 (868.69, 1801.08) | 24.86% | 15.26 (10.43, 21.59) | 18.04 (12.33, 25.56) | 0.58 (0.51, 0.65) |
| Senegal | 931.24 (643.98, 1292.31) | 1525.90 (1048.14, 2149.85) | 63.86% | 25.58 (17.69, 35.50) | 25.01 (17.18, 35.23) | -0.07 (-0.10, -0.04) |
| Serbia | 253.53 (168.34, 375.99) | 163.76 (109.04, 240.22) | -35.41% | 12.01 (7.98, 17.82) | 11.19 (7.45, 16.41) | -0.24 (-0.27, -0.22) |
| Seychelles | 4.12 (2.87, 5.76) | 3.72 (2.56, 5.24) | -9.83% | 17.28 (12.01, 24.13) | 17.04 (11.72, 24.00) | -0.05 (-0.09, 0.00) |
| Sierra Leone | 415.22 (285.06, 585.76) | 916.26 (626.44, 1292.42) | 120.67% | 26.02 (17.86, 36.70) | 27.42 (18.75, 38.68) | 0.20 (0.14, 0.25) |
| Singapore | 63.43 (40.70, 94.27) | 59.47 (37.06, 93.24) | -6.24% | 9.77 (6.27, 14.52) | 7.61 (4.74, 11.92) | -0.86 (-0.90, -0.83) |
| Slovakia | 166.89 (113.01, 241.85) | 88.45 (57.75, 131.81) | -47.00% | 12.60 (8.53, 18.25) | 10.41 (6.80, 15.51) | -0.66 (-0.68, -0.63) |
| Slovenia | 40.09 (26.16, 59.85) | 24.51 (15.84, 37.34) | -38.85% | 9.70 (6.33, 14.48) | 7.89 (5.10, 12.02) | -0.70 (-0.75, -0.65) |
| Solomon Islands | 28.83 (19.91, 40.32) | 45.98 (31.69, 64.63) | 59.46% | 18.35 (12.67, 25.65) | 17.80 (12.27, 25.02) | -0.11 (-0.11, -0.10) |
| Somalia | 987.27 (672.34, 1369.90) | 2969.90 (2012.19, 4151.56) | 200.82% | 28.47 (19.39, 39.50) | 31.26 (21.18, 43.70) | 0.33 (0.28, 0.39) |
| South Africa | 3923.87 (2701.55, 5520.73) | 4152.25 (2809.35, 5910.92) | 5.82% | 29.63 (20.40, 41.69) | 27.62 (18.68, 39.31) | -0.21 (-0.36, -0.07) |
| South Sudan | 691.39 (482.06, 975.70) | 1168.46 (804.42, 1660.11) | 69.00% | 26.50 (18.48, 37.40) | 28.03 (19.30, 39.83) | 0.19 (0.16, 0.22) |
| Spain | 751.67 (472.26, 1180.66) | 592.99 (363.14, 919.82) | -21.11% | 9.59 (6.03, 15.07) | 8.75 (5.36, 13.57) | -0.31 (-0.39, -0.24) |
| Sri Lanka | 849.05 (577.31, 1210.80) | 767.71 (515.89, 1094.88) | -9.58% | 15.13 (10.29, 21.58) | 15.09 (10.14, 21.52) | -0.01 (-0.02, 0.01) |
| Sudan | 1752.45 (1221.02, 2460.69) | 3238.74 (2259.17, 4547.26) | 84.81% | 19.30 (13.45, 27.11) | 20.50 (14.30, 28.78) | 0.20 (0.14, 0.26) |
| Suriname | 27.40 (18.79, 38.18) | 32.86 (22.77, 46.06) | 19.90% | 20.96 (14.37, 29.20) | 22.41 (15.53, 31.41) | 0.24 (0.19, 0.29) |
| Sweden | 208.11 (131.31, 313.42) | 304.51 (199.39, 441.32) | 46.32% | 13.47 (8.50, 20.29) | 16.85 (11.03, 24.42) | 0.77 (0.71, 0.83) |
| Switzerland | 96.24 (60.14, 149.84) | 97.38 (59.68, 156.03) | 1.18% | 8.34 (5.21, 12.98) | 7.42 (4.55, 11.89) | -0.39 (-0.43, -0.36) |
| Syrian Arab Republic | 1236.99 (884.12, 1714.80) | 867.62 (621.13, 1183.20) | -29.86% | 20.35 (14.55, 28.21) | 21.42 (15.33, 29.21) | 0.18 (0.15, 0.21) |
| Taiwan (Province of China) | 771.27 (533.25, 1091.15) | 441.53 (296.89, 640.16) | -42.75% | 13.98 (9.66, 19.78) | 14.59 (9.81, 21.15) | 0.14 (0.07, 0.21) |
| Tajikistan | 289.10 (196.54, 413.17) | 408.55 (268.95, 587.61) | 41.32% | 12.48 (8.49, 17.84) | 12.56 (8.27, 18.06) | 0.03 (0.00, 0.05) |
| Thailand | 2454.13 (1679.25, 3526.59) | 1482.80 (1007.36, 2142.69) | -39.58% | 14.43 (9.87, 20.73) | 13.91 (9.45, 20.09) | -0.13 (-0.15, -0.11) |
| Timor-Leste | 54.66 (37.29, 76.69) | 83.32 (57.32, 117.89) | 52.41% | 16.34 (11.15, 22.92) | 16.51 (11.36, 23.36) | 0.04 (0.02, 0.06) |
| Togo | 448.45 (311.63, 624.27) | 804.87 (555.34, 1150.94) | 79.48% | 25.21 (17.52, 35.09) | 25.57 (17.64, 36.57) | 0.06 (0.03, 0.08) |
| Tokelau | 0.13 (0.09, 0.18) | 0.09 (0.06, 0.12) | -34.47% | 18.67 (12.96, 26.10) | 18.41 (12.69, 25.99) | -0.04 (-0.08, -0.01) |
| Tonga | 7.18 (4.87, 10.14) | 6.64 (4.61, 9.37) | -7.51% | 18.06 (12.26, 25.52) | 18.50 (12.84, 26.10) | 0.09 (0.06, 0.11) |
| Trinidad and Tobago | 97.86 (66.84, 136.49) | 63.79 (44.56, 89.34) | -34.81% | 24.22 (16.54, 33.78) | 23.46 (16.39, 32.86) | -0.11 (-0.15, -0.07) |
| Tunisia | 535.85 (358.20, 777.17) | 473.86 (318.89, 674.48) | -11.57% | 16.82 (11.24, 24.39) | 17.91 (12.05, 25.49) | 0.22 (0.20, 0.24) |
| Turkey | 4111.34 (2839.37, 5818.37) | 2722.74 (1853.40, 3862.46) | -33.77% | 19.02 (13.14, 26.92) | 17.27 (11.75, 24.49) | -0.33 (-0.35, -0.31) |
| Turkmenistan | 173.41 (116.80, 249.49) | 188.85 (128.47, 268.23) | 8.90% | 11.53 (7.77, 16.59) | 12.45 (8.47, 17.69) | 0.27 (0.22, 0.32) |
| Tuvalu | 0.61 (0.42, 0.86) | 0.63 (0.44, 0.89) | 2.96% | 18.52 (12.84, 25.93) | 18.39 (12.83, 25.93) | -0.02 (-0.05, 0.00) |
| Uganda | 2310.96 (1612.62, 3212.32) | 4765.20 (3219.67, 6737.26) | 106.20% | 27.39 (19.12, 38.08) | 24.98 (16.88, 35.32) | -0.32 (-0.34, -0.30) |
| Ukraine | 1990.32 (1386.47, 2854.11) | 1422.81 (1009.64, 1997.26) | -28.51% | 17.55 (12.23, 25.17) | 20.60 (14.62, 28.91) | 0.56 (0.51, 0.62) |
| United Arab Emirates | 122.52 (85.00, 172.06) | 239.53 (168.35, 336.20) | 95.50% | 20.73 (14.38, 29.11) | 20.43 (14.36, 28.68) | -0.05 (-0.09, -0.01) |
| United Kingdom | 1202.89 (771.17, 1825.09) | 1172.26 (741.51, 1787.47) | -2.55% | 10.98 (7.04, 16.66) | 9.89 (6.26, 15.08) | -0.36 (-0.39, -0.33) |
| United Republic of Tanzania | 3402.99 (2329.26, 4789.32) | 7412.36 (5126.69, 10549.23) | 117.82% | 28.04 (19.19, 39.46) | 30.16 (20.86, 42.93) | 0.25 (0.23, 0.27) |
| United States of America | 5883.37 (3697.43, 9096.56) | 7754.80 (4996.40, 11837.63) | 31.81% | 10.57 (6.64, 16.35) | 12.89 (8.31, 19.68) | 0.69 (0.64, 0.73) |
| United States Virgin Islands | 6.13 (4.19, 8.68) | 3.95 (2.73, 5.59) | -35.62% | 19.26 (13.15, 27.26) | 19.71 (13.62, 27.90) | 0.08 (0.06, 0.10) |
| Uruguay | 95.49 (63.96, 141.78) | 80.02 (52.76, 117.56) | -16.20% | 11.67 (7.82, 17.32) | 11.41 (7.52, 16.76) | -0.08 (-0.09, -0.07) |
| Uzbekistan | 1040.27 (696.93, 1505.70) | 1245.32 (827.99, 1821.44) | 19.71% | 12.14 (8.13, 17.57) | 12.10 (8.04, 17.69) | -0.01 (-0.06, 0.04) |
| Vanuatu | 14.77 (10.47, 20.36) | 23.45 (16.62, 32.08) | 58.80% | 22.07 (15.64, 30.43) | 21.16 (15.00, 28.95) | -0.15 (-0.17, -0.12) |
| Venezuela (Bolivarian Republic of) | 1563.27 (1087.14, 2143.64) | 1391.52 (953.85, 1965.09) | -10.99% | 21.99 (15.29, 30.15) | 19.76 (13.55, 27.91) | -0.36 (-0.40, -0.32) |
| Viet Nam | 3903.52 (2620.70, 5638.37) | 3467.67 (2370.24, 4917.74) | -11.17% | 14.90 (10.00, 21.52) | 16.26 (11.12, 23.06) | 0.35 (0.09, 0.61) |
| Yemen | 1374.36 (929.23, 1956.42) | 2584.44 (1761.38, 3626.14) | 88.05% | 19.14 (12.94, 27.24) | 19.96 (13.60, 28.01) | 0.15 (0.14, 0.16) |
| Zambia | 1035.32 (719.17, 1468.86) | 2122.71 (1443.35, 3005.83) | 105.03% | 27.52 (19.12, 39.05) | 27.45 (18.66, 38.87) | -0.02 (-0.04, 0.01) |
| Zimbabwe | 1102.74 (758.54, 1557.29) | 1479.50 (1015.42, 2041.08) | 34.17% | 22.89 (15.74, 32.32) | 24.80 (17.02, 34.21) | 0.29 (0.22, 0.37) |

Note: ASR: age-standardized rates; AAPC, average annual percentage change; CI, confidence interval; UI, uncertainty interval

Table S4**. The prevalent strokes and age-standardized prevalence rate for stroke and their temporal change among children (0-14 years) in 204 countries or territories from 1990 to 2019.**

|  | Prevalent strokes (95% UI) | | | Age-standardized prevalence rate (95% UI), per 100,000 population | | |
| --- | --- | --- | --- | --- | --- | --- |
| **Countries or territories** | 1990 | 2019 | Percentage change (%) | 1990 | 2019 | AAPC (95% CI) |
| Afghanistan | 3717.19 (2693.67, 4869.35) | 13995.82 (10297.40, 18489.84) | 276.52% | 74.50 (53.99, 97.59) | 81.09 (59.67, 107.13) | 0.31 (-0.06, 0.67) |
| Albania | 302.33 (184.25, 436.58) | 112.62 (66.38, 165.97) | -62.75% | 26.91 (16.40, 38.85) | 23.81 (14.04, 35.10) | 0.25 (-3.26, 3.89) |
| Algeria | 7990.89 (5597.21, 10462.85) | 8540.16 (6252.64, 11385.38) | 6.87% | 74.54 (52.21, 97.60) | 72.76 (53.27, 97.00) | -0.06 (-0.27, 0.15) |
| American Samoa | 12.46 (8.72, 16.68) | 13.01 (9.16, 17.14) | 4.37% | 65.92 (46.13, 88.20) | 78.35 (55.18, 103.24) | 0.63 (0.56, 0.70) |
| Andorra | 3.37 (2.08, 4.86) | 3.94 (2.45, 5.88) | 16.76% | 35.53 (21.91, 51.12) | 36.84 (22.88, 54.99) | 0.22 (-0.07, 0.51) |
| Angola | 4784.19 (3573.53, 6232.55) | 15116.90 (11073.68, 19504.70) | 215.98% | 100.76 (75.26, 131.26) | 108.08 (79.17, 139.45) | 0.28 (0.09, 0.46) |
| Antigua and Barbuda | 18.09 (12.92, 23.41) | 17.53 (12.78, 23.10) | -3.05% | 96.72 (69.07, 125.19) | 103.86 (75.71, 136.81) | 0.25 (0.16, 0.33) |
| Argentina | 2520.98 (1491.93, 3658.46) | 2553.27 (1487.47, 3860.30) | 1.28% | 24.88 (14.72, 36.10) | 24.14 (14.07, 36.50) | -0.08 (-0.21, 0.06) |
| Armenia | 337.37 (205.05, 493.74) | 195.37 (118.36, 288.01) | -42.09% | 32.39 (19.69, 47.40) | 32.10 (19.45, 47.33) | -0.01 (-0.25, 0.24) |
| Australia | 1522.31 (961.54, 2143.32) | 1687.20 (1046.71, 2423.89) | 10.83% | 40.20 (25.39, 56.60) | 36.75 (22.80, 52.79) | -0.28 (-0.58, 0.02) |
| Austria | 590.03 (366.38, 865.06) | 496.78 (312.62, 720.38) | -15.80% | 43.74 (27.16, 64.13) | 38.54 (24.25, 55.88) | -0.28 (-1.03, 0.48) |
| Azerbaijan | 677.52 (397.71, 999.59) | 699.90 (437.64, 1010.11) | 3.30% | 27.89 (16.37, 41.14) | 30.08 (18.81, 43.41) | 0.36 (-0.43, 1.15) |
| Bahamas | 88.36 (66.35, 113.17) | 85.77 (64.31, 112.55) | -2.93% | 109.89 (82.53, 140.76) | 107.33 (80.48, 140.84) | -0.09 (-0.24, 0.07) |
| Bahrain | 129.22 (93.86, 171.80) | 200.45 (144.39, 263.33) | 55.12% | 78.79 (57.23, 104.75) | 85.37 (61.49, 112.15) | 0.30 (0.16, 0.43) |
| Bangladesh | 29521.22 (20725.50, 39989.40) | 30777.25 (21526.51, 43323.22) | 4.25% | 60.95 (42.79, 82.56) | 69.87 (48.87, 98.35) | 0.86 (-0.77, 2.51) |
| Barbados | 73.07 (54.82, 93.37) | 60.63 (44.90, 79.86) | -17.02% | 116.42 (87.35, 148.77) | 122.73 (90.89, 161.65) | 0.19 (0.01, 0.36) |
| Belarus | 1073.54 (700.18, 1496.73) | 770.27 (525.58, 1071.10) | -28.25% | 44.49 (29.02, 62.03) | 46.85 (31.97, 65.15) | 0.21 (-0.33, 0.75) |
| Belgium | 602.66 (373.39, 876.15) | 672.51 (391.07, 994.26) | 11.59% | 33.36 (20.67, 48.50) | 34.79 (20.23, 51.43) | 0.20 (-0.20, 0.60) |
| Belize | 72.75 (53.31, 94.68) | 132.10 (96.30, 169.10) | 81.57% | 90.05 (65.98, 117.20) | 107.84 (78.61, 138.05) | 0.62 (0.47, 0.77) |
| Benin | 2264.00 (1641.50, 2975.52) | 5845.73 (4274.40, 7593.43) | 158.20% | 93.38 (67.70, 122.72) | 101.46 (74.19, 131.79) | 0.38 (0.09, 0.68) |
| Bermuda | 11.94 (8.83, 15.28) | 8.99 (6.76, 11.68) | -24.68% | 100.02 (73.97, 128.02) | 102.05 (76.66, 132.57) | 0.06 (-0.03, 0.16) |
| Bhutan | 135.61 (92.00, 187.72) | 108.01 (71.78, 146.90) | -20.35% | 53.56 (36.33, 74.14) | 55.50 (36.88, 75.48) | 0.21 (-0.44, 0.86) |
| Bolivia (Plurinational State of) | 1725.51 (1249.67, 2272.97) | 2526.70 (1787.72, 3358.40) | 46.43% | 63.20 (45.77, 83.25) | 64.36 (45.53, 85.54) | 0.10 (-0.29, 0.48) |
| Bosnia and Herzegovina | 490.02 (326.45, 687.21) | 217.59 (134.92, 311.75) | -55.59% | 44.35 (29.54, 62.19) | 45.66 (28.31, 65.42) | 0.27 (-0.86, 1.42) |
| Botswana | 788.05 (599.87, 1000.02) | 977.27 (747.37, 1253.49) | 24.01% | 137.92 (104.99, 175.02) | 138.58 (105.98, 177.74) | 0.05 (-0.03, 0.13) |
| Brazil | 53110.29 (38548.17, 73110.60) | 43858.78 (32718.60, 59211.16) | -17.42% | 101.60 (73.75, 139.87) | 91.86 (68.53, 124.02) | -0.34 (-0.50, -0.18) |
| Brunei Darussalam | 35.39 (23.76, 50.12) | 41.20 (26.16, 57.82) | 16.41% | 39.04 (26.21, 55.27) | 43.31 (27.50, 60.78) | 0.46 (-0.77, 1.70) |
| Bulgaria | 974.11 (635.72, 1438.03) | 509.05 (326.11, 714.84) | -47.74% | 56.09 (36.61, 82.80) | 51.43 (32.95, 72.22) | -0.06 (-2.01, 1.92) |
| Burkina Faso | 4376.64 (3203.33, 5731.74) | 9454.89 (6771.38, 12505.78) | 116.03% | 92.56 (67.75, 121.22) | 90.97 (65.15, 120.32) | 0.01 (-0.40, 0.42) |
| Burundi | 4080.19 (3145.02, 5166.36) | 8688.13 (6674.87, 11177.86) | 112.93% | 154.61 (119.17, 195.77) | 160.85 (123.58, 206.94) | 0.18 (-0.02, 0.39) |
| Cabo Verde | 171.63 (127.51, 222.18) | 190.68 (144.54, 242.47) | 11.10% | 110.64 (82.20, 143.22) | 119.94 (90.91, 152.51) | 0.28 (0.20, 0.37) |
| Cambodia | 1591.80 (1020.88, 2264.55) | 1971.56 (1290.92, 2792.04) | 23.86% | 33.37 (21.40, 47.48) | 39.17 (25.65, 55.47) | 0.63 (0.01, 1.25) |
| Cameroon | 4265.83 (3133.56, 5516.57) | 12307.98 (9146.41, 15901.60) | 188.52% | 88.15 (64.75, 114.00) | 102.24 (75.98, 132.10) | 0.52 (0.35, 0.70) |
| Canada | 3864.50 (2508.33, 5563.51) | 3603.49 (2353.12, 5195.12) | -6.75% | 67.17 (43.60, 96.70) | 59.05 (38.56, 85.13) | -0.25 (-1.69, 1.21) |
| Central African Republic | 1257.01 (933.14, 1637.94) | 2383.64 (1761.39, 3055.22) | 89.63% | 102.65 (76.20, 133.75) | 106.73 (78.87, 136.80) | 0.14 (0.01, 0.28) |
| Chad | 2646.71 (1994.49, 3452.94) | 8274.03 (6235.01, 10607.18) | 212.62% | 90.56 (68.25, 118.15) | 99.57 (75.03, 127.64) | 0.38 (0.14, 0.63) |
| Chile | 1168.77 (736.52, 1709.24) | 1157.16 (727.51, 1675.43) | -0.99% | 29.38 (18.52, 42.97) | 31.89 (20.05, 46.17) | 0.21 (-0.09, 0.52) |
| China | 196869.01 (134988.77, 277920.70) | 110147.48 (74347.81, 158611.31) | -44.05% | 60.97 (41.80, 86.07) | 49.00 (33.08, 70.56) | -0.69 (-0.95, -0.42) |
| Colombia | 10159.58 (7290.53, 13344.31) | 8873.53 (6413.46, 11606.71) | -12.66% | 86.76 (62.26, 113.95) | 80.49 (58.17, 105.28) | -0.29 (-0.65, 0.08) |
| Comoros | 406.62 (316.29, 509.28) | 392.54 (301.86, 498.43) | -3.46% | 191.39 (148.88, 239.72) | 168.63 (129.67, 214.11) | -0.35 (-0.54, -0.17) |
| Congo | 1396.66 (1053.26, 1793.44) | 2432.62 (1840.17, 3150.56) | 74.17% | 127.42 (96.09, 163.62) | 122.37 (92.57, 158.49) | -0.08 (-0.25, 0.08) |
| Cook Islands | 4.64 (3.26, 6.28) | 3.15 (2.23, 4.26) | -32.17% | 69.18 (48.59, 93.53) | 75.28 (53.25, 101.84) | 0.31 (0.19, 0.42) |
| Costa Rica | 1007.20 (711.73, 1337.93) | 950.79 (685.65, 1258.09) | -5.60% | 89.71 (63.39, 119.16) | 90.13 (65.00, 119.26) | 0.00 (-0.11, 0.12) |
| Croatia | 363.38 (219.88, 540.30) | 195.07 (115.71, 287.30) | -46.32% | 36.82 (22.28, 54.75) | 32.00 (18.98, 47.13) | -0.43 (-0.73, -0.12) |
| Cuba | 2608.05 (1909.91, 3396.52) | 1695.43 (1254.22, 2250.61) | -34.99% | 104.37 (76.43, 135.92) | 94.43 (69.86, 125.36) | -0.34 (-0.52, -0.16) |
| Cyprus | 59.49 (34.34, 90.04) | 73.44 (42.43, 110.41) | 23.46% | 30.03 (17.33, 45.45) | 34.47 (19.91, 51.82) | 0.53 (0.20, 0.86) |
| Czechia | 1120.69 (744.66, 1570.54) | 631.02 (406.26, 885.31) | -43.69% | 50.99 (33.88, 71.46) | 37.02 (23.84, 51.94) | -1.01 (-1.55, -0.47) |
| Cote d'Ivoire | 5993.91 (4428.93, 7663.24) | 11903.25 (8987.19, 15365.51) | 98.59% | 104.61 (77.30, 133.74) | 111.42 (84.12, 143.83) | 0.24 (0.09, 0.39) |
| Democratic People's Republic of Korea | 2362.25 (1530.09, 3228.77) | 2551.47 (1718.07, 3516.46) | 8.01% | 36.28 (23.50, 49.58) | 52.07 (35.06, 71.76) | 1.39 (0.56, 2.24) |
| Democratic Republic of the Congo | 16804.33 (12471.69, 21775.98) | 35665.35 (26422.33, 47672.52) | 112.24% | 92.64 (68.75, 120.05) | 94.62 (70.09, 126.47) | 0.16 (-0.17, 0.48) |
| Denmark | 435.20 (282.14, 605.42) | 388.85 (243.44, 552.31) | -10.65% | 49.27 (31.94, 68.55) | 40.50 (25.35, 57.52) | -0.48 (-1.08, 0.12) |
| Djibouti | 321.55 (251.96, 405.89) | 673.62 (517.72, 856.48) | 109.49% | 146.31 (114.64, 184.69) | 160.16 (123.09, 203.64) | 0.35 (0.16, 0.53) |
| Dominica | 25.58 (18.75, 33.89) | 16.08 (11.98, 21.18) | -37.12% | 104.23 (76.40, 138.10) | 111.04 (82.71, 146.21) | 0.23 (0.13, 0.34) |
| Dominican Republic | 2232.52 (1613.62, 2922.48) | 2647.24 (1953.10, 3470.03) | 18.58% | 81.38 (58.82, 106.53) | 86.97 (64.17, 114.00) | 0.24 (0.10, 0.38) |
| Ecuador | 2898.93 (2084.83, 3892.68) | 4049.65 (2903.64, 5347.04) | 39.69% | 74.14 (53.32, 99.55) | 80.46 (57.69, 106.24) | 0.29 (0.18, 0.41) |
| Egypt | 15630.47 (11214.01, 21193.99) | 26774.91 (19622.56, 35179.95) | 71.30% | 70.22 (50.38, 95.22) | 81.99 (60.09, 107.72) | 0.57 (0.38, 0.75) |
| El Salvador | 1478.90 (1051.86, 1928.93) | 1277.45 (908.31, 1698.14) | -13.62% | 69.60 (49.50, 90.78) | 74.84 (53.21, 99.48) | 0.30 (0.18, 0.43) |
| Equatorial Guinea | 207.25 (154.31, 270.41) | 647.49 (487.30, 847.95) | 212.42% | 100.93 (75.14, 131.68) | 114.72 (86.34, 150.24) | 0.48 (0.27, 0.69) |
| Eritrea | 1898.42 (1428.90, 2423.26) | 3654.35 (2792.98, 4731.77) | 92.49% | 134.78 (101.44, 172.04) | 138.37 (105.75, 179.17) | 0.11 (-0.07, 0.30) |
| Estonia | 111.49 (66.63, 164.27) | 72.11 (43.10, 103.63) | -35.33% | 31.92 (19.08, 47.03) | 33.45 (20.00, 48.07) | 0.19 (-0.10, 0.47) |
| Eswatini | 470.84 (358.96, 607.13) | 498.25 (369.40, 642.94) | 5.82% | 122.18 (93.15, 157.55) | 121.76 (90.27, 157.12) | 0.00 (-0.12, 0.12) |
| Ethiopia | 27141.38 (19670.82, 38079.12) | 57951.99 (41126.61, 79560.71) | 113.52% | 109.55 (79.39, 153.69) | 125.80 (89.28, 172.71) | 0.49 (0.38, 0.60) |
| Fiji | 205.94 (144.71, 275.15) | 201.43 (142.09, 272.42) | -2.19% | 73.01 (51.30, 97.54) | 75.87 (53.52, 102.61) | 0.21 (0.10, 0.33) |
| Finland | 547.82 (365.13, 749.89) | 495.49 (327.94, 700.26) | -9.55% | 56.74 (37.82, 77.66) | 56.66 (37.50, 80.08) | 0.18 (-0.65, 1.02) |
| France | 4138.00 (2599.54, 6193.51) | 3879.13 (2342.45, 5677.18) | -6.26% | 35.34 (22.20, 52.89) | 32.84 (19.83, 48.07) | -0.21 (-0.45, 0.04) |
| Gabon | 463.52 (352.49, 590.79) | 680.70 (507.18, 885.37) | 46.85% | 110.65 (84.14, 141.02) | 116.84 (87.06, 151.97) | 0.20 (0.15, 0.26) |
| Gambia | 556.88 (418.44, 725.70) | 1206.97 (907.85, 1565.87) | 116.74% | 118.27 (88.87, 154.12) | 130.50 (98.16, 169.30) | 0.34 (0.17, 0.50) |
| Georgia | 501.94 (317.89, 739.46) | 269.11 (164.86, 396.67) | -46.39% | 36.73 (23.26, 54.11) | 37.11 (22.74, 54.71) | 0.06 (-0.25, 0.37) |
| Germany | 5492.75 (3495.93, 7817.09) | 4615.65 (3000.12, 6692.47) | -15.97% | 42.40 (26.99, 60.35) | 39.79 (25.86, 57.69) | -0.15 (-0.76, 0.47) |
| Ghana | 7637.02 (5748.93, 10068.23) | 13562.88 (10313.89, 17334.72) | 77.59% | 113.12 (85.16, 149.14) | 119.95 (91.22, 153.31) | 0.25 (0.07, 0.43) |
| Greece | 839.85 (501.30, 1239.62) | 632.48 (396.87, 923.33) | -24.69% | 41.52 (24.78, 61.28) | 43.27 (27.15, 63.17) | 0.32 (-0.63, 1.29) |
| Greenland | 12.15 (8.33, 16.94) | 9.20 (6.07, 12.84) | -24.29% | 85.48 (58.61, 119.20) | 78.91 (52.09, 110.11) | -0.06 (-1.21, 1.09) |
| Grenada | 38.00 (28.31, 49.53) | 26.94 (19.80, 35.31) | -29.10% | 118.54 (88.32, 154.51) | 120.73 (88.74, 158.24) | 0.13 (-0.13, 0.40) |
| Guam | 20.68 (13.62, 28.44) | 31.65 (22.08, 43.39) | 53.02% | 49.57 (32.65, 68.17) | 69.54 (48.52, 95.35) | 1.26 (1.07, 1.45) |
| Guatemala | 2286.74 (1636.34, 3074.95) | 4505.68 (3279.78, 5995.05) | 97.04% | 62.69 (44.86, 84.29) | 77.31 (56.28, 102.87) | 0.83 (0.15, 1.51) |
| Guinea | 1927.38 (1389.09, 2645.52) | 4896.15 (3609.58, 6346.37) | 154.03% | 67.94 (48.97, 93.26) | 85.70 (63.18, 111.08) | 0.87 (0.53, 1.20) |
| Guinea-Bissau | 512.67 (386.62, 657.63) | 919.04 (704.43, 1204.24) | 79.27% | 107.69 (81.21, 138.14) | 115.65 (88.64, 151.54) | 0.28 (0.14, 0.42) |
| Guyana | 367.47 (268.10, 497.39) | 258.04 (194.72, 337.15) | -29.78% | 129.11 (94.20, 174.76) | 123.65 (93.31, 161.56) | -0.09 (-0.32, 0.14) |
| Haiti | 2416.44 (1775.66, 3142.14) | 4319.91 (3163.08, 5748.38) | 78.77% | 89.80 (65.98, 116.76) | 101.02 (73.97, 134.42) | 0.54 (-0.08, 1.17) |
| Honduras | 1535.65 (1103.91, 2004.18) | 2779.62 (2053.81, 3572.33) | 81.01% | 69.59 (50.03, 90.83) | 84.25 (62.25, 108.28) | 0.69 (0.42, 0.97) |
| Hungary | 1536.26 (1063.89, 2086.08) | 816.57 (555.84, 1114.17) | -46.85% | 72.21 (50.01, 98.05) | 58.92 (40.11, 80.39) | -0.69 (-0.89, -0.50) |
| Iceland | 20.81 (12.91, 29.76) | 22.03 (13.44, 31.30) | 5.88% | 32.78 (20.34, 46.89) | 32.87 (20.05, 46.70) | 0.09 (-0.31, 0.48) |
| India | 206300.46 (145855.93, 289304.28) | 277586.34 (198445.20, 387426.57) | 34.55% | 62.31 (44.06, 87.38) | 73.80 (52.76, 103.00) | 0.68 (0.38, 0.99) |
| Indonesia | 54925.77 (39726.35, 74933.38) | 56248.74 (40757.21, 76007.28) | 2.41% | 80.64 (58.32, 110.01) | 86.39 (62.60, 116.74) | 0.27 (0.13, 0.40) |
| Iran (Islamic Republic of) | 16633.83 (11691.39, 23101.37) | 14410.88 (10259.40, 19714.47) | -13.36% | 64.19 (45.12, 89.15) | 70.65 (50.30, 96.65) | 0.34 (0.23, 0.45) |
| Iraq | 7751.08 (5742.53, 10104.10) | 13687.55 (10006.27, 17684.48) | 76.59% | 95.92 (71.06, 125.04) | 99.35 (72.63, 128.37) | 0.19 (-0.22, 0.60) |
| Ireland | 375.41 (228.01, 564.55) | 366.58 (215.25, 535.22) | -2.35% | 38.24 (23.23, 57.50) | 35.92 (21.09, 52.44) | -0.12 (-0.53, 0.30) |
| Israel | 522.82 (326.07, 763.02) | 847.03 (531.12, 1232.35) | 62.01% | 34.11 (21.28, 49.79) | 32.71 (20.51, 47.60) | -0.10 (-0.23, 0.02) |
| Italy | 2918.55 (1876.10, 4192.75) | 3110.38 (2109.70, 4342.82) | 6.57% | 31.62 (20.33, 45.43) | 39.12 (26.54, 54.62) | 0.77 (0.47, 1.06) |
| Jamaica | 789.01 (577.31, 1046.63) | 725.87 (541.24, 961.69) | -8.00% | 94.66 (69.26, 125.56) | 117.30 (87.46, 155.41) | 0.79 (0.49, 1.08) |
| Japan | 4160.46 (2684.78, 5903.61) | 5790.68 (3840.55, 8201.54) | 39.18% | 18.04 (11.64, 25.59) | 37.10 (24.61, 52.55) | 2.58 (2.36, 2.79) |
| Jordan | 1880.74 (1433.43, 2401.56) | 4130.67 (3046.52, 5381.67) | 119.63% | 113.05 (86.16, 144.35) | 110.93 (81.82, 144.53) | -0.03 (-0.22, 0.17) |
| Kazakhstan | 2218.68 (1476.95, 3084.77) | 2046.52 (1287.98, 2938.07) | -7.76% | 42.95 (28.59, 59.71) | 40.34 (25.39, 57.91) | -0.19 (-0.35, -0.03) |
| Kenya | 17801.44 (13162.57, 24285.58) | 33071.36 (24631.27, 45306.39) | 85.78% | 158.60 (117.27, 216.37) | 172.61 (128.56, 236.46) | 0.30 (0.26, 0.34) |
| Kiribati | 17.56 (12.17, 23.52) | 28.80 (20.03, 38.88) | 64.04% | 60.14 (41.69, 80.56) | 68.74 (47.82, 92.80) | 0.47 (-0.30, 1.25) |
| Kuwait | 418.19 (298.32, 553.58) | 703.93 (519.51, 935.25) | 68.33% | 73.97 (52.77, 97.92) | 81.72 (60.31, 108.58) | 0.36 (0.20, 0.51) |
| Kyrgyzstan | 506.70 (304.51, 749.99) | 619.25 (378.20, 931.40) | 22.21% | 30.41 (18.28, 45.01) | 29.66 (18.11, 44.61) | 0.14 (-1.08, 1.39) |
| Lao People's Democratic Republic | 849.04 (580.76, 1177.17) | 970.39 (624.34, 1358.79) | 14.29% | 46.52 (31.82, 64.50) | 43.13 (27.75, 60.39) | -0.17 (-0.68, 0.34) |
| Latvia | 205.14 (126.00, 294.01) | 143.24 (94.00, 204.06) | -30.17% | 36.06 (22.15, 51.68) | 47.15 (30.94, 67.17) | 0.94 (0.57, 1.32) |
| Lebanon | 911.53 (656.98, 1213.73) | 1129.90 (813.62, 1478.74) | 23.96% | 73.65 (53.08, 98.07) | 81.51 (58.69, 106.67) | 0.37 (0.22, 0.52) |
| Lesotho | 831.26 (607.84, 1089.02) | 781.57 (589.23, 1004.51) | -5.98% | 109.15 (79.81, 142.99) | 118.27 (89.17, 152.01) | 0.29 (0.18, 0.41) |
| Liberia | 956.36 (706.23, 1241.78) | 2171.68 (1588.85, 2812.39) | 127.08% | 105.61 (77.99, 137.13) | 116.25 (85.05, 150.55) | 0.35 (0.19, 0.51) |
| Libya | 1268.05 (880.42, 1729.28) | 1364.78 (969.55, 1785.41) | 7.63% | 67.35 (46.76, 91.84) | 92.93 (66.02, 121.57) | 1.21 (0.75, 1.67) |
| Lithuania | 460.06 (299.51, 649.67) | 218.57 (141.93, 310.52) | -52.49% | 55.50 (36.13, 78.37) | 52.56 (34.13, 74.67) | -0.17 (-0.29, -0.04) |
| Luxembourg | 21.40 (12.63, 33.07) | 34.81 (21.23, 51.24) | 62.64% | 32.42 (19.13, 50.09) | 35.23 (21.49, 51.86) | 0.33 (-0.22, 0.89) |
| Madagascar | 7917.80 (6082.54, 9997.68) | 17177.83 (13161.89, 21795.55) | 116.95% | 143.83 (110.49, 181.62) | 157.20 (120.45, 199.46) | 0.36 (0.10, 0.61) |
| Malawi | 6663.08 (5108.54, 8430.26) | 13506.55 (10353.25, 17137.55) | 102.71% | 150.41 (115.32, 190.30) | 171.45 (131.42, 217.54) | 0.56 (0.35, 0.76) |
| Malaysia | 5024.12 (3637.65, 6702.54) | 5823.39 (4188.31, 7696.16) | 15.91% | 76.38 (55.30, 101.90) | 75.68 (54.43, 100.02) | -0.06 (-0.42, 0.31) |
| Maldives | 45.24 (31.39, 62.31) | 41.90 (26.75, 59.73) | -7.37% | 43.22 (29.98, 59.52) | 38.08 (24.31, 54.28) | -0.39 (-0.59, -0.19) |
| Mali | 3691.12 (2724.41, 4808.74) | 9433.19 (6946.82, 12588.59) | 155.56% | 89.50 (66.06, 116.60) | 91.35 (67.27, 121.90) | 0.16 (-0.47, 0.80) |
| Malta | 28.23 (17.04, 41.73) | 19.78 (11.51, 30.09) | -29.92% | 32.25 (19.48, 47.68) | 31.42 (18.28, 47.79) | -0.04 (-0.44, 0.37) |
| Marshall Islands | 12.31 (8.53, 16.61) | 11.20 (7.72, 15.26) | -9.05% | 55.51 (38.46, 74.90) | 60.70 (41.85, 82.72) | 0.32 (0.11, 0.53) |
| Mauritania | 1110.29 (852.60, 1435.84) | 2060.67 (1546.42, 2634.27) | 85.60% | 118.67 (91.12, 153.46) | 127.78 (95.90, 163.35) | 0.29 (0.06, 0.53) |
| Mauritius | 267.66 (187.60, 355.61) | 164.93 (117.88, 218.28) | -38.38% | 81.03 (56.80, 107.66) | 76.75 (54.86, 101.57) | -0.01 (-0.24, 0.23) |
| Mexico | 33579.56 (24416.42, 46692.72) | 34708.23 (25421.38, 47826.40) | 3.36% | 100.19 (72.85, 139.32) | 107.49 (78.73, 148.12) | 0.29 (0.08, 0.49) |
| Micronesia (Federated States of) | 26.03 (17.39, 35.11) | 17.33 (12.03, 24.06) | -33.42% | 55.32 (36.96, 74.64) | 55.17 (38.30, 76.59) | 0.03 (-0.41, 0.48) |
| Monaco | 1.28 (0.81, 1.82) | 1.76 (1.08, 2.58) | 37.69% | 36.22 (22.85, 51.50) | 35.48 (21.73, 51.95) | 0.00 (-0.34, 0.33) |
| Mongolia | 245.66 (154.27, 352.67) | 286.55 (181.58, 406.66) | 16.65% | 27.36 (17.18, 39.27) | 28.31 (17.94, 40.17) | 0.07 (-0.46, 0.60) |
| Montenegro | 64.05 (42.52, 89.29) | 42.53 (28.46, 59.20) | -33.59% | 39.59 (26.29, 55.20) | 39.02 (26.11, 54.32) | -0.02 (-0.19, 0.16) |
| Morocco | 7938.94 (5727.61, 10370.42) | 8073.12 (5912.16, 10735.42) | 1.69% | 81.04 (58.47, 105.86) | 85.57 (62.66, 113.78) | 0.26 (0.02, 0.49) |
| Mozambique | 10447.68 (8163.06, 13211.84) | 25237.53 (19519.51, 31945.65) | 141.56% | 171.48 (133.98, 216.85) | 183.37 (141.82, 232.11) | 0.28 (0.06, 0.50) |
| Myanmar | 6586.37 (4318.82, 9289.05) | 6042.56 (3741.09, 8715.77) | -8.26% | 42.00 (27.54, 59.23) | 40.34 (24.98, 58.19) | -0.10 (-0.37, 0.17) |
| Namibia | 778.33 (589.12, 1003.83) | 1079.43 (802.79, 1381.38) | 38.69% | 130.80 (99.00, 168.70) | 128.88 (95.85, 164.93) | -0.03 (-0.13, 0.07) |
| Nauru | 2.71 (1.86, 3.68) | 2.69 (1.88, 3.66) | -0.43% | 60.91 (41.91, 82.78) | 68.80 (47.90, 93.36) | 0.50 (-0.14, 1.14) |
| Nepal | 3296.92 (2219.09, 4542.09) | 3753.07 (2340.58, 5305.76) | 13.84% | 38.79 (26.11, 53.43) | 40.71 (25.39, 57.55) | 0.23 (-0.54, 1.02) |
| Netherlands | 926.72 (575.67, 1337.31) | 1123.35 (698.11, 1649.20) | 21.22% | 34.01 (21.13, 49.08) | 41.09 (25.53, 60.32) | 0.78 (0.13, 1.44) |
| New Zealand | 216.82 (127.52, 329.35) | 418.36 (275.86, 598.93) | 92.95% | 27.10 (15.94, 41.17) | 46.89 (30.92, 67.12) | 1.96 (1.44, 2.50) |
| Nicaragua | 1660.64 (1222.69, 2192.87) | 1910.52 (1420.93, 2496.25) | 15.05% | 90.93 (66.95, 120.08) | 95.49 (71.02, 124.76) | 0.20 (-0.05, 0.44) |
| Niger | 3622.35 (2649.84, 4765.89) | 11134.39 (8270.32, 14492.12) | 207.38% | 89.31 (65.33, 117.51) | 92.61 (68.79, 120.54) | 0.22 (-0.25, 0.69) |
| Nigeria | 37398.03 (27466.20, 51895.11) | 106389.39 (78522.15, 145108.34) | 184.48% | 94.95 (69.74, 131.76) | 113.48 (83.76, 154.79) | 0.63 (0.49, 0.78) |
| Niue | 0.53 (0.37, 0.73) | 0.29 (0.21, 0.40) | -44.18% | 63.68 (44.14, 87.95) | 73.18 (50.89, 99.49) | 0.53 (0.16, 0.89) |
| North Macedonia | 248.03 (163.25, 357.61) | 169.26 (103.68, 249.63) | -31.76% | 46.27 (30.46, 66.72) | 48.99 (30.01, 72.25) | 0.65 (-1.06, 2.38) |
| Northern Mariana Islands | 6.71 (4.56, 9.12) | 5.60 (3.91, 7.68) | -16.60% | 54.05 (36.71, 73.43) | 75.76 (52.99, 103.96) | 1.25 (0.80, 1.70) |
| Norway | 414.64 (286.78, 588.28) | 566.15 (384.25, 791.45) | 36.54% | 51.88 (35.88, 73.61) | 60.52 (41.08, 84.61) | 0.58 (0.38, 0.79) |
| Oman | 578.39 (406.54, 778.66) | 835.70 (598.57, 1085.75) | 44.49% | 69.41 (48.78, 93.44) | 81.45 (58.34, 105.82) | 0.51 (0.22, 0.80) |
| Pakistan | 35567.16 (24912.69, 49417.63) | 68069.31 (47593.19, 97063.92) | 91.38% | 70.13 (49.12, 97.44) | 78.11 (54.61, 111.38) | 0.41 (0.24, 0.57) |
| Palau | 3.35 (2.30, 4.52) | 2.62 (1.84, 3.57) | -21.86% | 70.61 (48.55, 95.31) | 77.65 (54.53, 106.11) | 0.36 (0.17, 0.55) |
| Palestine | 689.01 (503.97, 909.07) | 1493.43 (1034.40, 1989.34) | 116.75% | 69.21 (50.63, 91.32) | 80.48 (55.74, 107.21) | 0.52 (0.27, 0.76) |
| Panama | 676.07 (486.00, 892.45) | 987.95 (740.01, 1311.40) | 46.13% | 81.08 (58.28, 107.03) | 86.38 (64.70, 114.66) | 0.28 (0.09, 0.48) |
| Papua New Guinea | 713.33 (478.22, 977.30) | 1554.09 (1038.19, 2142.40) | 117.86% | 42.50 (28.49, 58.23) | 42.22 (28.21, 58.21) | -0.01 (-0.12, 0.09) |
| Paraguay | 1453.97 (1095.72, 1867.82) | 1916.86 (1423.43, 2443.50) | 31.84% | 86.91 (65.49, 111.65) | 97.38 (72.32, 124.14) | 0.37 (0.24, 0.50) |
| Peru | 5103.43 (3588.47, 6839.43) | 5731.51 (4092.32, 7530.14) | 12.31% | 60.73 (42.70, 81.38) | 62.77 (44.81, 82.46) | 0.24 (0.10, 0.39) |
| Philippines | 11572.63 (8241.87, 15786.87) | 19496.75 (13861.62, 26842.92) | 68.47% | 45.52 (32.42, 62.09) | 54.82 (38.97, 75.47) | 0.70 (0.54, 0.87) |
| Poland | 4088.64 (2731.60, 5930.48) | 2591.65 (1779.34, 3675.42) | -36.61% | 42.83 (28.62, 62.13) | 44.01 (30.21, 62.41) | 0.16 (-0.28, 0.60) |
| Portugal | 1112.91 (700.14, 1632.61) | 674.77 (426.39, 1016.27) | -39.37% | 52.64 (33.11, 77.22) | 48.42 (30.60, 72.93) | -0.09 (-0.64, 0.47) |
| Puerto Rico | 1050.90 (766.48, 1376.67) | 601.70 (429.95, 796.19) | -42.74% | 105.50 (76.94, 138.20) | 114.98 (82.16, 152.14) | 0.36 (0.28, 0.44) |
| Qatar | 101.63 (73.34, 132.15) | 335.23 (244.29, 436.93) | 229.84% | 81.64 (58.91, 106.16) | 81.60 (59.47, 106.36) | -0.08 (-0.27, 0.11) |
| Republic of Korea | 5135.47 (3312.35, 7275.82) | 2140.85 (1270.09, 3233.28) | -58.31% | 44.91 (28.97, 63.63) | 31.22 (18.52, 47.15) | -1.20 (-1.54, -0.85) |
| Republic of Moldova | 386.35 (233.34, 553.42) | 222.52 (144.65, 319.33) | -42.40% | 31.37 (18.95, 44.93) | 38.54 (25.05, 55.31) | 0.76 (0.18, 1.33) |
| Romania | 2743.07 (1817.03, 3771.91) | 1535.08 (1004.46, 2203.45) | -44.04% | 49.19 (32.58, 67.64) | 50.67 (33.15, 72.72) | 0.23 (-0.63, 1.10) |
| Russian Federation | 12329.30 (8099.09, 17701.06) | 14789.65 (10420.06, 20764.63) | 19.96% | 35.51 (23.33, 50.98) | 54.79 (38.60, 76.92) | 1.59 (1.23, 1.95) |
| Rwanda | 4501.16 (3461.65, 5687.75) | 6477.17 (4964.34, 8274.20) | 43.90% | 133.10 (102.36, 168.18) | 133.73 (102.49, 170.83) | 0.10 (-0.20, 0.40) |
| Saint Kitts and Nevis | 17.12 (12.45, 23.37) | 14.70 (10.72, 19.38) | -14.14% | 122.08 (88.76, 166.63) | 124.45 (90.77, 164.03) | 0.12 (-0.35, 0.59) |
| Saint Lucia | 64.77 (46.78, 87.82) | 40.79 (29.89, 53.95) | -37.02% | 123.64 (89.30, 167.63) | 128.28 (94.00, 169.63) | 0.15 (0.04, 0.26) |
| Saint Vincent and the Grenadines | 43.47 (32.22, 58.34) | 28.29 (21.29, 36.69) | -34.92% | 104.36 (77.34, 140.04) | 113.15 (85.15, 146.76) | 0.35 (0.08, 0.62) |
| Samoa | 51.43 (36.29, 68.73) | 58.61 (41.10, 78.28) | 13.97% | 78.08 (55.09, 104.34) | 79.56 (55.79, 106.26) | 0.11 (-0.12, 0.34) |
| San Marino | 1.67 (1.01, 2.43) | 1.90 (1.17, 2.77) | 13.70% | 36.57 (22.19, 53.17) | 35.54 (21.84, 51.85) | -0.05 (-0.32, 0.22) |
| Sao Tome and Principe | 71.71 (54.18, 93.52) | 111.53 (85.04, 142.44) | 55.53% | 126.03 (95.23, 164.37) | 149.90 (114.30, 191.45) | 0.70 (0.35, 1.05) |
| Saudi Arabia | 8250.47 (6173.86, 10796.59) | 10623.68 (8013.25, 13608.86) | 28.76% | 123.66 (92.54, 161.83) | 150.77 (113.72, 193.13) | 1.03 (0.11, 1.97) |
| Senegal | 4127.97 (3107.67, 5268.07) | 7148.79 (5413.49, 8983.51) | 73.18% | 113.38 (85.36, 144.70) | 117.15 (88.72, 147.22) | 0.12 (-0.03, 0.27) |
| Serbia | 1095.02 (692.44, 1607.22) | 851.53 (540.61, 1235.02) | -22.24% | 51.89 (32.81, 76.17) | 58.18 (36.94, 84.39) | 0.62 (-0.24, 1.49) |
| Seychelles | 17.20 (12.24, 23.68) | 15.64 (11.20, 20.70) | -9.07% | 72.07 (51.29, 99.24) | 71.68 (51.32, 94.88) | 0.06 (-0.39, 0.52) |
| Sierra Leone | 1841.48 (1369.80, 2374.29) | 4219.76 (3187.66, 5488.92) | 129.15% | 115.38 (85.82, 148.76) | 126.29 (95.40, 164.27) | 0.38 (0.10, 0.65) |
| Singapore | 203.54 (120.56, 297.44) | 169.56 (96.64, 252.29) | -16.69% | 31.34 (18.56, 45.80) | 21.68 (12.36, 32.26) | -1.13 (-1.53, -0.73) |
| Slovakia | 703.79 (469.87, 972.48) | 293.16 (185.80, 419.95) | -58.35% | 53.12 (35.46, 73.39) | 34.49 (21.86, 49.41) | -1.49 (-1.89, -1.08) |
| Slovenia | 132.94 (78.88, 195.67) | 80.52 (47.59, 120.69) | -39.43% | 32.15 (19.08, 47.32) | 25.91 (15.32, 38.84) | -0.65 (-0.89, -0.41) |
| Solomon Islands | 83.37 (57.60, 113.82) | 129.69 (86.90, 180.68) | 55.56% | 53.04 (36.65, 72.42) | 50.21 (33.64, 69.95) | -0.13 (-0.53, 0.27) |
| Somalia | 4762.65 (3649.23, 6092.90) | 13525.07 (10492.91, 17154.53) | 183.98% | 137.32 (105.22, 175.68) | 142.37 (110.45, 180.57) | 0.16 (0.03, 0.29) |
| South Africa | 21566.19 (15864.99, 29887.60) | 23081.70 (17166.89, 31504.13) | 7.03% | 162.84 (119.79, 225.67) | 153.52 (114.18, 209.53) | -0.19 (-0.27, -0.11) |
| South Sudan | 3563.45 (2744.13, 4536.83) | 6365.24 (4750.74, 8037.96) | 78.63% | 136.60 (105.20, 173.92) | 152.70 (113.97, 192.83) | 0.51 (0.10, 0.92) |
| Spain | 3204.84 (1935.70, 4778.69) | 2519.51 (1604.95, 3679.19) | -21.38% | 40.91 (24.71, 60.99) | 37.18 (23.68, 54.29) | -0.32 (-0.66, 0.03) |
| Sri Lanka | 2566.43 (1657.37, 3582.75) | 2494.24 (1645.79, 3506.20) | -2.81% | 45.75 (29.54, 63.86) | 49.03 (32.35, 68.92) | 0.26 (0.09, 0.43) |
| Sudan | 6211.57 (4442.79, 8170.42) | 12827.17 (9299.86, 16870.58) | 106.50% | 68.42 (48.94, 90.00) | 81.18 (58.86, 106.77) | 0.68 (0.32, 1.04) |
| Suriname | 128.39 (94.47, 170.66) | 170.62 (128.70, 220.79) | 32.89% | 98.20 (72.26, 130.53) | 116.35 (87.76, 150.56) | 0.64 (0.35, 0.93) |
| Sweden | 1422.61 (926.92, 2021.96) | 2625.28 (1774.19, 3604.29) | 84.54% | 92.09 (60.00, 130.88) | 145.24 (98.16, 199.40) | 1.79 (1.11, 2.47) |
| Switzerland | 340.30 (204.24, 512.01) | 377.13 (224.23, 562.14) | 10.83% | 29.49 (17.70, 44.36) | 28.75 (17.09, 42.85) | 0.00 (-0.33, 0.33) |
| Syrian Arab Republic | 4438.34 (3236.35, 5740.40) | 3888.68 (2783.45, 5160.84) | -12.38% | 73.02 (53.24, 94.44) | 96.00 (68.71, 127.41) | 0.99 (0.81, 1.17) |
| Taiwan (Province of China) | 3377.88 (2315.99, 4639.05) | 1909.47 (1288.64, 2605.81) | -43.47% | 61.22 (41.98, 84.08) | 63.08 (42.57, 86.08) | 0.12 (-0.06, 0.31) |
| Tajikistan | 331.30 (160.12, 532.40) | 501.30 (262.64, 781.21) | 51.31% | 14.31 (6.91, 22.99) | 15.41 (8.07, 24.02) | 0.31 (0.13, 0.49) |
| Thailand | 7236.70 (4707.02, 10288.70) | 4498.36 (2862.86, 6359.62) | -37.84% | 42.55 (27.67, 60.49) | 42.19 (26.85, 59.64) | 0.02 (-0.11, 0.15) |
| Timor-Leste | 133.56 (89.70, 182.59) | 254.81 (170.62, 349.68) | 90.78% | 39.92 (26.81, 54.58) | 50.50 (33.81, 69.30) | 0.94 (-0.10, 1.98) |
| Togo | 1992.81 (1497.88, 2593.92) | 3802.98 (2810.36, 4981.20) | 90.84% | 112.02 (84.20, 145.81) | 120.82 (89.28, 158.25) | 0.29 (0.07, 0.51) |
| Tokelau | 0.39 (0.27, 0.53) | 0.30 (0.21, 0.40) | -24.19% | 55.83 (38.32, 75.05) | 63.67 (44.33, 85.49) | 0.43 (0.29, 0.56) |
| Tonga | 26.01 (17.54, 35.74) | 23.63 (16.37, 31.86) | -9.13% | 65.43 (44.14, 89.92) | 65.86 (45.61, 88.78) | 0.01 (-0.09, 0.12) |
| Trinidad and Tobago | 523.50 (396.08, 679.54) | 364.64 (272.17, 471.24) | -30.35% | 129.56 (98.02, 168.17) | 134.11 (100.10, 173.32) | 0.14 (0.06, 0.21) |
| Tunisia | 1235.30 (782.34, 1733.95) | 1442.37 (989.16, 1932.55) | 16.76% | 38.76 (24.55, 54.41) | 54.52 (37.39, 73.04) | 1.15 (0.62, 1.68) |
| Turkey | 16242.50 (11723.42, 21307.06) | 10835.99 (7939.54, 14638.84) | -33.29% | 75.15 (54.24, 98.59) | 68.71 (50.35, 92.83) | -0.30 (-0.48, -0.12) |
| Turkmenistan | 356.98 (210.67, 523.61) | 385.73 (234.98, 563.62) | 8.05% | 23.73 (14.01, 34.81) | 25.43 (15.49, 37.16) | 0.24 (-1.25, 1.74) |
| Tuvalu | 1.49 (1.02, 2.11) | 2.02 (1.38, 2.70) | 35.95% | 45.10 (31.00, 63.96) | 59.14 (40.34, 78.86) | 0.99 (0.49, 1.49) |
| Uganda | 11102.62 (8563.03, 14153.75) | 26147.51 (19850.88, 33138.21) | 135.51% | 131.61 (101.51, 167.78) | 137.06 (104.05, 173.70) | 0.18 (0.07, 0.29) |
| Ukraine | 7526.46 (5062.89, 10688.70) | 5914.59 (4053.77, 8234.82) | -21.42% | 66.38 (44.65, 94.27) | 85.63 (58.69, 119.22) | 0.88 (0.66, 1.10) |
| United Arab Emirates | 542.39 (400.61, 706.48) | 1347.71 (997.93, 1768.03) | 148.48% | 91.76 (67.77, 119.52) | 114.96 (85.13, 150.82) | 0.79 (0.68, 0.91) |
| United Kingdom | 4144.08 (2842.02, 5826.25) | 4888.89 (3354.17, 6748.89) | 17.97% | 37.83 (25.94, 53.19) | 41.26 (28.31, 56.95) | 0.34 (0.08, 0.59) |
| United Republic of Tanzania | 16144.93 (12242.35, 20632.23) | 37542.03 (28864.30, 48298.46) | 132.53% | 133.02 (100.86, 169.99) | 152.77 (117.46, 196.54) | 0.56 (0.29, 0.83) |
| United States of America | 33186.46 (22642.34, 46955.19) | 55894.46 (38846.74, 78229.45) | 68.43% | 59.64 (40.69, 84.39) | 92.92 (64.58, 130.04) | 1.74 (0.70, 2.79) |
| United States Virgin Islands | 29.51 (21.16, 38.25) | 19.35 (14.01, 25.38) | -34.41% | 92.69 (66.47, 120.15) | 96.65 (69.96, 126.77) | 0.15 (0.08, 0.22) |
| Uruguay | 265.78 (163.04, 386.86) | 241.49 (152.28, 352.84) | -9.14% | 32.48 (19.92, 47.27) | 34.43 (21.71, 50.30) | 0.24 (-0.12, 0.60) |
| Uzbekistan | 2095.34 (1285.13, 3095.72) | 3386.78 (2006.34, 4978.52) | 61.63% | 24.46 (15.00, 36.13) | 32.89 (19.49, 48.35) | 1.09 (0.50, 1.67) |
| Vanuatu | 52.12 (37.72, 69.20) | 87.18 (63.26, 118.42) | 67.28% | 77.90 (56.38, 103.43) | 78.66 (57.07, 106.84) | 0.10 (-0.11, 0.31) |
| Venezuela (Bolivarian Republic of) | 7476.21 (5666.22, 9724.24) | 7005.43 (4993.74, 9036.31) | -6.30% | 105.16 (79.70, 136.78) | 99.49 (70.92, 128.33) | -0.15 (-0.29, -0.01) |
| Viet Nam | 10359.06 (6765.11, 14839.09) | 11201.50 (7571.83, 15171.14) | 8.13% | 39.54 (25.82, 56.65) | 52.54 (35.51, 71.15) | 1.16 (0.46, 1.87) |
| Yemen | 4765.74 (3407.79, 6406.40) | 9952.01 (7130.50, 12926.91) | 108.82% | 66.36 (47.45, 89.21) | 76.87 (55.07, 99.84) | 0.55 (0.17, 0.93) |
| Zambia | 5009.10 (3864.67, 6474.25) | 11404.03 (8454.27, 14500.61) | 127.67% | 133.15 (102.73, 172.10) | 147.46 (109.32, 187.50) | 0.39 (0.15, 0.63) |
| Zimbabwe | 5483.65 (4018.37, 7058.17) | 7161.32 (5335.92, 9209.32) | 30.59% | 113.81 (83.40, 146.49) | 120.03 (89.44, 154.36) | 0.21 (0.09, 0.33) |

Note: ASR: age-standardized rates; AAPC, average annual percentage change; CI, confidence interval; UI, uncertainty interval

Table S5**. The deaths and age-standardized mortality rate for stroke and their temporal change among children (0-14 years) in 204 countries or territories from 1990 to 2019.**

|  | Deaths (95% UI) | |  | Age-standardized mortality rate (95% UI), per 100,000 population | | |
| --- | --- | --- | --- | --- | --- | --- |
| **Countries or territories** | 1990 | 2019 | Percentage change (%) | 1990 | 2019 | AAPC (95% CI) |
| Afghanistan | 119.80 (70.67, 201.95) | 113.67 (72.34, 203.82) | -5.12% | 2.40 (1.42, 4.05) | 0.66 (0.42, 1.18) | -4.26 (-5.06, -3.46) |
| Albania | 34.40 (26.97, 44.73) | 13.54 (7.53, 19.27) | -60.63% | 3.06 (2.40, 3.98) | 2.86 (1.59, 4.07) | -0.30 (-1.08, 0.49) |
| Algeria | 330.03 (199.79, 568.87) | 79.63 (53.56, 113.24) | -75.87% | 3.08 (1.86, 5.31) | 0.68 (0.46, 0.96) | -5.04 (-5.35, -4.73) |
| American Samoa | 0.16 (0.12, 0.22) | 0.07 (0.05, 0.11) | -54.67% | 0.87 (0.62, 1.18) | 0.45 (0.30, 0.65) | -2.20 (-2.62, -1.77) |
| Andorra | 0.03 (0.01, 0.04) | 0.00 (0.00, 0.01) | -83.60% | 0.29 (0.15, 0.45) | 0.04 (0.03, 0.06) | -6.43 (-6.91, -5.95) |
| Angola | 179.58 (83.37, 367.08) | 118.29 (72.00, 181.20) | -34.13% | 3.78 (1.76, 7.73) | 0.85 (0.51, 1.30) | -5.09 (-5.44, -4.73) |
| Antigua and Barbuda | 0.08 (0.06, 0.09) | 0.04 (0.03, 0.05) | -53.49% | 0.40 (0.32, 0.50) | 0.21 (0.15, 0.28) | -2.21 (-2.77, -1.64) |
| Argentina | 145.47 (121.65, 172.33) | 29.98 (23.63, 36.93) | -79.39% | 1.44 (1.20, 1.70) | 0.28 (0.22, 0.35) | -5.55 (-6.07, -5.03) |
| Armenia | 1.99 (1.46, 2.57) | 0.30 (0.18, 0.42) | -84.97% | 0.19 (0.14, 0.25) | 0.05 (0.03, 0.07) | -4.38 (-5.11, -3.65) |
| Australia | 9.15 (7.51, 11.12) | 2.18 (1.60, 2.96) | -76.19% | 0.24 (0.20, 0.29) | 0.05 (0.03, 0.06) | -5.32 (-5.85, -4.78) |
| Austria | 5.04 (3.93, 5.95) | 0.85 (0.52, 1.14) | -83.10% | 0.37 (0.29, 0.44) | 0.07 (0.04, 0.09) | -5.74 (-6.26, -5.21) |
| Azerbaijan | 4.61 (3.10, 7.21) | 2.29 (1.43, 3.35) | -50.37% | 0.19 (0.13, 0.30) | 0.10 (0.06, 0.14) | -2.38 (-3.19, -1.57) |
| Bahamas | 0.39 (0.30, 0.51) | 0.13 (0.09, 0.17) | -67.75% | 0.49 (0.37, 0.63) | 0.16 (0.12, 0.21) | -3.88 (-4.92, -2.83) |
| Bahrain | 3.49 (2.30, 4.98) | 0.65 (0.48, 0.88) | -81.33% | 2.13 (1.40, 3.03) | 0.28 (0.21, 0.37) | -6.80 (-7.73, -5.86) |
| Bangladesh | 1213.76 (716.64, 1699.53) | 390.97 (271.52, 531.73) | -67.79% | 2.51 (1.48, 3.51) | 0.89 (0.62, 1.21) | -3.42 (-3.66, -3.18) |
| Barbados | 0.27 (0.21, 0.34) | 0.08 (0.06, 0.11) | -70.35% | 0.43 (0.34, 0.54) | 0.16 (0.11, 0.23) | -3.13 (-4.03, -2.22) |
| Belarus | 2.15 (1.58, 2.91) | 0.31 (0.18, 0.52) | -85.83% | 0.09 (0.07, 0.12) | 0.02 (0.01, 0.03) | -5.67 (-6.75, -4.59) |
| Belgium | 11.05 (9.39, 12.77) | 2.01 (1.45, 2.57) | -81.84% | 0.61 (0.52, 0.71) | 0.10 (0.07, 0.13) | -6.07 (-6.44, -5.69) |
| Belize | 0.85 (0.65, 1.11) | 0.26 (0.20, 0.34) | -69.39% | 1.06 (0.81, 1.37) | 0.21 (0.16, 0.28) | -5.59 (-6.58, -4.60) |
| Benin | 90.83 (57.07, 136.04) | 130.19 (83.32, 192.74) | 43.34% | 3.75 (2.35, 5.61) | 2.26 (1.45, 3.35) | -1.70 (-1.98, -1.43) |
| Bermuda | 0.03 (0.02, 0.03) | 0.00 (0.00, 0.01) | -85.54% | 0.22 (0.16, 0.29) | 0.04 (0.03, 0.06) | -5.46 (-5.94, -4.99) |
| Bhutan | 2.91 (0.89, 5.57) | 0.73 (0.46, 1.09) | -74.85% | 1.15 (0.35, 2.20) | 0.38 (0.23, 0.56) | -3.76 (-4.24, -3.27) |
| Bolivia (Plurinational State of) | 95.53 (59.34, 147.43) | 31.70 (21.97, 44.25) | -66.82% | 3.50 (2.17, 5.40) | 0.81 (0.56, 1.13) | -4.94 (-5.07, -4.80) |
| Bosnia and Herzegovina | 10.99 (8.78, 13.41) | 2.06 (1.52, 2.67) | -81.24% | 0.99 (0.79, 1.21) | 0.43 (0.32, 0.56) | -2.95 (-5.13, -0.72) |
| Botswana | 3.29 (2.04, 4.85) | 4.97 (2.46, 8.87) | 50.91% | 0.58 (0.36, 0.85) | 0.71 (0.35, 1.26) | 0.74 (0.02, 1.47) |
| Brazil | 661.77 (540.08, 819.58) | 187.45 (153.41, 225.02) | -71.67% | 1.27 (1.03, 1.57) | 0.39 (0.32, 0.47) | -3.93 (-4.22, -3.65) |
| Brunei Darussalam | 0.61 (0.44, 0.80) | 0.15 (0.11, 0.24) | -74.93% | 0.68 (0.48, 0.88) | 0.16 (0.11, 0.25) | -4.77 (-5.33, -4.21) |
| Bulgaria | 25.24 (21.73, 29.79) | 4.06 (2.74, 6.29) | -83.93% | 1.45 (1.25, 1.72) | 0.41 (0.28, 0.64) | -4.70 (-5.34, -4.06) |
| Burkina Faso | 159.78 (103.75, 240.92) | 315.81 (197.18, 530.44) | 97.65% | 3.38 (2.19, 5.10) | 3.04 (1.90, 5.10) | -0.26 (-0.52, 0.00) |
| Burundi | 60.80 (33.53, 99.24) | 41.01 (25.01, 71.44) | -32.55% | 2.30 (1.27, 3.76) | 0.76 (0.46, 1.32) | -3.81 (-4.52, -3.09) |
| Cabo Verde | 3.75 (2.47, 5.81) | 1.05 (0.68, 1.55) | -71.93% | 2.42 (1.59, 3.75) | 0.66 (0.42, 0.98) | -4.22 (-4.93, -3.50) |
| Cambodia | 222.69 (86.62, 420.00) | 55.69 (24.45, 94.53) | -74.99% | 4.67 (1.82, 8.81) | 1.11 (0.49, 1.88) | -4.83 (-4.99, -4.66) |
| Cameroon | 122.92 (76.40, 183.11) | 249.46 (157.73, 399.97) | 102.95% | 2.54 (1.58, 3.78) | 2.07 (1.31, 3.32) | -0.66 (-1.19, -0.13) |
| Canada | 19.22 (15.89, 23.55) | 5.61 (4.11, 8.02) | -70.82% | 0.33 (0.28, 0.41) | 0.09 (0.07, 0.13) | -4.29 (-5.04, -3.53) |
| Central African Republic | 35.14 (17.16, 63.00) | 42.85 (23.64, 71.74) | 21.92% | 2.87 (1.40, 5.14) | 1.92 (1.06, 3.21) | -1.29 (-1.56, -1.03) |
| Chad | 141.12 (89.77, 207.80) | 285.66 (195.92, 402.95) | 102.42% | 4.83 (3.07, 7.11) | 3.44 (2.36, 4.85) | -1.07 (-1.46, -0.68) |
| Chile | 18.14 (15.09, 21.24) | 4.68 (3.47, 6.10) | -74.18% | 0.46 (0.38, 0.53) | 0.13 (0.10, 0.17) | -3.98 (-4.14, -3.82) |
| China | 10933.49 (7462.62, 13403.35) | 689.87 (545.84, 961.93) | -93.69% | 3.39 (2.31, 4.15) | 0.31 (0.24, 0.43) | -8.01 (-8.54, -7.48) |
| Colombia | 153.89 (133.18, 176.14) | 50.71 (35.32, 69.03) | -67.05% | 1.31 (1.14, 1.50) | 0.46 (0.32, 0.63) | -3.60 (-4.14, -3.05) |
| Comoros | 3.17 (1.32, 4.89) | 1.24 (0.71, 1.96) | -60.98% | 1.49 (0.62, 2.30) | 0.53 (0.30, 0.84) | -3.57 (-5.35, -1.75) |
| Congo | 17.92 (10.11, 28.71) | 9.97 (6.26, 16.40) | -44.37% | 1.63 (0.92, 2.62) | 0.50 (0.31, 0.82) | -3.91 (-4.10, -3.71) |
| Cook Islands | 0.10 (0.07, 0.15) | 0.01 (0.00, 0.01) | -91.87% | 1.52 (1.01, 2.25) | 0.20 (0.11, 0.31) | -6.80 (-7.51, -6.09) |
| Costa Rica | 3.75 (3.01, 4.64) | 1.08 (0.74, 1.59) | -71.07% | 0.33 (0.27, 0.41) | 0.10 (0.07, 0.15) | -3.81 (-4.31, -3.30) |
| Croatia | 6.67 (5.70, 7.82) | 0.86 (0.60, 1.17) | -87.11% | 0.68 (0.58, 0.79) | 0.14 (0.10, 0.19) | -5.31 (-6.53, -4.07) |
| Cuba | 8.18 (6.53, 10.13) | 1.97 (1.36, 2.88) | -75.94% | 0.33 (0.26, 0.41) | 0.11 (0.08, 0.16) | -3.52 (-4.02, -3.02) |
| Cyprus | 1.75 (1.08, 2.67) | 0.30 (0.20, 0.40) | -82.87% | 0.88 (0.55, 1.35) | 0.14 (0.09, 0.19) | -5.83 (-6.89, -4.76) |
| Czechia | 10.15 (7.96, 14.48) | 0.60 (0.38, 1.16) | -94.13% | 0.46 (0.36, 0.66) | 0.03 (0.02, 0.07) | -8.58 (-9.55, -7.59) |
| Cote d'Ivoire | 234.20 (161.27, 326.73) | 226.71 (140.80, 338.51) | -3.20% | 4.09 (2.81, 5.70) | 2.12 (1.32, 3.17) | -2.24 (-2.53, -1.94) |
| Democratic People's Republic of Korea | 244.26 (136.38, 406.45) | 24.39 (14.66, 38.11) | -90.01% | 3.75 (2.09, 6.24) | 0.50 (0.30, 0.78) | -6.72 (-6.95, -6.48) |
| Democratic Republic of the Congo | 535.19 (266.31, 888.48) | 264.28 (162.92, 420.00) | -50.62% | 2.95 (1.47, 4.90) | 0.70 (0.43, 1.11) | -4.89 (-5.05, -4.72) |
| Denmark | 2.34 (1.74, 2.93) | 0.27 (0.17, 0.40) | -88.30% | 0.26 (0.20, 0.33) | 0.03 (0.02, 0.04) | -7.36 (-8.14, -6.57) |
| Djibouti | 2.58 (1.59, 4.06) | 2.52 (1.42, 4.25) | -2.49% | 1.18 (0.73, 1.85) | 0.60 (0.34, 1.01) | -2.30 (-2.93, -1.67) |
| Dominica | 0.07 (0.05, 0.09) | 0.04 (0.02, 0.05) | -47.28% | 0.28 (0.21, 0.36) | 0.25 (0.16, 0.37) | -0.38 (-1.08, 0.33) |
| Dominican Republic | 56.58 (41.45, 76.10) | 21.48 (14.26, 31.96) | -62.04% | 2.06 (1.51, 2.77) | 0.71 (0.47, 1.05) | -3.71 (-4.15, -3.27) |
| Ecuador | 38.02 (31.90, 45.99) | 23.28 (17.48, 30.75) | -38.76% | 0.97 (0.82, 1.18) | 0.46 (0.35, 0.61) | -2.62 (-3.21, -2.03) |
| Egypt | 9950.18 (4439.35, 13877.53) | 2035.99 (1109.01, 3227.94) | -79.54% | 44.70 (19.94, 62.35) | 6.23 (3.40, 9.88) | -6.58 (-6.94, -6.22) |
| El Salvador | 77.47 (58.71, 93.16) | 7.65 (5.37, 10.74) | -90.13% | 3.65 (2.76, 4.38) | 0.45 (0.31, 0.63) | -6.98 (-7.65, -6.32) |
| Equatorial Guinea | 5.44 (2.75, 9.10) | 1.79 (0.92, 3.03) | -67.07% | 2.65 (1.34, 4.43) | 0.32 (0.16, 0.54) | -7.09 (-8.05, -6.12) |
| Eritrea | 20.48 (11.28, 33.88) | 12.93 (7.93, 22.47) | -36.85% | 1.45 (0.80, 2.41) | 0.49 (0.30, 0.85) | -3.72 (-4.11, -3.34) |
| Estonia | 0.89 (0.73, 1.08) | 0.04 (0.03, 0.05) | -95.51% | 0.25 (0.21, 0.31) | 0.02 (0.01, 0.03) | -8.64 (-9.63, -7.64) |
| Eswatini | 3.97 (2.06, 6.46) | 2.62 (1.72, 3.75) | -34.11% | 1.03 (0.53, 1.68) | 0.64 (0.42, 0.92) | -1.69 (-1.97, -1.40) |
| Ethiopia | 572.03 (300.33, 916.74) | 221.62 (136.07, 341.38) | -61.26% | 2.31 (1.21, 3.70) | 0.48 (0.30, 0.74) | -5.19 (-5.62, -4.75) |
| Fiji | 4.28 (3.06, 5.66) | 4.24 (2.98, 5.99) | -1.05% | 1.52 (1.09, 2.01) | 1.60 (1.12, 2.26) | 0.16 (-0.35, 0.68) |
| Finland | 3.53 (2.65, 4.39) | 0.54 (0.36, 0.85) | -84.56% | 0.37 (0.27, 0.45) | 0.06 (0.04, 0.10) | -5.85 (-7.33, -4.35) |
| France | 48.78 (42.76, 55.86) | 12.44 (9.14, 15.78) | -74.50% | 0.42 (0.37, 0.48) | 0.11 (0.08, 0.13) | -4.67 (-5.37, -3.96) |
| Gabon | 5.44 (3.03, 8.40) | 1.99 (1.23, 3.17) | -63.34% | 1.30 (0.72, 2.01) | 0.34 (0.21, 0.54) | -4.56 (-4.85, -4.27) |
| Gambia | 13.00 (8.37, 18.71) | 10.98 (6.38, 21.04) | -15.53% | 2.76 (1.78, 3.97) | 1.19 (0.69, 2.27) | -2.88 (-3.90, -1.86) |
| Georgia | 7.52 (5.85, 9.51) | 1.07 (0.77, 1.43) | -85.77% | 0.55 (0.43, 0.70) | 0.15 (0.11, 0.20) | -4.53 (-5.48, -3.56) |
| Germany | 59.05 (48.15, 69.81) | 8.39 (6.05, 10.81) | -85.78% | 0.46 (0.37, 0.54) | 0.07 (0.05, 0.09) | -6.02 (-6.41, -5.61) |
| Ghana | 137.68 (96.20, 194.11) | 123.33 (79.04, 184.98) | -10.43% | 2.04 (1.43, 2.88) | 1.09 (0.70, 1.64) | -2.13 (-2.50, -1.75) |
| Greece | 9.18 (7.71, 11.09) | 1.85 (1.40, 2.33) | -79.80% | 0.45 (0.38, 0.55) | 0.13 (0.10, 0.16) | -4.30 (-5.12, -3.48) |
| Greenland | 0.45 (0.30, 0.64) | 0.04 (0.02, 0.06) | -90.83% | 3.15 (2.10, 4.49) | 0.35 (0.20, 0.55) | -7.36 (-8.00, -6.72) |
| Grenada | 0.23 (0.17, 0.29) | 0.05 (0.04, 0.07) | -76.21% | 0.71 (0.54, 0.90) | 0.24 (0.17, 0.32) | -3.58 (-3.89, -3.28) |
| Guam | 0.42 (0.33, 0.55) | 0.44 (0.33, 0.58) | 4.66% | 1.01 (0.79, 1.31) | 0.97 (0.72, 1.28) | -0.19 (-0.99, 0.63) |
| Guatemala | 69.43 (54.86, 83.77) | 41.88 (29.18, 60.11) | -39.68% | 1.90 (1.50, 2.30) | 0.72 (0.50, 1.03) | -3.23 (-3.63, -2.82) |
| Guinea | 180.01 (108.97, 294.12) | 197.45 (123.56, 303.93) | 9.69% | 6.35 (3.84, 10.37) | 3.46 (2.16, 5.32) | -2.05 (-2.48, -1.63) |
| Guinea-Bissau | 25.81 (16.27, 39.74) | 17.18 (11.43, 26.10) | -33.43% | 5.42 (3.42, 8.35) | 2.16 (1.44, 3.28) | -3.05 (-3.43, -2.67) |
| Guyana | 7.07 (5.66, 8.58) | 1.72 (1.20, 2.38) | -75.62% | 2.48 (1.99, 3.01) | 0.83 (0.57, 1.14) | -3.76 (-4.76, -2.76) |
| Haiti | 287.83 (134.93, 455.76) | 202.70 (113.13, 327.04) | -29.57% | 10.70 (5.01, 16.94) | 4.74 (2.65, 7.65) | -2.75 (-2.94, -2.56) |
| Honduras | 108.54 (74.00, 143.51) | 35.97 (19.73, 57.99) | -66.86% | 4.92 (3.35, 6.50) | 1.09 (0.60, 1.76) | -5.05 (-5.37, -4.74) |
| Hungary | 9.89 (8.38, 11.49) | 1.48 (0.94, 2.03) | -85.06% | 0.46 (0.39, 0.54) | 0.11 (0.07, 0.15) | -4.66 (-5.59, -3.72) |
| Iceland | 0.36 (0.29, 0.43) | 0.06 (0.03, 0.08) | -84.56% | 0.57 (0.46, 0.68) | 0.08 (0.05, 0.12) | -6.38 (-7.05, -5.70) |
| India | 3380.39 (2496.35, 4523.02) | 1253.97 (1004.67, 1590.72) | -62.90% | 1.02 (0.75, 1.37) | 0.33 (0.27, 0.42) | -3.81 (-4.49, -3.12) |
| Indonesia | 1694.67 (991.75, 2517.68) | 398.99 (314.75, 515.11) | -76.46% | 2.49 (1.46, 3.70) | 0.61 (0.48, 0.79) | -4.68 (-4.80, -4.56) |
| Iran (Islamic Republic of) | 528.32 (384.06, 762.42) | 91.05 (64.29, 116.23) | -82.77% | 2.04 (1.48, 2.94) | 0.45 (0.32, 0.57) | -5.11 (-5.53, -4.69) |
| Iraq | 334.82 (214.36, 487.46) | 143.40 (99.55, 204.94) | -57.17% | 4.14 (2.65, 6.03) | 1.04 (0.72, 1.49) | -4.57 (-5.38, -3.76) |
| Ireland | 2.34 (1.88, 2.82) | 0.38 (0.24, 0.52) | -83.63% | 0.24 (0.19, 0.29) | 0.04 (0.02, 0.05) | -6.08 (-7.12, -5.02) |
| Israel | 7.38 (5.86, 9.29) | 1.58 (1.16, 2.08) | -78.63% | 0.48 (0.38, 0.61) | 0.06 (0.04, 0.08) | -6.92 (-7.74, -6.09) |
| Italy | 60.01 (55.27, 69.04) | 10.05 (7.99, 11.40) | -83.26% | 0.65 (0.60, 0.75) | 0.13 (0.10, 0.14) | -5.48 (-6.41, -4.54) |
| Jamaica | 21.74 (17.21, 27.07) | 3.67 (2.62, 5.04) | -83.11% | 2.61 (2.06, 3.25) | 0.59 (0.42, 0.81) | -5.06 (-5.98, -4.14) |
| Japan | 91.89 (78.91, 99.15) | 15.74 (12.23, 18.21) | -82.87% | 0.40 (0.34, 0.43) | 0.10 (0.08, 0.12) | -4.65 (-5.23, -4.06) |
| Jordan | 9.87 (7.02, 14.85) | 7.09 (4.73, 11.48) | -28.20% | 0.59 (0.42, 0.89) | 0.19 (0.13, 0.31) | -3.85 (-4.52, -3.18) |
| Kazakhstan | 23.27 (17.08, 28.89) | 9.95 (7.10, 15.38) | -57.22% | 0.45 (0.33, 0.56) | 0.20 (0.14, 0.30) | -2.97 (-3.60, -2.34) |
| Kenya | 91.69 (57.02, 119.15) | 60.02 (41.02, 86.77) | -34.53% | 0.82 (0.51, 1.06) | 0.31 (0.21, 0.45) | -3.28 (-3.65, -2.90) |
| Kiribati | 1.38 (0.94, 1.90) | 0.81 (0.52, 1.20) | -40.97% | 4.71 (3.23, 6.51) | 1.94 (1.24, 2.87) | -3.07 (-3.50, -2.65) |
| Kuwait | 5.04 (3.96, 6.72) | 3.56 (2.70, 4.68) | -29.33% | 0.89 (0.70, 1.19) | 0.41 (0.31, 0.54) | -2.34 (-3.57, -1.09) |
| Kyrgyzstan | 3.40 (2.63, 4.33) | 1.31 (0.98, 1.65) | -61.58% | 0.20 (0.16, 0.26) | 0.06 (0.05, 0.08) | -4.07 (-5.33, -2.79) |
| Lao People's Democratic Republic | 67.04 (35.17, 111.98) | 34.59 (21.11, 52.90) | -48.41% | 3.67 (1.93, 6.14) | 1.54 (0.94, 2.35) | -2.92 (-3.15, -2.69) |
| Latvia | 1.23 (0.83, 1.65) | 0.10 (0.06, 0.14) | -92.06% | 0.22 (0.15, 0.29) | 0.03 (0.02, 0.05) | -6.33 (-7.70, -4.94) |
| Lebanon | 7.28 (4.21, 11.14) | 1.58 (0.73, 3.19) | -78.35% | 0.59 (0.34, 0.90) | 0.11 (0.05, 0.23) | -5.50 (-5.88, -5.13) |
| Lesotho | 5.13 (3.10, 7.99) | 4.27 (2.73, 6.48) | -16.78% | 0.67 (0.41, 1.05) | 0.65 (0.41, 0.98) | -0.27 (-0.94, 0.41) |
| Liberia | 67.66 (40.82, 112.54) | 28.53 (18.31, 44.01) | -57.83% | 7.47 (4.51, 12.43) | 1.53 (0.98, 2.36) | -5.51 (-5.89, -5.14) |
| Libya | 229.16 (106.99, 346.50) | 32.97 (18.65, 50.54) | -85.61% | 12.17 (5.68, 18.40) | 2.24 (1.27, 3.44) | -5.69 (-6.04, -5.34) |
| Lithuania | 1.05 (0.82, 1.40) | 0.12 (0.09, 0.18) | -88.31% | 0.13 (0.10, 0.17) | 0.03 (0.02, 0.04) | -5.09 (-7.46, -2.65) |
| Luxembourg | 0.45 (0.37, 0.54) | 0.08 (0.05, 0.11) | -83.00% | 0.68 (0.56, 0.82) | 0.08 (0.05, 0.11) | -7.29 (-8.37, -6.21) |
| Madagascar | 193.95 (123.93, 289.54) | 110.66 (60.01, 218.11) | -42.95% | 3.52 (2.25, 5.26) | 1.01 (0.55, 2.00) | -4.11 (-4.48, -3.74) |
| Malawi | 126.73 (66.87, 225.61) | 47.54 (29.55, 73.82) | -62.48% | 2.86 (1.51, 5.09) | 0.60 (0.38, 0.94) | -5.31 (-6.40, -4.20) |
| Malaysia | 98.87 (61.64, 135.15) | 32.16 (21.94, 44.24) | -67.47% | 1.50 (0.94, 2.05) | 0.42 (0.29, 0.57) | -3.84 (-4.56, -3.11) |
| Maldives | 6.29 (2.92, 10.12) | 0.66 (0.36, 1.23) | -89.49% | 6.01 (2.79, 9.67) | 0.60 (0.33, 1.12) | -7.52 (-8.11, -6.92) |
| Mali | 242.43 (153.41, 391.12) | 408.00 (242.42, 777.91) | 68.29% | 5.88 (3.72, 9.48) | 3.95 (2.35, 7.53) | -1.38 (-2.25, -0.50) |
| Malta | 0.53 (0.42, 0.65) | 0.12 (0.08, 0.16) | -78.06% | 0.60 (0.49, 0.74) | 0.18 (0.13, 0.25) | -4.05 (-4.52, -3.59) |
| Marshall Islands | 0.30 (0.19, 0.44) | 0.22 (0.14, 0.33) | -25.89% | 1.36 (0.87, 1.99) | 1.21 (0.78, 1.78) | -0.46 (-0.71, -0.22) |
| Mauritania | 22.62 (16.10, 31.43) | 14.68 (8.70, 23.62) | -35.09% | 2.42 (1.72, 3.36) | 0.91 (0.54, 1.47) | -3.31 (-3.77, -2.84) |
| Mauritius | 2.43 (2.00, 2.90) | 0.41 (0.30, 0.55) | -83.03% | 0.73 (0.61, 0.88) | 0.19 (0.14, 0.25) | -4.39 (-5.98, -2.77) |
| Mexico | 411.52 (351.74, 470.29) | 170.03 (125.65, 266.43) | -58.68% | 1.23 (1.05, 1.40) | 0.53 (0.39, 0.83) | -2.78 (-2.99, -2.56) |
| Micronesia (Federated States of) | 0.94 (0.60, 1.39) | 0.25 (0.10, 0.39) | -72.89% | 1.99 (1.27, 2.95) | 0.81 (0.31, 1.24) | -3.05 (-3.32, -2.77) |
| Monaco | 0.02 (0.01, 0.03) | 0.00 (0.00, 0.01) | -71.90% | 0.44 (0.26, 0.72) | 0.09 (0.05, 0.13) | -5.43 (-5.59, -5.27) |
| Mongolia | 42.87 (20.86, 59.42) | 7.14 (4.13, 10.07) | -83.34% | 4.77 (2.32, 6.62) | 0.71 (0.41, 0.99) | -6.28 (-6.89, -5.67) |
| Montenegro | 1.09 (0.83, 1.36) | 0.14 (0.10, 0.22) | -87.06% | 0.67 (0.51, 0.84) | 0.13 (0.09, 0.20) | -5.25 (-7.02, -3.45) |
| Morocco | 311.33 (201.61, 454.96) | 70.32 (36.44, 110.68) | -77.41% | 3.18 (2.06, 4.64) | 0.75 (0.39, 1.17) | -4.74 (-5.04, -4.43) |
| Mozambique | 105.92 (57.99, 183.98) | 69.91 (38.92, 120.20) | -34.00% | 1.74 (0.95, 3.02) | 0.51 (0.28, 0.87) | -4.30 (-4.97, -3.63) |
| Myanmar | 1355.35 (572.60, 2879.27) | 474.27 (199.04, 775.66) | -65.01% | 8.64 (3.65, 18.36) | 3.17 (1.33, 5.18) | -3.40 (-3.65, -3.16) |
| Namibia | 4.35 (1.96, 7.06) | 2.93 (1.68, 4.47) | -32.58% | 0.73 (0.33, 1.19) | 0.35 (0.20, 0.53) | -2.70 (-3.46, -1.94) |
| Nauru | 0.17 (0.11, 0.25) | 0.12 (0.08, 0.18) | -28.99% | 3.76 (2.47, 5.67) | 3.03 (1.93, 4.50) | -0.77 (-1.10, -0.45) |
| Nepal | 87.97 (51.79, 136.27) | 20.23 (12.21, 29.69) | -77.00% | 1.03 (0.61, 1.60) | 0.22 (0.13, 0.32) | -5.24 (-5.51, -4.96) |
| Netherlands | 10.06 (8.12, 12.29) | 2.19 (1.42, 2.84) | -78.19% | 0.37 (0.30, 0.45) | 0.08 (0.05, 0.10) | -5.09 (-5.75, -4.43) |
| New Zealand | 2.59 (2.19, 3.13) | 0.61 (0.43, 0.78) | -76.34% | 0.32 (0.27, 0.39) | 0.07 (0.05, 0.09) | -5.32 (-6.43, -4.21) |
| Nicaragua | 19.80 (13.87, 27.16) | 4.26 (2.95, 6.51) | -78.51% | 1.08 (0.76, 1.49) | 0.21 (0.15, 0.33) | -5.47 (-5.71, -5.23) |
| Niger | 323.69 (194.47, 534.33) | 409.78 (257.65, 624.85) | 26.60% | 7.98 (4.79, 13.17) | 3.41 (2.14, 5.20) | -2.95 (-3.50, -2.39) |
| Nigeria | 1201.71 (736.41, 2011.53) | 1798.43 (1237.55, 3145.18) | 49.66% | 3.05 (1.87, 5.11) | 1.92 (1.32, 3.35) | -1.55 (-1.96, -1.14) |
| Niue | 0.01 (0.01, 0.02) | 0.01 (0.00, 0.01) | -53.42% | 1.72 (1.18, 2.47) | 1.65 (1.04, 2.57) | -0.14 (-0.33, 0.06) |
| North Macedonia | 11.36 (8.69, 14.05) | 2.02 (1.55, 2.56) | -82.26% | 2.12 (1.62, 2.62) | 0.58 (0.45, 0.74) | -4.51 (-6.04, -2.96) |
| Northern Mariana Islands | 0.10 (0.07, 0.13) | 0.05 (0.03, 0.06) | -52.75% | 0.77 (0.54, 1.05) | 0.61 (0.43, 0.84) | -0.82 (-1.32, -0.32) |
| Norway | 2.75 (2.33, 3.07) | 0.27 (0.17, 0.40) | -90.15% | 0.34 (0.29, 0.38) | 0.03 (0.02, 0.04) | -8.22 (-9.35, -7.07) |
| Oman | 52.61 (30.32, 82.42) | 17.68 (12.63, 23.43) | -66.39% | 6.31 (3.64, 9.89) | 1.72 (1.23, 2.28) | -4.37 (-4.95, -3.78) |
| Pakistan | 949.03 (577.62, 1489.29) | 1270.20 (898.89, 1658.87) | 33.84% | 1.87 (1.14, 2.94) | 1.46 (1.03, 1.90) | -0.96 (-1.22, -0.69) |
| Palau | 0.11 (0.07, 0.17) | 0.04 (0.03, 0.05) | -67.38% | 2.36 (1.42, 3.58) | 1.08 (0.78, 1.46) | -2.73 (-3.12, -2.33) |
| Palestine | 19.17 (11.45, 29.42) | 6.47 (4.57, 10.13) | -66.23% | 1.93 (1.15, 2.96) | 0.35 (0.25, 0.55) | -5.78 (-6.21, -5.34) |
| Panama | 11.16 (9.03, 13.67) | 5.29 (3.74, 7.21) | -52.58% | 1.34 (1.08, 1.64) | 0.46 (0.33, 0.63) | -3.58 (-4.43, -2.71) |
| Papua New Guinea | 72.31 (34.62, 123.95) | 140.20 (72.55, 234.47) | 93.87% | 4.31 (2.06, 7.39) | 3.81 (1.97, 6.37) | -0.46 (-0.75, -0.17) |
| Paraguay | 15.26 (12.07, 19.25) | 4.91 (3.14, 7.61) | -67.79% | 0.91 (0.72, 1.15) | 0.25 (0.16, 0.39) | -4.30 (-5.16, -3.43) |
| Peru | 273.77 (189.24, 347.01) | 38.28 (25.19, 56.10) | -86.02% | 3.26 (2.25, 4.13) | 0.42 (0.28, 0.61) | -6.87 (-7.37, -6.37) |
| Philippines | 194.40 (138.01, 315.43) | 298.52 (241.49, 355.60) | 53.56% | 0.76 (0.54, 1.24) | 0.84 (0.68, 1.00) | 0.57 (-0.15, 1.30) |
| Poland | 60.94 (54.82, 75.08) | 5.46 (4.20, 6.92) | -91.03% | 0.64 (0.57, 0.79) | 0.09 (0.07, 0.12) | -6.87 (-7.39, -6.34) |
| Portugal | 17.42 (14.83, 21.26) | 0.59 (0.41, 0.95) | -96.62% | 0.82 (0.70, 1.01) | 0.04 (0.03, 0.07) | -9.89 (-10.75, -9.01) |
| Puerto Rico | 5.04 (4.13, 6.56) | 0.76 (0.53, 1.09) | -85.00% | 0.51 (0.41, 0.66) | 0.14 (0.10, 0.21) | -4.09 (-5.23, -2.94) |
| Qatar | 2.01 (1.34, 2.88) | 1.05 (0.70, 1.54) | -48.03% | 1.62 (1.08, 2.32) | 0.25 (0.17, 0.37) | -6.30 (-7.44, -5.15) |
| Republic of Korea | 198.64 (158.68, 244.12) | 7.25 (5.21, 11.77) | -96.35% | 1.74 (1.39, 2.13) | 0.11 (0.08, 0.17) | -9.18 (-9.50, -8.86) |
| Republic of Moldova | 1.85 (1.35, 2.59) | 0.26 (0.17, 0.43) | -86.18% | 0.15 (0.11, 0.21) | 0.04 (0.03, 0.08) | -4.15 (-4.99, -3.31) |
| Romania | 16.51 (9.19, 21.88) | 2.61 (1.72, 3.49) | -84.20% | 0.30 (0.16, 0.39) | 0.09 (0.06, 0.12) | -3.99 (-5.19, -2.78) |
| Russian Federation | 90.57 (63.61, 100.87) | 28.93 (22.96, 34.69) | -68.06% | 0.26 (0.18, 0.29) | 0.11 (0.09, 0.13) | -3.33 (-4.53, -2.11) |
| Rwanda | 66.43 (40.24, 105.48) | 21.23 (11.75, 34.28) | -68.04% | 1.96 (1.19, 3.12) | 0.44 (0.24, 0.71) | -4.91 (-5.96, -3.86) |
| Saint Kitts and Nevis | 0.17 (0.14, 0.20) | 0.04 (0.03, 0.06) | -74.50% | 1.23 (1.03, 1.44) | 0.37 (0.27, 0.49) | -3.78 (-4.57, -2.98) |
| Saint Lucia | 0.29 (0.23, 0.36) | 0.06 (0.04, 0.08) | -78.60% | 0.55 (0.43, 0.68) | 0.19 (0.13, 0.26) | -3.52 (-4.06, -2.97) |
| Saint Vincent and the Grenadines | 0.46 (0.36, 0.57) | 0.09 (0.06, 0.12) | -80.59% | 1.10 (0.85, 1.37) | 0.35 (0.26, 0.48) | -3.80 (-4.65, -2.94) |
| Samoa | 1.07 (0.74, 1.52) | 0.45 (0.28, 0.68) | -57.63% | 1.62 (1.12, 2.31) | 0.61 (0.38, 0.93) | -2.86 (-3.16, -2.56) |
| San Marino | 0.01 (0.01, 0.02) | 0.00 (0.00, 0.01) | -73.34% | 0.31 (0.18, 0.45) | 0.07 (0.05, 0.10) | -4.96 (-5.20, -4.72) |
| Sao Tome and Principe | 2.18 (1.54, 3.11) | 0.72 (0.41, 1.21) | -67.09% | 3.83 (2.70, 5.47) | 0.96 (0.55, 1.63) | -4.46 (-6.35, -2.53) |
| Saudi Arabia | 36.33 (21.29, 57.90) | 6.29 (4.02, 10.10) | -82.67% | 0.54 (0.32, 0.87) | 0.09 (0.06, 0.14) | -6.14 (-6.47, -5.81) |
| Senegal | 141.04 (95.15, 197.70) | 92.35 (58.29, 148.94) | -34.52% | 3.87 (2.61, 5.43) | 1.51 (0.96, 2.44) | -3.21 (-4.76, -1.64) |
| Serbia | 39.32 (26.88, 53.30) | 1.69 (1.13, 2.58) | -95.69% | 1.86 (1.27, 2.53) | 0.12 (0.08, 0.18) | -9.19 (-11.74, -6.57) |
| Seychelles | 0.14 (0.10, 0.18) | 0.07 (0.05, 0.10) | -45.67% | 0.58 (0.41, 0.76) | 0.34 (0.24, 0.45) | -2.16 (-5.22, 1.00) |
| Sierra Leone | 152.25 (86.73, 241.92) | 173.13 (103.09, 277.57) | 13.71% | 9.54 (5.43, 15.16) | 5.18 (3.09, 8.31) | -2.12 (-2.47, -1.77) |
| Singapore | 4.36 (3.46, 6.07) | 0.51 (0.34, 0.77) | -88.27% | 0.67 (0.53, 0.94) | 0.07 (0.04, 0.10) | -7.96 (-9.27, -6.64) |
| Slovakia | 6.26 (4.90, 8.18) | 0.92 (0.59, 1.51) | -85.24% | 0.47 (0.37, 0.62) | 0.11 (0.07, 0.18) | -5.23 (-7.08, -3.33) |
| Slovenia | 1.97 (1.57, 2.37) | 0.21 (0.13, 0.29) | -89.41% | 0.48 (0.38, 0.57) | 0.07 (0.04, 0.09) | -6.53 (-7.54, -5.50) |
| Solomon Islands | 4.14 (2.14, 7.25) | 3.59 (2.19, 5.65) | -13.31% | 2.64 (1.36, 4.61) | 1.39 (0.85, 2.19) | -2.16 (-2.75, -1.56) |
| Somalia | 65.08 (34.73, 108.93) | 104.00 (64.52, 169.10) | 59.82% | 1.88 (1.00, 3.14) | 1.09 (0.68, 1.78) | -1.87 (-2.15, -1.58) |
| South Africa | 125.39 (70.96, 191.78) | 30.06 (22.13, 40.39) | -76.03% | 0.95 (0.54, 1.45) | 0.20 (0.15, 0.27) | -4.90 (-5.59, -4.20) |
| South Sudan | 59.51 (30.47, 102.84) | 42.41 (25.80, 65.78) | -28.73% | 2.28 (1.17, 3.94) | 1.02 (0.62, 1.58) | -2.76 (-3.19, -2.34) |
| Spain | 79.74 (70.98, 93.61) | 8.33 (6.39, 10.40) | -89.55% | 1.02 (0.91, 1.19) | 0.12 (0.09, 0.15) | -7.07 (-7.83, -6.31) |
| Sri Lanka | 43.02 (29.51, 59.12) | 13.89 (7.86, 20.54) | -67.70% | 0.77 (0.53, 1.05) | 0.27 (0.15, 0.40) | -3.19 (-4.06, -2.30) |
| Sudan | 908.96 (395.89, 1822.76) | 398.60 (223.34, 656.73) | -56.15% | 10.01 (4.36, 20.08) | 2.52 (1.41, 4.16) | -4.65 (-5.02, -4.28) |
| Suriname | 2.94 (2.05, 4.00) | 1.02 (0.69, 1.51) | -65.32% | 2.25 (1.57, 3.06) | 0.70 (0.47, 1.03) | -3.96 (-4.90, -3.01) |
| Sweden | 4.62 (3.74, 5.63) | 1.05 (0.74, 1.42) | -77.36% | 0.30 (0.24, 0.36) | 0.06 (0.04, 0.08) | -6.03 (-6.85, -5.21) |
| Switzerland | 1.47 (1.06, 1.92) | 0.28 (0.17, 0.38) | -81.07% | 0.13 (0.09, 0.17) | 0.02 (0.01, 0.03) | -6.27 (-7.76, -4.77) |
| Syrian Arab Republic | 879.76 (523.83, 1226.32) | 103.55 (77.85, 133.85) | -88.23% | 14.47 (8.62, 20.18) | 2.56 (1.92, 3.30) | -5.76 (-6.90, -4.60) |
| Taiwan (Province of China) | 39.71 (35.17, 44.43) | 3.28 (2.32, 4.68) | -91.74% | 0.72 (0.64, 0.81) | 0.11 (0.08, 0.15) | -6.25 (-8.28, -4.17) |
| Tajikistan | 6.12 (4.09, 8.22) | 3.14 (2.12, 4.74) | -48.74% | 0.26 (0.18, 0.36) | 0.10 (0.07, 0.15) | -3.38 (-3.87, -2.88) |
| Thailand | 150.94 (99.48, 230.19) | 48.58 (35.12, 64.82) | -67.81% | 0.89 (0.58, 1.35) | 0.46 (0.33, 0.61) | -2.20 (-2.75, -1.65) |
| Timor-Leste | 8.88 (4.26, 14.66) | 5.63 (3.04, 8.67) | -36.60% | 2.65 (1.27, 4.38) | 1.12 (0.60, 1.72) | -2.94 (-3.33, -2.55) |
| Togo | 61.69 (41.50, 87.92) | 51.81 (33.65, 79.65) | -16.01% | 3.47 (2.33, 4.94) | 1.65 (1.07, 2.53) | -2.50 (-2.73, -2.27) |
| Tokelau | 0.01 (0.01, 0.02) | 0.00 (0.00, 0.00) | -76.82% | 1.61 (0.97, 2.53) | 0.56 (0.35, 0.84) | -3.61 (-4.05, -3.18) |
| Tonga | 0.40 (0.28, 0.54) | 0.24 (0.16, 0.34) | -41.02% | 1.00 (0.71, 1.36) | 0.66 (0.44, 0.94) | -1.45 (-1.99, -0.90) |
| Trinidad and Tobago | 4.53 (3.69, 5.44) | 1.02 (0.72, 1.36) | -77.48% | 1.12 (0.91, 1.35) | 0.38 (0.27, 0.50) | -3.81 (-4.54, -3.07) |
| Tunisia | 74.95 (48.39, 119.87) | 10.15 (6.39, 15.05) | -86.46% | 2.35 (1.52, 3.76) | 0.38 (0.24, 0.57) | -6.06 (-6.37, -5.76) |
| Turkey | 494.17 (287.67, 818.81) | 50.20 (36.63, 68.94) | -89.84% | 2.29 (1.33, 3.79) | 0.32 (0.23, 0.44) | -6.50 (-6.79, -6.22) |
| Turkmenistan | 11.43 (7.98, 16.38) | 4.72 (2.97, 7.64) | -58.71% | 0.76 (0.53, 1.09) | 0.31 (0.20, 0.50) | -3.09 (-3.66, -2.52) |
| Tuvalu | 0.17 (0.09, 0.30) | 0.03 (0.02, 0.04) | -82.67% | 5.16 (2.83, 9.11) | 0.86 (0.58, 1.26) | -5.92 (-6.25, -5.58) |
| Uganda | 109.69 (65.63, 172.37) | 106.70 (65.58, 168.63) | -2.73% | 1.30 (0.78, 2.04) | 0.56 (0.34, 0.88) | -2.94 (-3.49, -2.39) |
| Ukraine | 12.81 (9.42, 16.25) | 7.75 (4.82, 10.50) | -39.48% | 0.11 (0.08, 0.14) | 0.11 (0.07, 0.15) | -0.07 (-1.81, 1.71) |
| United Arab Emirates | 17.90 (10.71, 25.32) | 4.35 (2.68, 6.61) | -75.68% | 3.03 (1.81, 4.28) | 0.37 (0.23, 0.56) | -7.08 (-7.47, -6.69) |
| United Kingdom | 48.46 (42.37, 56.43) | 9.21 (7.36, 13.65) | -80.99% | 0.44 (0.39, 0.52) | 0.08 (0.06, 0.12) | -5.93 (-6.38, -5.48) |
| United Republic of Tanzania | 249.25 (146.53, 394.53) | 228.43 (128.52, 403.41) | -8.35% | 2.05 (1.21, 3.25) | 0.93 (0.52, 1.64) | -2.56 (-2.82, -2.29) |
| United States of America | 330.92 (309.12, 369.06) | 161.09 (141.65, 182.81) | -51.32% | 0.59 (0.56, 0.66) | 0.27 (0.24, 0.30) | -2.74 (-3.26, -2.21) |
| United States Virgin Islands | 0.13 (0.09, 0.17) | 0.02 (0.01, 0.02) | -87.29% | 0.40 (0.28, 0.54) | 0.08 (0.05, 0.12) | -5.48 (-6.50, -4.45) |
| Uruguay | 6.38 (5.38, 7.47) | 1.48 (1.10, 1.94) | -76.80% | 0.78 (0.66, 0.91) | 0.21 (0.16, 0.28) | -4.56 (-6.16, -2.94) |
| Uzbekistan | 12.04 (9.40, 15.16) | 5.62 (3.91, 7.72) | -53.30% | 0.14 (0.11, 0.18) | 0.05 (0.04, 0.07) | -3.24 (-3.73, -2.75) |
| Vanuatu | 1.37 (0.84, 2.01) | 2.03 (1.28, 3.10) | 48.05% | 2.05 (1.26, 3.00) | 1.84 (1.15, 2.79) | -0.32 (-1.06, 0.42) |
| Venezuela (Bolivarian Republic of) | 39.74 (33.00, 46.53) | 16.26 (11.49, 22.47) | -59.09% | 0.56 (0.46, 0.65) | 0.23 (0.16, 0.32) | -2.92 (-3.95, -1.89) |
| Viet Nam | 440.26 (258.57, 692.72) | 106.09 (65.99, 165.42) | -75.90% | 1.68 (0.99, 2.64) | 0.50 (0.31, 0.78) | -4.09 (-4.22, -3.96) |
| Yemen | 346.68 (179.14, 755.87) | 201.23 (127.79, 298.65) | -41.96% | 4.83 (2.49, 10.53) | 1.55 (0.99, 2.31) | -3.84 (-4.07, -3.61) |
| Zambia | 86.21 (49.81, 140.13) | 39.88 (24.88, 63.25) | -53.74% | 2.29 (1.32, 3.73) | 0.52 (0.32, 0.82) | -5.07 (-5.54, -4.61) |
| Zimbabwe | 28.56 (15.28, 46.57) | 45.06 (24.80, 73.95) | 57.76% | 0.59 (0.32, 0.97) | 0.76 (0.42, 1.24) | 0.91 (0.44, 1.37) |

Note: ASR: age-standardized rates; AAPC, average annual percentage change; CI, confidence interval; UI, uncertainty interval

Table S6**. The DALYs and age-standardized DALY rate due to stroke and their temporal change among children (0-14 years) in 204 countries or territories from 1990 to 2019.**

|  | DALYs (95% UI) | |  | Age-standardized DALY rate (95% UI), per 100,000 population | | |
| --- | --- | --- | --- | --- | --- | --- |
| **Countries or territories** | 1990 | 2019 | Percentage change (%) | 1990 | 2019 | AAPC (95% CI) |
| Afghanistan | 10963.97 (6700.87, 18123.73) | 12318.82 (8791.87, 19757.48) | 12.35725 | 219.74 (134.30, 363.23) | 71.38 (50.94, 114.48) | -3.71 (-4.36, -3.06) |
| Albania | 3045.05 (2413.84, 3918.87) | 1189.47 (693.77, 1680.75) | -60.9377 | 270.99 (214.82, 348.75) | 251.53 (146.71, 355.42) | -0.31 (-1.06, 0.43) |
| Algeria | 29427.55 (18367.33, 50180.08) | 8399.09 (6064.33, 11307.57) | -71.4584 | 274.50 (171.33, 468.07) | 71.56 (51.66, 96.33) | -4.50 (-4.74, -4.25) |
| American Samoa | 17.39 (13.19, 22.32) | 9.76 (7.31, 13.01) | -43.9089 | 92.00 (69.74, 118.03) | 58.77 (44.06, 78.39) | -1.48 (-1.82, -1.14) |
| Andorra | 3.20 (2.05, 4.58) | 1.43 (1.04, 1.90) | -55.4573 | 33.71 (21.56, 48.24) | 13.34 (9.71, 17.77) | -3.10 (-3.39, -2.81) |
| Angola | 16155.81 (7767.02, 32250.68) | 12408.01 (8423.15, 17870.02) | -23.1979 | 340.25 (163.58, 679.22) | 88.71 (60.22, 127.76) | -4.59 (-4.90, -4.28) |
| Antigua and Barbuda | 8.38 (6.88, 10.20) | 4.83 (3.75, 6.06) | -42.3046 | 44.80 (36.82, 54.53) | 28.63 (22.23, 35.91) | -1.54 (-1.84, -1.24) |
| Argentina | 13194.50 (11140.43, 15536.26) | 3379.15 (2742.48, 4068.17) | -74.3897 | 130.21 (109.94, 153.32) | 31.95 (25.93, 38.47) | -4.81 (-5.25, -4.37) |
| Armenia | 305.53 (238.22, 385.59) | 105.21 (73.99, 138.05) | -65.5639 | 29.33 (22.87, 37.02) | 17.29 (12.16, 22.68) | -1.75 (-2.32, -1.19) |
| Australia | 1163.25 (956.27, 1396.96) | 643.87 (476.22, 840.83) | -44.6495 | 30.72 (25.25, 36.89) | 14.02 (10.37, 18.31) | -2.56 (-2.89, -2.23) |
| Austria | 566.35 (463.68, 664.16) | 201.88 (150.39, 262.23) | -64.3549 | 41.99 (34.38, 49.24) | 15.66 (11.67, 20.34) | -3.34 (-3.63, -3.04) |
| Azerbaijan | 694.71 (514.79, 953.37) | 488.53 (353.75, 645.19) | -29.6785 | 28.59 (21.19, 39.24) | 21.00 (15.20, 27.73) | -1.14 (-2.08, -0.18) |
| Bahamas | 42.49 (33.85, 53.32) | 19.64 (15.30, 24.85) | -53.7768 | 52.85 (42.10, 66.31) | 24.58 (19.15, 31.10) | -2.62 (-3.30, -1.95) |
| Bahrain | 328.97 (222.20, 466.49) | 95.18 (74.22, 121.92) | -71.0661 | 200.58 (135.48, 284.43) | 40.54 (31.61, 51.92) | -5.39 (-6.17, -4.61) |
| Bangladesh | 108421.11 (66196.17, 149236.68) | 38377.78 (28245.47, 49568.94) | -64.603 | 223.84 (136.66, 308.10) | 87.13 (64.12, 112.53) | -3.13 (-3.33, -2.92) |
| Barbados | 30.04 (24.78, 36.00) | 12.76 (9.67, 16.33) | -57.5242 | 47.87 (39.49, 57.36) | 25.83 (19.58, 33.05) | -2.04 (-2.60, -1.49) |
| Belarus | 574.91 (425.68, 754.61) | 293.08 (199.64, 403.61) | -49.0218 | 23.83 (17.64, 31.27) | 17.83 (12.14, 24.55) | -1.02 (-1.35, -0.69) |
| Belgium | 1085.92 (939.17, 1248.00) | 353.81 (277.00, 441.24) | -67.4184 | 60.11 (51.99, 69.09) | 18.30 (14.33, 22.82) | -4.22 (-4.46, -3.99) |
| Belize | 81.52 (63.12, 104.03) | 35.90 (28.49, 44.09) | -55.9594 | 100.90 (78.12, 128.77) | 29.31 (23.25, 35.99) | -4.36 (-5.39, -3.32) |
| Benin | 8200.45 (5256.02, 12116.11) | 12049.75 (8188.28, 17410.06) | 46.94004 | 338.22 (216.78, 499.71) | 209.14 (142.12, 302.17) | -1.62 (-1.87, -1.36) |
| Bermuda | 3.52 (2.75, 4.42) | 1.29 (0.95, 1.68) | -63.5302 | 29.52 (23.05, 37.06) | 14.58 (10.83, 19.12) | -2.41 (-2.64, -2.18) |
| Bhutan | 284.03 (113.00, 520.20) | 87.58 (62.64, 119.16) | -69.1645 | 112.18 (44.63, 205.45) | 45.00 (32.18, 61.22) | -3.15 (-3.58, -2.72) |
| Bolivia (Plurinational State of) | 8358.81 (5231.08, 12853.88) | 2972.45 (2150.17, 4035.20) | -64.4393 | 306.16 (191.60, 470.81) | 75.71 (54.77, 102.78) | -4.74 (-4.86, -4.61) |
| Bosnia and Herzegovina | 1092.49 (886.57, 1312.33) | 246.10 (191.92, 305.53) | -77.4738 | 98.87 (80.23, 118.77) | 51.65 (40.28, 64.12) | -2.29 (-3.81, -0.74) |
| Botswana | 394.39 (275.94, 530.29) | 563.48 (340.62, 900.92) | 42.87415 | 69.03 (48.29, 92.81) | 79.90 (48.30, 127.75) | 0.52 (-0.23, 1.27) |
| Brazil | 61608.41 (50643.79, 75274.41) | 20331.82 (16770.11, 24043.12) | -66.9983 | 117.86 (96.89, 144.01) | 42.58 (35.12, 50.36) | -3.41 (-3.67, -3.15) |
| Brunei Darussalam | 62.99 (47.94, 78.62) | 26.02 (19.34, 34.46) | -58.6993 | 69.47 (52.88, 86.71) | 27.35 (20.34, 36.23) | -3.22 (-3.71, -2.74) |
| Bulgaria | 2427.79 (2081.25, 2844.87) | 505.89 (373.71, 703.08) | -79.1626 | 139.80 (119.84, 163.81) | 51.11 (37.75, 71.03) | -3.46 (-4.32, -2.60) |
| Burkina Faso | 14457.19 (9714.77, 21536.22) | 28566.19 (18358.14, 47255.61) | 97.59154 | 305.76 (205.46, 455.47) | 274.85 (176.63, 454.67) | -0.27 (-0.51, -0.02) |
| Burundi | 5763.92 (3394.57, 9105.42) | 4727.13 (3241.96, 7555.55) | -17.9875 | 218.41 (128.63, 345.03) | 87.52 (60.02, 139.88) | -3.15 (-3.65, -2.65) |
| Cabo Verde | 352.52 (240.38, 532.71) | 119.06 (85.32, 161.81) | -66.2274 | 227.24 (154.95, 343.39) | 74.89 (53.67, 101.78) | -3.62 (-4.23, -3.01) |
| Cambodia | 20137.91 (8097.41, 37545.81) | 5653.35 (2870.97, 9073.73) | -71.9268 | 422.20 (169.77, 787.17) | 112.32 (57.04, 180.27) | -4.46 (-4.66, -4.26) |
| Cameroon | 11182.05 (7360.08, 16497.52) | 22977.22 (15237.33, 35916.28) | 105.483 | 231.08 (152.10, 340.92) | 190.88 (126.58, 298.36) | -0.62 (-1.11, -0.13) |
| Canada | 2417.62 (2011.03, 2918.99) | 1272.70 (961.64, 1645.99) | -47.3574 | 42.02 (34.95, 50.74) | 20.86 (15.76, 26.97) | -2.29 (-2.92, -1.65) |
| Central African Republic | 3217.79 (1664.36, 5692.66) | 4026.38 (2357.00, 6507.01) | 25.12883 | 262.77 (135.91, 464.86) | 180.28 (105.54, 291.35) | -1.28 (-1.61, -0.94) |
| Chad | 12591.83 (8168.48, 18259.88) | 25609.88 (18081.93, 35811.42) | 103.3849 | 430.86 (279.51, 624.81) | 308.18 (217.59, 430.94) | -1.06 (-1.43, -0.69) |
| Chile | 1884.77 (1602.90, 2183.92) | 744.04 (588.96, 915.89) | -60.5235 | 47.39 (40.30, 54.91) | 20.50 (16.23, 25.24) | -2.84 (-3.09, -2.58) |
| China | 1000604.59 (690219.66, 1222393.98) | 93755.94 (74164.53, 120520.29) | -90.6301 | 309.87 (213.75, 378.56) | 41.71 (32.99, 53.62) | -6.68 (-7.08, -6.29) |
| Colombia | 14167.46 (12413.42, 16279.15) | 5257.74 (3854.57, 6802.28) | -62.8886 | 120.98 (106.00, 139.01) | 47.69 (34.96, 61.70) | -3.19 (-3.67, -2.71) |
| Comoros | 327.34 (175.54, 475.87) | 160.18 (111.85, 225.85) | -51.0671 | 154.08 (82.63, 223.99) | 68.81 (48.05, 97.02) | -2.79 (-4.04, -1.52) |
| Congo | 1741.37 (1064.10, 2696.71) | 1221.04 (871.69, 1766.62) | -29.8801 | 158.86 (97.08, 246.02) | 61.43 (43.85, 88.87) | -3.18 (-3.34, -3.01) |
| Cook Islands | 9.91 (7.04, 14.03) | 1.57 (1.11, 2.07) | -84.2018 | 147.70 (104.90, 209.14) | 37.44 (26.43, 49.58) | -4.59 (-4.87, -4.32) |
| Costa Rica | 423.69 (355.07, 513.80) | 197.68 (151.57, 255.39) | -53.3438 | 37.74 (31.62, 45.76) | 18.74 (14.37, 24.21) | -2.29 (-2.55, -2.02) |
| Croatia | 717.89 (615.19, 835.08) | 157.37 (119.36, 202.85) | -78.0792 | 72.75 (62.34, 84.62) | 25.82 (19.58, 33.28) | -3.43 (-4.19, -2.67) |
| Cuba | 947.90 (773.85, 1156.41) | 346.14 (260.40, 448.80) | -63.4833 | 37.93 (30.97, 46.28) | 19.28 (14.50, 25.00) | -2.22 (-2.54, -1.90) |
| Cyprus | 164.35 (108.14, 242.43) | 44.90 (33.84, 57.58) | -72.6812 | 82.96 (54.59, 122.38) | 21.07 (15.88, 27.02) | -4.51 (-5.62, -3.38) |
| Czechia | 1221.68 (978.97, 1601.84) | 309.67 (213.60, 432.89) | -74.652 | 55.58 (44.54, 72.88) | 18.17 (12.53, 25.40) | -3.81 (-4.24, -3.37) |
| C么te d'Ivoire | 21024.95 (14686.52, 29017.18) | 20997.16 (13727.28, 30728.97) | -0.13218 | 366.94 (256.32, 506.42) | 196.54 (128.49, 287.63) | -2.14 (-2.42, -1.87) |
| Democratic People's Republic of Korea | 22195.41 (12592.51, 36322.38) | 2883.93 (1978.79, 4100.04) | -87.0067 | 340.85 (193.38, 557.79) | 58.85 (40.38, 83.67) | -5.91 (-6.05, -5.77) |
| Democratic Republic of the Congo | 48778.79 (25764.93, 79293.09) | 28166.28 (19256.28, 41564.49) | -42.2571 | 268.90 (142.04, 437.12) | 74.72 (51.08, 110.26) | -4.37 (-4.51, -4.23) |
| Denmark | 293.80 (234.32, 361.13) | 122.41 (86.35, 168.15) | -58.335 | 33.27 (26.53, 40.89) | 12.75 (8.99, 17.51) | -3.29 (-3.58, -3.00) |
| Djibouti | 268.48 (182.55, 399.05) | 311.72 (209.34, 465.29) | 16.10644 | 122.16 (83.06, 181.57) | 74.11 (49.77, 110.63) | -1.82 (-2.17, -1.46) |
| Dominica | 8.52 (6.77, 10.55) | 4.73 (3.45, 6.26) | -44.4917 | 34.73 (27.60, 42.99) | 32.66 (23.79, 43.19) | -0.20 (-0.61, 0.22) |
| Dominican Republic | 5130.07 (3802.21, 6861.37) | 2104.57 (1482.41, 3016.28) | -58.9759 | 187.01 (138.60, 250.12) | 69.14 (48.70, 99.10) | -3.45 (-3.85, -3.06) |
| Ecuador | 3473.16 (2941.75, 4122.58) | 2337.81 (1824.29, 2982.56) | -32.6892 | 88.82 (75.23, 105.43) | 46.45 (36.25, 59.26) | -2.31 (-2.86, -1.76) |
| Egypt | 860224.97 (380331.38, 1199924.88) | 176927.65 (98718.70, 280946.81) | -79.4324 | 3864.63 (1708.67, 5390.75) | 541.76 (302.28, 860.27) | -6.56 (-6.92, -6.20) |
| El Salvador | 6683.36 (5135.00, 8058.34) | 781.96 (582.42, 1040.52) | -88.2998 | 314.53 (241.67, 379.24) | 45.81 (34.12, 60.96) | -6.47 (-7.05, -5.89) |
| Equatorial Guinea | 498.91 (265.48, 815.92) | 253.99 (167.04, 361.41) | -49.0903 | 242.96 (129.28, 397.33) | 45.00 (29.60, 64.03) | -5.66 (-6.43, -4.89) |
| Eritrea | 2025.67 (1214.36, 3175.80) | 1643.02 (1166.39, 2470.50) | -18.8901 | 143.81 (86.21, 225.46) | 62.21 (44.16, 93.54) | -2.87 (-3.13, -2.60) |
| Estonia | 123.30 (99.60, 150.77) | 35.63 (24.48, 48.42) | -71.1001 | 35.30 (28.52, 43.17) | 16.53 (11.36, 22.46) | -2.58 (-2.92, -2.24) |
| Eswatini | 409.68 (243.75, 624.71) | 297.64 (218.45, 394.85) | -27.3465 | 106.31 (63.25, 162.11) | 72.74 (53.38, 96.49) | -1.34 (-1.54, -1.14) |
| Ethiopia | 53232.97 (29782.30, 83473.58) | 27874.06 (19691.50, 39134.68) | -47.6376 | 214.86 (120.21, 336.91) | 60.51 (42.75, 84.95) | -4.22 (-4.62, -3.83) |
| Fiji | 414.18 (311.97, 533.51) | 407.10 (300.18, 558.35) | -1.70891 | 146.83 (110.59, 189.14) | 153.33 (113.06, 210.30) | 0.14 (-0.16, 0.43) |
| Finland | 416.50 (330.08, 503.86) | 154.35 (112.21, 204.06) | -62.9412 | 43.14 (34.19, 52.18) | 17.65 (12.83, 23.33) | -2.90 (-3.47, -2.32) |
| France | 5197.21 (4530.15, 5907.25) | 2100.23 (1667.87, 2599.24) | -59.5893 | 44.38 (38.69, 50.45) | 17.78 (14.12, 22.01) | -3.18 (-3.62, -2.73) |
| Gabon | 537.38 (324.50, 790.06) | 275.19 (196.54, 385.14) | -48.791 | 128.27 (77.46, 188.59) | 47.24 (33.74, 66.11) | -3.43 (-3.64, -3.22) |
| Gambia | 1197.97 (795.18, 1690.27) | 1102.90 (702.96, 1960.79) | -7.93634 | 254.41 (168.87, 358.96) | 119.25 (76.01, 212.00) | -2.61 (-3.54, -1.67) |
| Georgia | 818.10 (654.02, 1004.83) | 194.46 (147.26, 250.33) | -76.2303 | 59.86 (47.86, 73.53) | 26.82 (20.31, 34.52) | -2.79 (-3.41, -2.17) |
| Germany | 6231.21 (5202.18, 7364.53) | 1854.47 (1400.56, 2356.76) | -70.2389 | 48.10 (40.16, 56.85) | 15.99 (12.07, 20.32) | -3.70 (-4.13, -3.27) |
| Ghana | 12894.04 (9270.34, 17748.09) | 12510.82 (8567.25, 17660.45) | -2.97208 | 190.99 (137.32, 262.90) | 110.65 (75.77, 156.19) | -1.84 (-2.19, -1.49) |
| Greece | 961.17 (816.25, 1142.06) | 306.45 (242.36, 387.17) | -68.1174 | 47.52 (40.35, 56.46) | 20.97 (16.58, 26.49) | -2.68 (-3.17, -2.19) |
| Greenland | 41.01 (28.01, 58.13) | 5.35 (3.71, 7.57) | -86.9517 | 288.46 (197.04, 408.92) | 45.89 (31.81, 64.96) | -6.25 (-6.68, -5.82) |
| Grenada | 23.05 (18.19, 28.76) | 7.27 (5.68, 9.11) | -68.4685 | 71.91 (56.75, 89.72) | 32.57 (25.44, 40.81) | -2.65 (-2.88, -2.42) |
| Guam | 42.24 (33.53, 53.52) | 45.90 (35.40, 58.31) | 8.678465 | 101.23 (80.36, 128.27) | 100.85 (77.77, 128.12) | -0.03 (-0.66, 0.61) |
| Guatemala | 6141.49 (4882.13, 7353.08) | 4028.21 (2937.71, 5616.18) | -34.4099 | 168.36 (133.83, 201.57) | 69.12 (50.41, 96.37) | -2.95 (-3.34, -2.56) |
| Guinea | 15858.54 (9781.01, 25721.57) | 17555.44 (11190.93, 26352.87) | 10.70026 | 559.05 (344.80, 906.74) | 307.28 (195.88, 461.26) | -2.02 (-2.43, -1.61) |
| Guinea-Bissau | 2284.38 (1469.80, 3470.36) | 1588.96 (1095.04, 2350.63) | -30.4425 | 479.85 (308.74, 728.97) | 199.95 (137.80, 295.80) | -2.92 (-3.28, -2.56) |
| Guyana | 637.16 (513.73, 774.23) | 169.14 (121.87, 226.88) | -73.4548 | 223.87 (180.50, 272.03) | 81.05 (58.40, 108.72) | -3.48 (-4.35, -2.59) |
| Haiti | 25122.08 (11979.72, 39821.42) | 17818.36 (10094.14, 28550.60) | -29.0729 | 933.56 (445.18, 1479.80) | 416.67 (236.04, 667.63) | -2.73 (-2.92, -2.54) |
| Honduras | 9333.82 (6405.81, 12435.67) | 3349.92 (1939.55, 5221.32) | -64.1099 | 422.99 (290.30, 563.56) | 101.53 (58.79, 158.26) | -4.79 (-5.08, -4.49) |
| Hungary | 1248.06 (1038.52, 1490.67) | 371.43 (269.42, 488.63) | -70.2399 | 58.66 (48.81, 70.06) | 26.80 (19.44, 35.26) | -2.60 (-3.12, -2.08) |
| Iceland | 35.54 (29.52, 42.15) | 10.58 (7.93, 13.62) | -70.22 | 55.99 (46.51, 66.40) | 15.79 (11.83, 20.33) | -4.23 (-5.03, -3.43) |
| India | 331680.40 (255553.22, 432201.14) | 161986.47 (131444.43, 198627.51) | -51.1619 | 100.18 (77.19, 130.54) | 43.06 (34.94, 52.81) | -2.88 (-3.32, -2.44) |
| Indonesia | 159060.47 (97806.19, 232009.10) | 48469.09 (38849.22, 60802.12) | -69.5279 | 233.52 (143.59, 340.62) | 74.44 (59.67, 93.39) | -3.87 (-4.14, -3.60) |
| Iran (Islamic Republic of) | 48379.59 (35504.30, 68605.78) | 10442.73 (7925.62, 13026.38) | -78.415 | 186.70 (137.02, 264.76) | 51.20 (38.86, 63.86) | -4.37 (-4.79, -3.95) |
| Iraq | 29863.28 (19448.37, 42913.52) | 14461.05 (10780.03, 19464.00) | -51.5758 | 369.55 (240.67, 531.05) | 104.97 (78.25, 141.28) | -4.19 (-4.94, -3.44) |
| Ireland | 291.75 (240.41, 349.20) | 131.02 (95.94, 178.33) | -55.0908 | 29.72 (24.49, 35.57) | 12.84 (9.40, 17.47) | -2.86 (-3.32, -2.40) |
| Israel | 771.50 (631.62, 946.47) | 368.42 (278.95, 476.34) | -52.2466 | 50.34 (41.21, 61.76) | 14.23 (10.77, 18.40) | -4.21 (-4.64, -3.78) |
| Italy | 5771.23 (5226.17, 6576.41) | 1624.92 (1305.86, 2003.14) | -71.8444 | 62.54 (56.63, 71.26) | 20.44 (16.43, 25.20) | -3.76 (-4.38, -3.12) |
| Jamaica | 1959.74 (1563.87, 2424.34) | 382.48 (287.33, 499.42) | -80.4832 | 235.11 (187.62, 290.84) | 61.81 (46.43, 80.70) | -4.58 (-5.34, -3.81) |
| Japan | 10231.79 (8948.58, 11496.03) | 3375.57 (2608.28, 4286.93) | -67.009 | 44.36 (38.79, 49.84) | 21.63 (16.71, 27.47) | -2.53 (-2.81, -2.24) |
| Jordan | 1172.30 (886.35, 1595.54) | 1347.08 (996.98, 1828.46) | 14.90909 | 70.46 (53.28, 95.90) | 36.18 (26.78, 49.11) | -2.16 (-2.34, -1.98) |
| Kazakhstan | 2792.37 (2209.09, 3340.58) | 1599.14 (1212.68, 2152.37) | -42.7319 | 54.05 (42.76, 64.66) | 31.52 (23.90, 42.42) | -1.93 (-2.55, -1.30) |
| Kenya | 10540.75 (7454.80, 13361.41) | 9948.88 (7544.77, 12927.63) | -5.61512 | 93.91 (66.42, 119.04) | 51.93 (39.38, 67.47) | -2.03 (-2.28, -1.77) |
| Kiribati | 122.85 (86.09, 167.81) | 76.02 (51.03, 108.79) | -38.1196 | 420.83 (294.90, 574.83) | 181.46 (121.80, 259.68) | -2.90 (-3.30, -2.51) |
| Kuwait | 506.36 (404.57, 646.07) | 435.11 (346.75, 545.95) | -14.0707 | 89.56 (71.56, 114.28) | 50.51 (40.26, 63.38) | -1.70 (-2.66, -0.73) |
| Kyrgyzstan | 497.87 (400.84, 613.20) | 368.76 (274.97, 485.84) | -25.9322 | 29.88 (24.06, 36.80) | 17.66 (13.17, 23.27) | -1.83 (-2.14, -1.52) |
| Lao People's Democratic Republic | 6089.30 (3256.43, 9962.80) | 3325.16 (2128.51, 4962.38) | -45.3934 | 333.66 (178.44, 545.91) | 147.79 (94.61, 220.56) | -2.74 (-2.95, -2.53) |
| Latvia | 180.66 (139.35, 227.73) | 56.05 (38.78, 78.75) | -68.978 | 31.76 (24.49, 40.03) | 18.45 (12.76, 25.92) | -1.86 (-2.52, -1.21) |
| Lebanon | 808.41 (535.44, 1134.04) | 361.14 (247.91, 515.34) | -55.3272 | 65.32 (43.26, 91.63) | 26.05 (17.88, 37.17) | -3.11 (-3.31, -2.90) |
| Lesotho | 563.79 (386.12, 799.01) | 479.76 (340.72, 671.96) | -14.9044 | 74.03 (50.70, 104.91) | 72.60 (51.56, 101.69) | -0.14 (-0.62, 0.34) |
| Liberia | 5984.73 (3658.04, 9869.86) | 2733.50 (1867.21, 4092.68) | -54.3254 | 660.89 (403.95, 1089.92) | 146.33 (99.96, 219.09) | -5.23 (-5.56, -4.89) |
| Libya | 20183.44 (9397.10, 30579.88) | 3051.70 (1784.21, 4545.24) | -84.8802 | 1071.96 (499.09, 1624.12) | 207.80 (121.49, 309.50) | -5.52 (-5.85, -5.20) |
| Lithuania | 224.94 (168.93, 295.47) | 78.47 (52.05, 109.84) | -65.1123 | 27.13 (20.38, 35.64) | 18.87 (12.52, 26.41) | -1.29 (-1.86, -0.72) |
| Luxembourg | 43.89 (36.35, 51.99) | 15.80 (11.71, 20.48) | -64.0093 | 66.47 (55.05, 78.75) | 15.99 (11.86, 20.73) | -4.88 (-5.86, -3.89) |
| Madagascar | 17345.18 (11379.87, 25265.60) | 11755.19 (7338.26, 20133.29) | -32.2279 | 315.09 (206.73, 458.97) | 107.58 (67.16, 184.25) | -3.58 (-3.89, -3.28) |
| Malawi | 11843.75 (6671.75, 20302.03) | 5955.05 (4198.08, 8266.08) | -49.7199 | 267.36 (150.61, 458.29) | 75.59 (53.29, 104.93) | -4.34 (-5.35, -3.31) |
| Malaysia | 9596.89 (6456.87, 12706.05) | 4269.00 (3252.04, 5460.34) | -55.5168 | 145.90 (98.16, 193.16) | 55.48 (42.27, 70.97) | -2.92 (-3.45, -2.39) |
| Maldives | 556.18 (266.24, 890.58) | 73.70 (46.84, 123.03) | -86.7494 | 531.30 (254.33, 850.73) | 66.98 (42.57, 111.81) | -6.84 (-7.37, -6.31) |
| Mali | 21521.24 (13828.02, 34375.87) | 36591.71 (22276.91, 67988.40) | 70.02605 | 521.86 (335.31, 833.56) | 354.34 (215.72, 658.38) | -1.34 (-2.18, -0.49) |
| Malta | 52.73 (43.80, 63.77) | 15.32 (11.64, 19.49) | -70.9505 | 60.25 (50.05, 72.87) | 24.33 (18.49, 30.95) | -3.05 (-3.72, -2.37) |
| Marshall Islands | 29.71 (20.51, 41.67) | 22.18 (15.39, 31.13) | -25.3321 | 133.96 (92.50, 187.89) | 120.25 (83.43, 168.76) | -0.40 (-0.69, -0.12) |
| Mauritania | 2090.57 (1521.60, 2859.56) | 1543.37 (1032.93, 2309.95) | -26.1748 | 223.44 (162.63, 305.62) | 95.71 (64.05, 143.24) | -2.87 (-3.27, -2.47) |
| Mauritius | 270.81 (229.13, 322.61) | 79.15 (58.44, 101.99) | -70.7739 | 81.99 (69.37, 97.67) | 36.83 (27.19, 47.46) | -2.66 (-3.35, -1.96) |
| Mexico | 38661.30 (33305.14, 44046.10) | 17995.96 (13693.55, 26010.29) | -53.4523 | 115.35 (99.37, 131.42) | 55.73 (42.41, 80.56) | -2.41 (-2.59, -2.24) |
| Micronesia (Federated States of) | 88.05 (58.59, 127.13) | 26.90 (14.81, 38.16) | -69.4491 | 187.16 (124.54, 270.23) | 85.64 (47.16, 121.50) | -2.65 (-2.89, -2.42) |
| Monaco | 1.64 (1.10, 2.44) | 0.84 (0.61, 1.10) | -48.6701 | 46.33 (31.10, 69.09) | 16.92 (12.28, 22.12) | -3.41 (-3.65, -3.17) |
| Mongolia | 3814.26 (1907.30, 5272.68) | 734.31 (464.22, 991.57) | -80.7484 | 424.76 (212.40, 587.17) | 72.53 (45.86, 97.95) | -5.82 (-6.36, -5.28) |
| Montenegro | 117.14 (93.46, 143.38) | 28.88 (21.16, 38.66) | -75.3447 | 72.42 (57.77, 88.63) | 26.50 (19.41, 35.47) | -3.26 (-4.24, -2.26) |
| Morocco | 28320.98 (18745.51, 40858.29) | 7466.79 (4502.86, 10846.11) | -73.6351 | 289.10 (191.36, 417.09) | 79.14 (47.73, 114.96) | -4.28 (-4.54, -4.02) |
| Mozambique | 10624.11 (6332.46, 17349.26) | 9620.97 (6600.05, 14088.16) | -9.44211 | 174.38 (103.94, 284.76) | 69.90 (47.95, 102.36) | -3.22 (-3.79, -2.66) |
| Myanmar | 120254.88 (50899.28, 253781.73) | 43697.25 (19474.50, 69688.12) | -63.6628 | 766.81 (324.56, 1618.24) | 291.74 (130.02, 465.27) | -3.29 (-3.57, -3.01) |
| Namibia | 484.29 (282.94, 719.79) | 413.21 (284.55, 549.59) | -14.6765 | 81.39 (47.55, 120.97) | 49.34 (33.97, 65.62) | -1.79 (-2.27, -1.31) |
| Nauru | 14.97 (10.20, 22.48) | 10.70 (7.09, 15.54) | -28.5083 | 336.89 (229.59, 506.01) | 273.24 (180.98, 396.82) | -0.75 (-1.06, -0.43) |
| Nepal | 8515.94 (5354.84, 12897.57) | 2758.62 (1986.30, 3659.86) | -67.6064 | 100.18 (63.00, 151.73) | 29.92 (21.54, 39.70) | -4.08 (-4.45, -3.71) |
| Netherlands | 1087.59 (904.13, 1297.90) | 463.87 (349.58, 598.50) | -57.3492 | 39.92 (33.18, 47.63) | 16.97 (12.79, 21.89) | -2.85 (-3.26, -2.44) |
| New Zealand | 288.45 (246.98, 343.50) | 154.82 (115.42, 203.26) | -46.3277 | 36.06 (30.87, 42.94) | 17.35 (12.94, 22.78) | -2.54 (-3.11, -1.96) |
| Nicaragua | 1872.02 (1359.70, 2506.02) | 560.75 (426.28, 760.30) | -70.0457 | 102.51 (74.46, 137.23) | 28.03 (21.31, 38.00) | -4.35 (-4.68, -4.01) |
| Niger | 28407.58 (17268.36, 46570.90) | 36773.38 (23862.82, 55529.46) | 29.44915 | 700.42 (425.77, 1148.26) | 305.87 (198.49, 461.88) | -2.87 (-3.40, -2.34) |
| Nigeria | 109051.72 (68148.34, 180062.98) | 170751.46 (122347.70, 285562.52) | 56.57843 | 276.88 (173.03, 457.17) | 182.14 (130.51, 304.61) | -1.40 (-1.77, -1.02) |
| Niue | 1.36 (0.97, 1.88) | 0.63 (0.42, 0.96) | -53.2052 | 163.46 (116.80, 227.03) | 157.46 (104.61, 237.77) | -0.12 (-0.31, 0.06) |
| North Macedonia | 1031.90 (793.10, 1269.74) | 220.21 (174.92, 272.18) | -78.6593 | 192.51 (147.96, 236.89) | 63.74 (50.63, 78.78) | -3.98 (-5.49, -2.45) |
| Northern Mariana Islands | 10.17 (7.61, 13.18) | 5.29 (3.95, 6.82) | -47.9655 | 81.88 (61.29, 106.14) | 71.61 (53.54, 92.30) | -0.46 (-0.91, 0.00) |
| Norway | 319.38 (274.23, 371.10) | 142.07 (99.93, 196.98) | -55.5167 | 39.96 (34.31, 46.43) | 15.19 (10.68, 21.06) | -3.29 (-3.65, -2.93) |
| Oman | 4627.62 (2691.08, 7206.46) | 1662.67 (1227.59, 2164.65) | -64.0707 | 555.31 (322.93, 864.78) | 162.05 (119.65, 210.97) | -4.11 (-4.63, -3.59) |
| Pakistan | 88702.77 (55702.80, 135969.86) | 120332.34 (88902.79, 153495.18) | 35.65794 | 174.91 (109.84, 268.11) | 138.08 (102.02, 176.14) | -0.89 (-1.11, -0.66) |
| Palau | 10.37 (6.51, 15.31) | 3.72 (2.80, 4.84) | -64.0951 | 218.82 (137.27, 322.96) | 110.57 (83.18, 143.79) | -2.39 (-2.74, -2.04) |
| Palestine | 1788.18 (1116.94, 2690.37) | 843.51 (631.21, 1143.29) | -52.8284 | 179.63 (112.20, 270.26) | 45.46 (34.02, 61.61) | -4.64 (-5.03, -4.25) |
| Panama | 1043.43 (853.90, 1260.16) | 566.06 (426.01, 740.03) | -45.7501 | 125.13 (102.40, 151.13) | 49.49 (37.25, 64.70) | -3.13 (-3.73, -2.52) |
| Papua New Guinea | 6493.99 (3227.79, 10978.38) | 12639.71 (6828.57, 20926.15) | 94.63721 | 386.94 (192.32, 654.13) | 343.41 (185.53, 568.55) | -0.44 (-0.72, -0.16) |
| Paraguay | 1464.84 (1180.57, 1813.94) | 620.22 (444.24, 847.77) | -57.6595 | 87.56 (70.57, 108.42) | 31.51 (22.57, 43.07) | -3.47 (-4.09, -2.84) |
| Peru | 24124.39 (16719.03, 30468.04) | 3866.67 (2758.01, 5372.42) | -83.9719 | 287.06 (198.94, 362.54) | 42.34 (30.20, 58.83) | -6.51 (-6.98, -6.04) |
| Philippines | 20859.47 (15812.40, 30893.52) | 31489.21 (25896.92, 36788.30) | 50.95884 | 82.05 (62.19, 121.51) | 88.54 (72.81, 103.44) | 0.44 (-0.09, 0.98) |
| Poland | 6616.54 (5762.82, 8055.65) | 1385.45 (1029.45, 1816.10) | -79.0608 | 69.31 (60.37, 84.39) | 23.53 (17.48, 30.84) | -3.68 (-4.39, -2.97) |
| Portugal | 1705.45 (1463.53, 2035.80) | 212.18 (149.77, 291.45) | -87.5585 | 80.66 (69.22, 96.29) | 15.23 (10.75, 20.91) | -5.61 (-6.11, -5.11) |
| Puerto Rico | 533.04 (442.78, 661.19) | 125.82 (94.91, 161.94) | -76.3949 | 53.51 (44.45, 66.37) | 24.04 (18.14, 30.95) | -2.66 (-3.46, -1.87) |
| Qatar | 190.66 (132.75, 265.47) | 156.33 (118.23, 202.46) | -18.004 | 153.16 (106.64, 213.25) | 38.05 (28.78, 49.28) | -4.80 (-5.47, -4.12) |
| Republic of Korea | 18147.10 (14690.49, 22095.30) | 1428.73 (1068.47, 1889.29) | -92.127 | 158.69 (128.46, 193.22) | 20.83 (15.58, 27.55) | -6.75 (-6.95, -6.56) |
| Republic of Moldova | 324.53 (251.15, 417.04) | 109.40 (77.29, 150.85) | -66.2913 | 26.35 (20.39, 33.86) | 18.95 (13.39, 26.13) | -1.19 (-1.46, -0.92) |
| Romania | 2314.62 (1606.29, 2961.63) | 731.08 (523.96, 985.19) | -68.4145 | 41.51 (28.80, 53.11) | 24.13 (17.29, 32.52) | -1.78 (-2.22, -1.35) |
| Russian Federation | 12831.07 (10016.62, 15381.27) | 7157.81 (5412.54, 9430.97) | -44.215 | 36.96 (28.85, 44.30) | 26.52 (20.05, 34.94) | -1.24 (-1.52, -0.96) |
| Rwanda | 6290.31 (4076.07, 9648.82) | 2764.95 (1870.43, 3993.01) | -56.0443 | 186.00 (120.53, 285.31) | 57.08 (38.62, 82.44) | -3.90 (-4.77, -3.02) |
| Saint Kitts and Nevis | 16.16 (13.67, 18.98) | 5.09 (4.00, 6.41) | -68.4795 | 115.18 (97.44, 135.33) | 43.10 (33.87, 54.28) | -3.13 (-3.77, -2.49) |
| Saint Lucia | 31.04 (24.96, 37.59) | 9.19 (7.01, 11.68) | -70.3839 | 59.24 (47.65, 71.75) | 28.90 (22.04, 36.74) | -2.46 (-2.79, -2.13) |
| Saint Vincent and the Grenadines | 43.35 (34.29, 53.48) | 10.27 (7.92, 12.95) | -76.3025 | 104.07 (82.31, 128.38) | 41.09 (31.70, 51.81) | -3.14 (-3.82, -2.46) |
| Samoa | 103.34 (74.81, 140.41) | 53.15 (37.75, 72.62) | -48.5704 | 156.89 (113.57, 213.16) | 72.15 (51.25, 98.58) | -2.36 (-2.60, -2.12) |
| San Marino | 1.62 (1.12, 2.21) | 0.83 (0.62, 1.07) | -48.823 | 35.47 (24.42, 48.48) | 15.51 (11.51, 20.08) | -2.75 (-2.90, -2.61) |
| Sao Tome and Principe | 196.30 (140.25, 276.39) | 76.73 (49.86, 118.82) | -60.9134 | 345.01 (246.50, 485.79) | 103.13 (67.01, 159.71) | -3.96 (-5.71, -2.17) |
| Saudi Arabia | 4541.97 (3119.73, 6403.99) | 2322.68 (1635.20, 3153.17) | -48.8619 | 68.08 (46.76, 95.99) | 32.96 (23.21, 44.75) | -2.33 (-2.67, -1.99) |
| Senegal | 12699.83 (8810.32, 17612.48) | 8907.72 (6141.02, 13974.11) | -29.8595 | 348.82 (241.99, 483.75) | 145.98 (100.64, 229.01) | -2.98 (-4.38, -1.56) |
| Serbia | 3655.17 (2567.93, 4866.47) | 402.78 (288.10, 558.60) | -88.9806 | 173.22 (121.69, 230.62) | 27.52 (19.69, 38.17) | -5.92 (-8.15, -3.64) |
| Seychelles | 16.41 (12.54, 20.58) | 10.63 (8.14, 13.36) | -35.2191 | 68.77 (52.57, 86.24) | 48.73 (37.29, 61.26) | -1.40 (-3.27, 0.51) |
| Sierra Leone | 13466.83 (7788.48, 21173.76) | 15485.64 (9447.49, 24518.96) | 14.99094 | 843.75 (487.98, 1326.62) | 463.45 (282.74, 733.80) | -2.09 (-2.43, -1.74) |
| Singapore | 443.65 (360.83, 594.83) | 122.69 (90.99, 159.44) | -72.3454 | 68.32 (55.57, 91.60) | 15.69 (11.64, 20.39) | -4.95 (-6.05, -3.85) |
| Slovakia | 742.29 (600.97, 932.83) | 200.51 (144.69, 271.91) | -72.9871 | 56.02 (45.36, 70.40) | 23.59 (17.02, 31.99) | -3.12 (-4.17, -2.07) |
| Slovenia | 220.27 (179.00, 260.50) | 53.06 (38.12, 70.46) | -75.9101 | 53.27 (43.29, 63.00) | 17.08 (12.27, 22.67) | -3.78 (-4.23, -3.33) |
| Solomon Islands | 387.83 (211.17, 661.60) | 354.91 (230.96, 534.82) | -8.48649 | 246.76 (134.36, 420.95) | 137.39 (89.41, 207.04) | -2.00 (-2.57, -1.43) |
| Somalia | 6296.13 (3639.15, 10003.15) | 10828.56 (7474.57, 15983.69) | 71.98743 | 181.54 (104.93, 288.42) | 113.98 (78.68, 168.25) | -1.55 (-1.91, -1.19) |
| South Africa | 13798.38 (8740.69, 19527.14) | 6105.10 (4667.67, 7926.36) | -55.7549 | 104.19 (66.00, 147.44) | 40.60 (31.04, 52.72) | -3.12 (-3.67, -2.57) |
| South Sudan | 5681.22 (3136.32, 9531.29) | 4568.42 (3109.66, 6628.84) | -19.5874 | 217.79 (120.23, 365.38) | 109.59 (74.60, 159.02) | -2.33 (-2.61, -2.05) |
| Spain | 7470.66 (6655.10, 8619.72) | 1379.89 (1094.69, 1705.99) | -81.5292 | 95.35 (84.94, 110.02) | 20.36 (16.15, 25.17) | -5.22 (-5.66, -4.77) |
| Sri Lanka | 4625.66 (3428.16, 6072.16) | 2124.14 (1511.05, 2826.19) | -54.0793 | 82.45 (61.11, 108.23) | 41.76 (29.70, 55.56) | -2.14 (-2.78, -1.48) |
| Sudan | 79653.06 (35063.53, 158815.59) | 36312.66 (21124.31, 58028.08) | -54.4115 | 877.41 (386.24, 1749.42) | 229.81 (133.69, 367.25) | -4.52 (-4.86, -4.17) |
| Suriname | 265.77 (189.07, 356.70) | 102.58 (72.64, 145.06) | -61.4041 | 203.28 (144.61, 272.83) | 69.95 (49.54, 98.92) | -3.62 (-4.37, -2.86) |
| Sweden | 623.57 (508.00, 774.90) | 493.27 (336.36, 699.43) | -20.8959 | 40.36 (32.88, 50.16) | 27.29 (18.61, 38.70) | -1.34 (-2.34, -0.32) |
| Switzerland | 218.22 (168.71, 275.73) | 130.38 (91.81, 176.50) | -40.2525 | 18.91 (14.62, 23.89) | 9.94 (7.00, 13.45) | -2.17 (-2.59, -1.76) |
| Syrian Arab Republic | 75747.91 (45218.46, 105734.78) | 9084.56 (6948.04, 11556.64) | -88.0069 | 1246.21 (743.94, 1739.56) | 224.27 (171.53, 285.30) | -5.65 (-6.94, -4.33) |
| Taiwan (Province of China) | 4321.35 (3778.94, 4947.93) | 851.32 (620.80, 1115.96) | -80.2997 | 78.32 (68.49, 89.68) | 28.12 (20.51, 36.86) | -3.44 (-4.16, -2.72) |
| Tajikistan | 759.77 (564.19, 954.08) | 612.94 (468.81, 804.33) | -19.3251 | 32.81 (24.36, 41.20) | 18.84 (14.41, 24.73) | -1.93 (-2.28, -1.59) |
| Thailand | 15778.64 (11054.05, 22744.81) | 5766.53 (4513.97, 7321.52) | -63.4536 | 92.77 (64.99, 133.73) | 54.08 (42.33, 68.66) | -1.77 (-2.19, -1.34) |
| Timor-Leste | 821.99 (412.45, 1331.57) | 566.81 (338.53, 817.43) | -31.0439 | 245.69 (123.28, 398.01) | 112.33 (67.09, 161.99) | -2.65 (-3.02, -2.29) |
| Togo | 5565.93 (3864.98, 7790.44) | 4926.85 (3388.54, 7291.19) | -11.482 | 312.88 (217.27, 437.93) | 156.53 (107.65, 231.64) | -2.33 (-2.55, -2.12) |
| Tokelau | 1.09 (0.70, 1.63) | 0.31 (0.22, 0.42) | -71.8391 | 154.75 (99.72, 232.19) | 65.56 (46.72, 90.17) | -2.97 (-3.38, -2.55) |
| Tonga | 41.86 (31.09, 54.49) | 26.86 (19.80, 36.36) | -35.8352 | 105.32 (78.21, 137.09) | 74.86 (55.17, 101.34) | -1.17 (-1.62, -0.72) |
| Trinidad and Tobago | 429.26 (355.40, 511.28) | 121.29 (92.36, 152.06) | -71.744 | 106.23 (87.95, 126.53) | 44.61 (33.97, 55.93) | -3.05 (-3.63, -2.46) |
| Tunisia | 6734.48 (4411.09, 10719.97) | 1171.34 (835.80, 1586.15) | -82.6069 | 211.33 (138.42, 336.39) | 44.27 (31.59, 59.95) | -5.27 (-5.54, -4.99) |
| Turkey | 45843.06 (27828.44, 73942.75) | 6508.24 (5051.16, 8249.07) | -85.8032 | 212.11 (128.76, 342.13) | 41.27 (32.03, 52.31) | -5.45 (-5.78, -5.11) |
| Turkmenistan | 1165.09 (857.64, 1591.19) | 589.72 (426.43, 842.71) | -49.3842 | 77.46 (57.02, 105.79) | 38.88 (28.12, 55.57) | -2.37 (-2.87, -1.88) |
| Tuvalu | 15.19 (8.52, 26.40) | 3.09 (2.24, 4.29) | -79.6665 | 460.50 (258.33, 800.31) | 90.33 (65.38, 125.54) | -5.39 (-5.71, -5.07) |
| Uganda | 11140.38 (7212.36, 16549.13) | 13042.18 (9220.91, 18379.66) | 17.07119 | 132.06 (85.50, 196.18) | 68.36 (48.33, 96.34) | -2.29 (-2.71, -1.86) |
| Ukraine | 3343.12 (2412.70, 4405.40) | 2306.64 (1665.08, 3107.06) | -31.0035 | 29.49 (21.28, 38.85) | 33.39 (24.11, 44.98) | 0.46 (0.12, 0.81) |
| United Arab Emirates | 1645.52 (1005.60, 2298.37) | 605.78 (442.53, 817.04) | -63.186 | 278.38 (170.12, 388.83) | 51.68 (37.75, 69.70) | -5.75 (-6.11, -5.39) |
| United Kingdom | 5115.49 (4517.60, 5932.08) | 1958.87 (1493.81, 2527.27) | -61.707 | 46.70 (41.24, 54.15) | 16.53 (12.61, 21.33) | -3.55 (-3.82, -3.29) |
| United Republic of Tanzania | 23823.22 (14953.00, 36003.33) | 24956.79 (15718.82, 39263.90) | 4.758245 | 196.28 (123.20, 296.63) | 101.56 (63.96, 159.78) | -2.13 (-2.35, -1.91) |
| United States of America | 35863.39 (32536.32, 40224.33) | 24541.01 (20208.50, 29754.02) | -31.5709 | 64.45 (58.47, 72.29) | 40.80 (33.59, 49.46) | -1.56 (-2.13, -0.99) |
| United States Virgin Islands | 13.72 (10.45, 17.92) | 3.48 (2.61, 4.50) | -74.6234 | 43.11 (32.84, 56.29) | 17.39 (13.06, 22.47) | -3.10 (-3.75, -2.45) |
| Uruguay | 618.35 (527.56, 720.10) | 196.30 (153.71, 243.81) | -68.2541 | 75.56 (64.46, 87.99) | 27.99 (21.91, 34.76) | -3.51 (-4.79, -2.21) |
| Uzbekistan | 2012.43 (1593.35, 2509.69) | 1843.76 (1336.82, 2484.85) | -8.38138 | 23.49 (18.60, 29.29) | 17.91 (12.98, 24.13) | -0.93 (-1.05, -0.80) |
| Vanuatu | 131.40 (85.41, 185.98) | 194.26 (128.50, 283.98) | 47.84522 | 196.39 (127.66, 277.98) | 175.26 (115.94, 256.21) | -0.33 (-1.02, 0.37) |
| Venezuela (Bolivarian Republic of) | 4120.09 (3479.26, 4792.20) | 2099.57 (1632.28, 2691.39) | -49.0406 | 57.95 (48.94, 67.40) | 29.82 (23.18, 38.22) | -2.24 (-3.17, -1.30) |
| Viet Nam | 42688.33 (25766.23, 64666.79) | 13134.62 (9373.07, 18768.97) | -69.2314 | 162.96 (98.36, 246.85) | 61.60 (43.96, 88.03) | -3.28 (-3.51, -3.06) |
| Yemen | 30899.07 (16334.96, 66554.17) | 19022.11 (12552.64, 27742.73) | -38.4379 | 430.26 (227.46, 926.74) | 146.92 (96.95, 214.28) | -3.64 (-3.87, -3.41) |
| Zambia | 8145.01 (4925.51, 12681.72) | 5064.26 (3581.44, 7057.73) | -37.8237 | 216.51 (130.93, 337.11) | 65.48 (46.31, 91.26) | -4.03 (-4.43, -3.62) |
| Zimbabwe | 3320.62 (2081.24, 4902.17) | 4978.92 (3208.53, 7460.34) | 49.93924 | 68.92 (43.20, 101.75) | 83.45 (53.78, 125.05) | 0.76 (0.43, 1.09) |

Note: ASR: age-standardized rates; AAPC, average annual percentage change; CI, confidence interval; DALYs, disability-adjusted life-years; UI, uncertainty interval


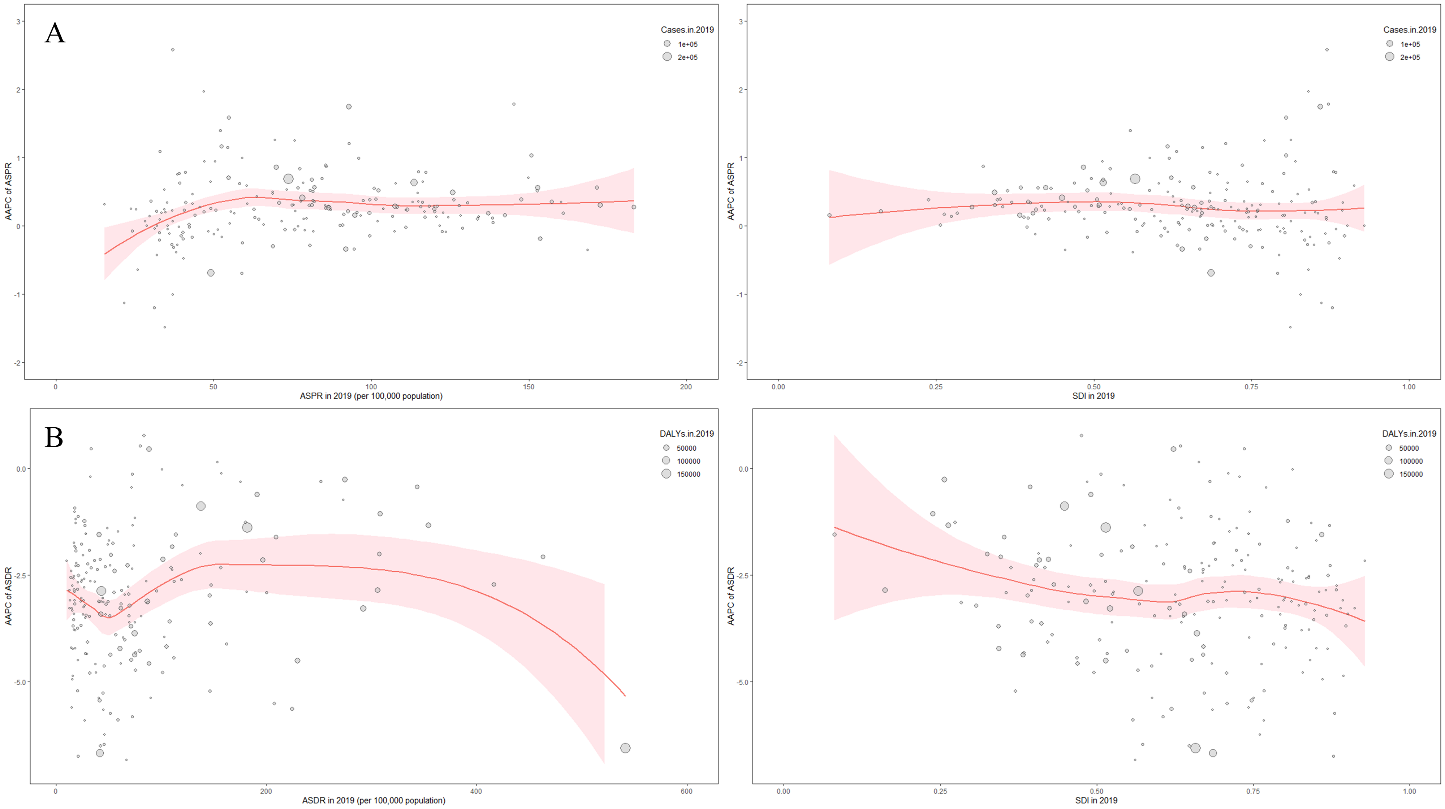


Figure S1. Average annual percentage changes of age-standardized prevalence rates and age-standardized DALY rates of stroke at the country and territorial levels.

(A) Correlation of average annual percentage changes with age-standardized prevalence rates and sociodemographic indexes in 2019.

(B) Correlation of average annual percentage changes with age-standardized DALY rates and sociodemographic indexes in 2019.

The prevalent strokes and DALYs due to stroke from 204 countries and territories are represented by the circles. The circle size reflects the number of prevalent strokes and DALYs due to stroke. AAPC, average annual percentage change; ASPR, age-standardized prevalence rate; ASDR, age-standardized DALY rate; SDI, sociodemographic index.
